# Design, synthesis of some novel coumarins and their nanoformulations into lipid-chitosan nanocapsule as unique antimicrobial agents

**DOI:** 10.1038/s41598-024-79861-7

**Published:** 2024-12-23

**Authors:** Ibrahim Taha Radwan, Ibrahim M. El-Sherbiny, Abdelfattah M. Selim, Nadia Hanafy Metwally

**Affiliations:** 1 Supplementary General Sciences Department, Faculty of Oral and Dental Medicine, Future University in Egypt, Cairo, 11835 Egypt; 2 Center for Materials Science (CMS), Zewail City of Science and Technology, 6th October City, 12578 Giza, Egypt; 3 Department of Animal Medicine (Infectious Diseases), College of Veterinary Medicine, Benha University, Toukh, 13736 Egypt; 4 Chemistry Department, Faculty of Science, Cairo University, Giza, 12613 Egypt

**Keywords:** Coumarins, Antimicrobial properties, Nanostructure lipid carrier, Chitosan nanocapsule, DNA-Gyrase assay, TEM, Biochemistry, Microbiology, Chemistry

## Abstract

Developing and creating novel antibiotics is one of the most important targets in treating infectious diseases. Novel coumarins were synthesized and characterized using different spectroscopic techniques such as Fourier Transform Infrared (FTIR), Nuclear magnetic resonance^1^H and ^13^C and mass spectroscopy (MS). All of the synthesized compounds have been tested for activity and sensitivity against the microbial strains of *B. subtilis*,* S. aureus*,* E. coli*,* P. aeruginosa*,* S. typhi*, and *C. albicans*. All compounds showed substantial results against the tested microbes except *S. typhi*, which was not affected in any way by these coumarins. Exceptional results were shown by compounds **4**, **6d**, and **8b**, which made them the best candidates for loading to the vicinity of nanostructure lipid carrier and coated by chitosan nanocapsule (NLC-Cs). Transmission electron microscope (TEM) confirmed spherical morphology with particles size less than 500 nm. Also, dynamic light scattering (DLS) were utilized to measure the average particle size (between 100 and 200 nm) and the stability assessed by zeta potential were found to be more positive confirming the chitosan encapsulation. Antimicrobial activity assessments were performed for both synthetic compounds and their NLCs analogues. The nanoformulation of **4**-NLC-Cs, **6d**-NLC-Cs, and **8b**-NLC-Cs manifested unique biological results, especially **8b**-NLC-Cs, which revealed powerful effects over all the tested organisms including *S. typhi*. The increasing biological effect of the drugs in their nanoscale form is reflected in the increasing value of inhibition zone diameter and suppressing the value of MIC to reach record levels like **8b**-NLC-Cs disclosed MIC = 0.48 and 0.24 µg/ml against *S. aureus* and *C. albicans*, respectively, by the mean **8b**-NLC-Cs nanoformulation suppressed the MIC by 65 folds of its initial value before nano. In continuation, it was proven that the compounds **4**, **6d** and **8b** were found to make noticeable changes on the DNA-Gyrase levels with reduced IC_50_ values particularly **8b** showed excellent inhibitory effect with IC_50_ = 4.56 µM. TEM was used to pursue the morphological changes that occur in bacterial cells of *P. aeruginosa*. The weakness of the cell wall in most bacterial cells treated with nanomaterials, **8b**-NLC-Cs, has reached the point of the cell wall rupture and the cell components spilling out of the cells causing necrotic cell death.

**Supplementary Information:**

The online version contains supplementary material available at 10.1038/s41598-024-79861-7.

## Introduction

Numerous synthetic compounds with unique biological properties became less useful because of their insoluble nature, which made it challenging to handle those utilized in normal methods for example converting them into salts due to their lacked acidic or basic functional groups. This makes synthetic chemistry researchers handcuffed, and these materials are forgotten and ignored despite their potential biological properties at the laboratory and in *vitro* scale. Due to several limitations of drug administration, such as low bioavailability, drug metabolism, associated toxicity, undesirable side effects, prolonged treatment time, and effects on the patient`s psyche, nanotechnology and drug delivery technologies are steadily increasing over traditional techniques. Most of these problems come from the hydrophilic and hydrophobic characteristics of drugs, which have promoted the creation of unique nanocarriers to fulfill loading objectives and reduce drug administration problems. The development of an effective nano delivery scaffold is one of the necessary goals of a drug delivery system to minimize drug disintegration as a cause of early degradation and maintain the drug’s optimal dosage in the target tissue. In addition, the desired treatment results can be achieved by minimizing side effects^1^. Lipid and polymeric nanoparticles are two different classes of unique nanocarriers used for drug encapsulation^2^. Solid Lipid Nanoparticles (SLN) and Nanostructured Lipid Carriers (NLC) are two different generations of lipid nanoparticles (LNPs). While their chemical constituents are similar, the main difference between them is that the lipid matrix is made up of one or more liquid lipids, in addition to solid lipids. Many vesicles were created, particularly after the discovery of nonionic surfactants to accomplish unique drug-loading systems such as liposomes^3^, niosomes^4^, ethosomes^5^, herbosomes^6^, bilosomes^7^, colloidosomes^8^, sphingosomes^9^, and transferosomes^10^.

Antibiotics have played an important role in controlling the spread of pathogenic microorganisms. However, the emergence of antibiotic-resistance bacteria has provided opportunities for the generating of new strains of pathogens, and the lack of appropriate treatments has led to infectious diseases and increased morbidity and mortality in modern society^11^. The only way out of this predicament is to keep discovering new ways to eliminate this enemy and to be prepared with unconventional alternatives such as natural polymers and their nanoformulations.

Chitosan, a marine-derived biopolymer has attracted much attention recently due to its important antibacterial properties and nontoxicity, biodegradability, and biocompatibility^12^. Chitosan has been synthetically modified with different functional groups to obtain biologically enhanced derivatives. The most common hydrophilic functional groups are quaternary ammoniumyl, carboxyalkyl, guanidinyl, hydroxyalkyl groups, and thiol-containing groups, while hydrophobic groups are long alkyl chains, substituted phenyl, and benzyl rings. These antibacterial chitosan derivatives are widely used in a range of fields, as evidenced by several recent publications. Chitosan has been shown to have antibacterial properties against Gram-positive, Gram-negative bacteria, and antifungal properties. Notably low-molecular-weight chitosan hydrogels have shown strong antibacterial activity against biofilms of various Candida species^13^.

**Fig. 1:**
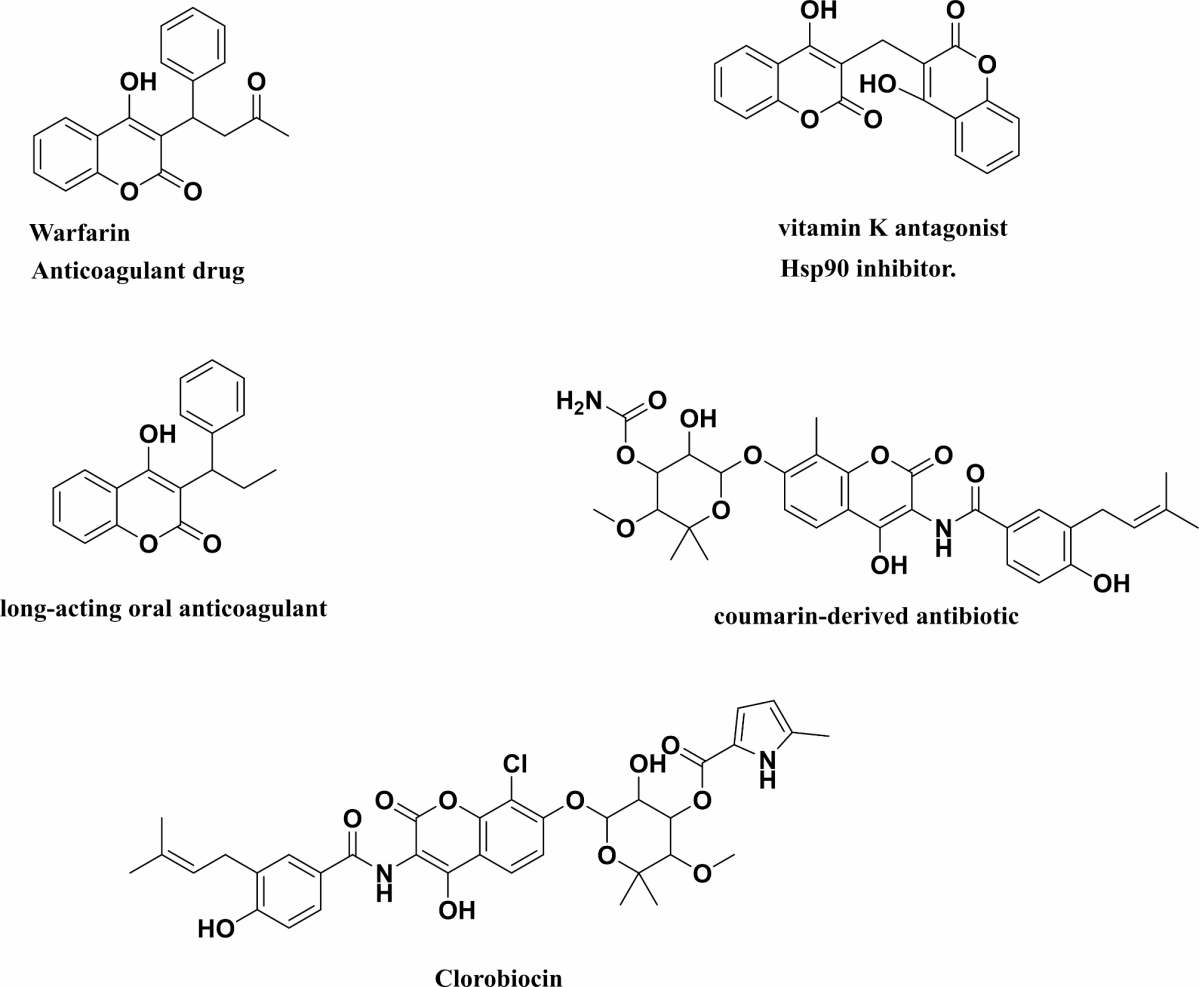
Different coumarin-based drugs.

However, the chemical biology toolsets need to be updated from time to time to keep track of changes that occur in biological systems related to human health and sometimes need urgent real out of box thinking for significant progress and growth of beneficial synthetic changes that could be tackled to get drug development. The synthetic yields of some natural products were found to manifest assorted biological applications. Coumarin heterocyclic is one of the most important nuclei that contains oxygen and plays a crucial role in not only designing new molecular therapeutics^14–17^ but also drug markets (Fig. 1). For the first time, the parent coumarin synthesized by Vogel from Tonkabeansin at 1820^18^ from this moment coumarin chemistry has been intensely the focus of attention due to its widespread biological activities, especially as anticancer^19,20^, anti-HIV^21^, analgesic^22^, antiplatelet^23,24^, antiviral^25,26^, antibacterial^27^, antimalarial^28^, anti-filarial^29^and other purposes in photodynamic therapy^30^.

Topoisomerases are categories of enzymes that are responsible for making changes in the DNA’s topology and they can control the interconvert process between relaxed and supercoiled forms of DNA^31,32^. They play an important role in controlling DNA replication, transcription, recombination repair, and chromosome de-condensation^33,34^. Topoisomerase inhibitors idea is based on, the discontinuity of the ligament steps in the microbial for both gram-positive and negative bacteria cell cycle that generates single and double-stranded breaks that make the genomic structure lose its integrity leading to apoptosis and cell death in the proliferating cell^35^. DNA gyrase is a tetrameric (four components) enzyme composed of two main gyrase subunits expressed as GyrA and GyrB.

Structurally the complex is formed by 3 pairs of “gates”, each gate participates in the DNA relaxation and is involved in the segregation of DNA after replication, initiation of DNA replication, and gene expression. It is one of the most investigated and validated targets for the development of new antibacterial agents. Its absence in the mammalian organism and its crucial role in the bacterial DNA replication cycle make this enzyme a suitable target for the development of antibacterial drugs with selective toxicity. It comprises two subunits; Gyrase A and Gyrase B that together form the catalytically active heterotetrameric enzyme (i.e. A2B2). The role of the A subunit is breakage and reunion of the double DNA strand, while the B subunit (DNA gyrase B) possesses the ATPase activity, providing a sufficient amount of energy for the DNA supercoiling^36–38^. Hence these enzymes serve as attractive targets for designing new antibacterial drugs.

Novobiocin, a naturally occurring antibiotic produced by the bacterium *Streptomyces niveus*, has been the subject of significant interest in antibiotic research and development. Novobiocin’s chemical structure has been extensively studied to identify potential modifications that can improve its pharmacological properties. Researchers have explored the synthesis of novel derivatives with enhanced potency and reduced toxicity. Additionally, the structural features of novobiocin have been crucial in understanding its mechanism of action, shedding light on how it disrupts bacterial processes. The chemical structure and properties of novobiocin play a pivotal role in shaping its antibiotic activity and drive ongoing research efforts to optimize its therapeutic potential. It belongs to the coumarin family and consists of a fused-ring system with a lactone moiety, along with several functional groups such as carbonyl, hydroxyl, and carboxylic acid. This complex structure allows novobiocin to interact with specific enzymes in bacteria, inhibiting their activity and ultimately leading to bacterial cell death. Here on we tried to synthesize coumarin-based drugs making use of the structure of novobiocin as a parent drug. On continuing our research in the synthesis of active moieties^39–52^ and drug delivery applications^53–55^we look forward to making use of the two different scopes of synthesis and drug delivery for the synthesis of a novel series of coumarin-based compounds and their chitosan-nanostructured lipid carrier conjugate nanoparticles as promising antimicrobial agents.

**Fig. 2:**
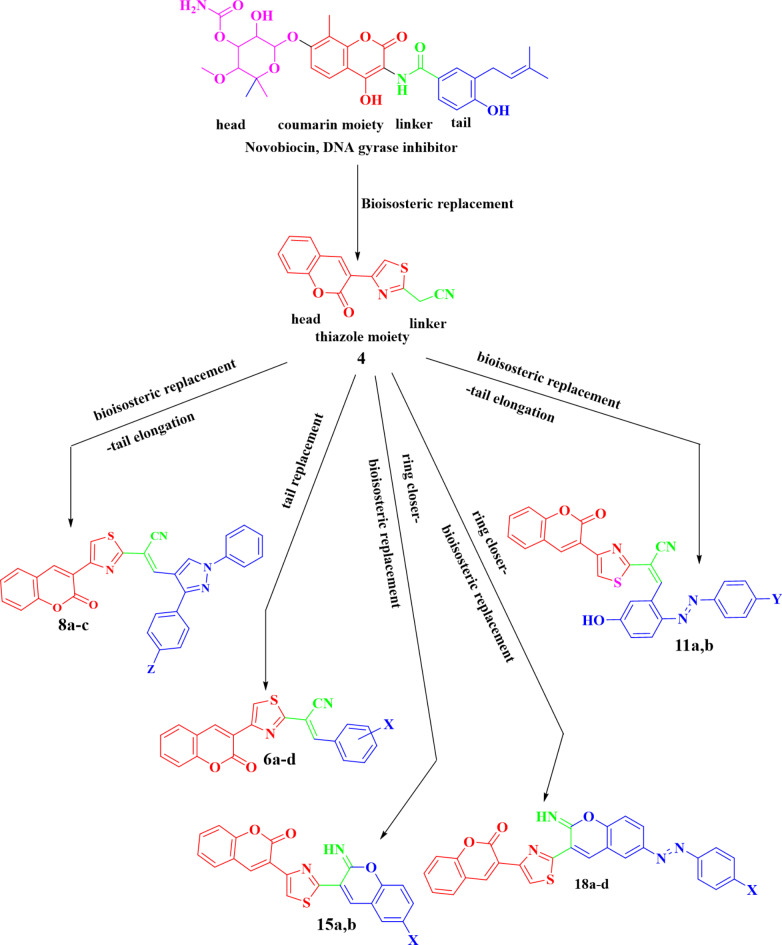
Bioisosteric drug design tactic for the synthesis of coumarin derivatives.

Bioisosteric tactic was used to synthesize coumarin-based drugs that share the same structural features as novobiocin (Fig. 2). The moiety of 8-methyl-4-hydroxy coumarin was replaced with coumarin thiazole system **4**. Hopefully, the combination between thiazole and coumarin will achieve the desired biological outcomes. Making use of the newly generated active methylene group, multiple synthetic routes were designed through different drug-design pathways. The arylidene derivatives of **6a-d** were designed to be optionally prepared using both thermal and/or green Knoevenagel reaction with aromatic aldehyde synthesis *via* tail elongation protocol, similarly, compounds **8a-c** were sketched in addition to tail elongation, the bioisosteric replacement was applied to change the active methylene group in the starting **4** to pyrazole group. The Synthetic route of arylazo derivatives **11a**,** b** also proceeded using two alternative pathways *via* tail elongation and bioisosteric replacement of synthon **4** and the corresponding azo dye coupler. 4-Hydroxybenzaldehyde and 2,4-dihydroxybenzaldehyde reacted with the starting **4** through bioisosteric replacement followed by ring closure of the arylidene derivatives depicting **15a**,** b** derivatives. Analogously, 3-hydroxybenzaldehyde undergoes multistep diazotization bioisosteric replacement and come behind the ring closure reaction furnishing **18a-d** derivatives.

## Results and discussion

### Chemistry

**Scheme 1:**
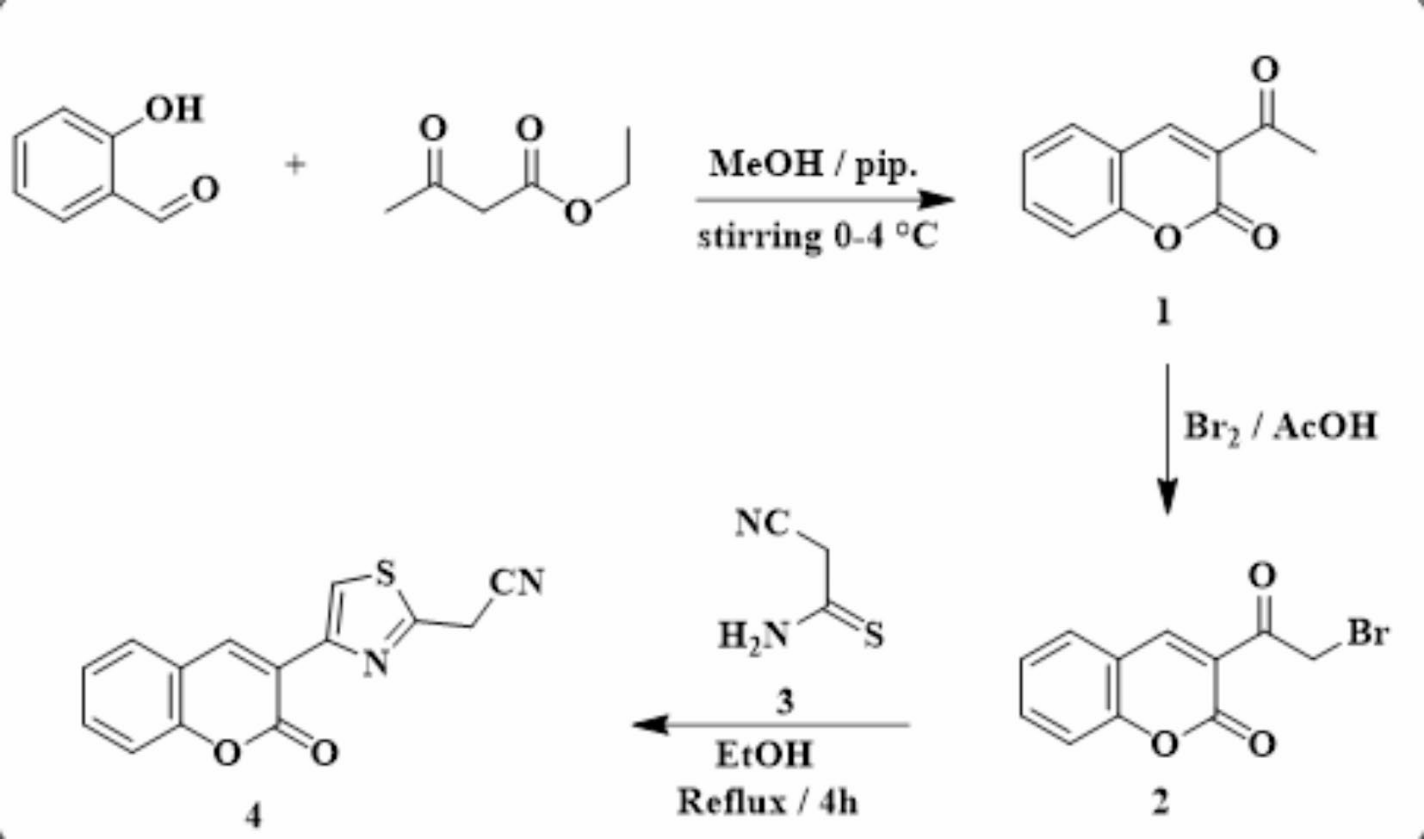
Synthetic route of 2-(4-(2-oxo-2*H*-chromen-3-yl) thiazol-2-yl) acetonitrile **4**.

Bromination of 3-acetyl-2*H*-chromen-2-one **1** with bromine in acetic acid afforded 3-(2-bromoacetyl)-2*H*-chromen-2-one **2** (Scheme 1). Refluxing compound **2** with 2-cyanothioacetamide **3** in absolute ethanol afforded the 2-(4-(2-oxo-2*H*-chromen-3-yl)thiazol-2-yl)acetonitrile **4** (Scheme 1). The structure of **4** was supported by spectroscopic techniques. For example, the IR spectrum showed an absorption band for the nitrile group at a wavelength of 2253 cm^−1^ and an absorption band for the carbonyl function at 1739 cm^−1^. The^1^H NMR chart also presented three singlet signals at δ = 4.64, 8.74, and 8.75 ppm for CH_2_, CH = coumarin, and CH = thiazole ring. In mass spectrum, a molecular ion peak was observed at m/z = 268.23 [M^+^, 100%], which is assigned with its molecular formula C_14_H_8_N_2_O_2_S.

**Scheme 2:**
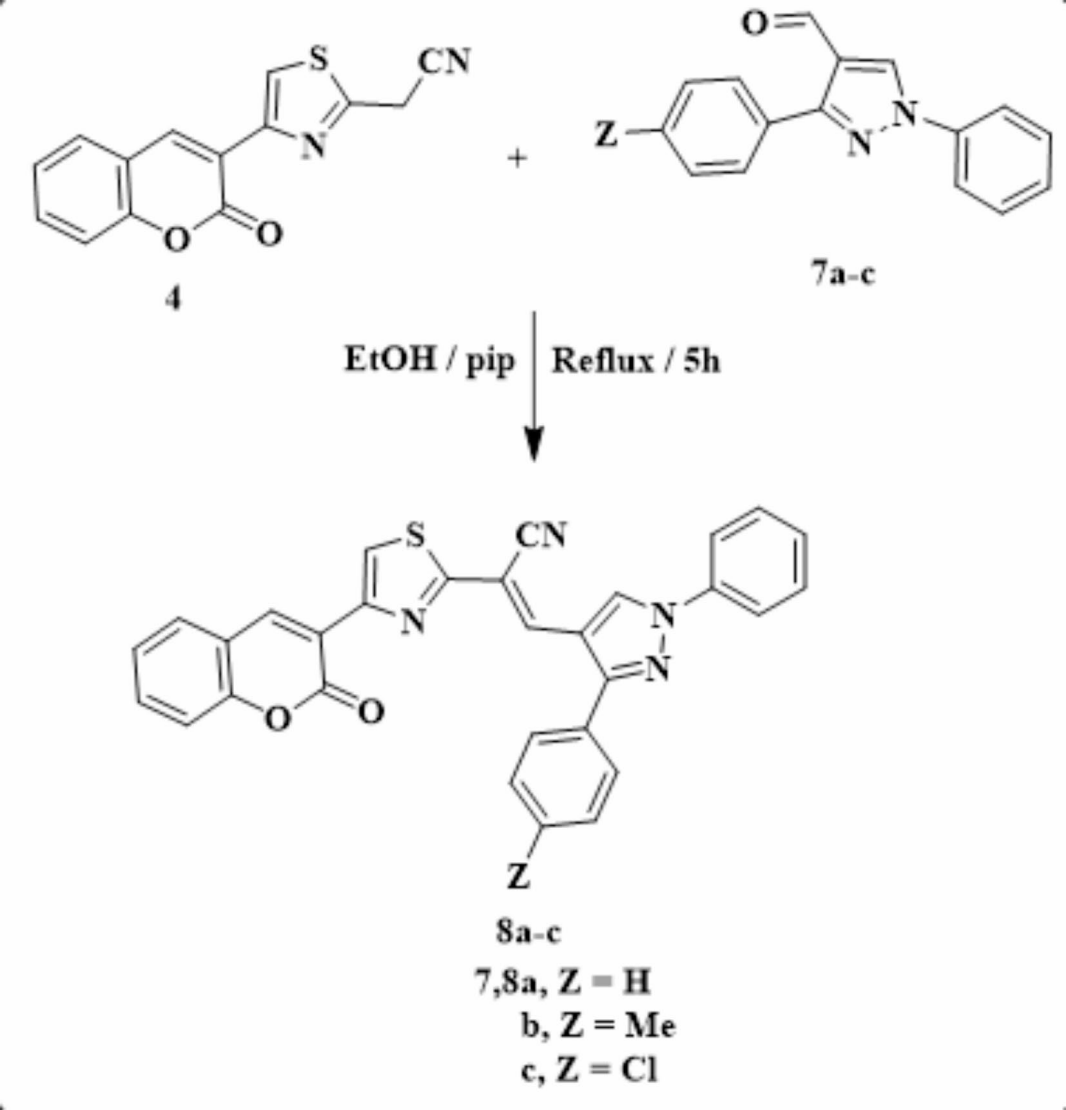
Synthesis of arylmethylene derivatives **8a–c**.

Candidate **4** was used as a precursor for some new heterocyclic compounds so; condensation of compound **4** with aromatic aldehydes **5a-d** under reflux in ethanol containing a catalytic amount of piperidine furnished the arymethylene derivatives **6a-d** (Scheme 2). The structure of products **6a-d** was identified from their spectral data. As an example, compound **6d** exhibited in IR absorption bands of CN and CO at ν 2216 and 1717 cm^−1^, respectively^1^. H NMR spectrum for **6d** revealing several multiplet signals at 7.14 to 7.49 corresponding to two aryl protons, and 7.29 to 7.70 ppm attributed to the three aryl protons, in addition to an olefinic proton and aryl protons in the region of 7.94 to 8.12 ppm.

**Scheme 3:**
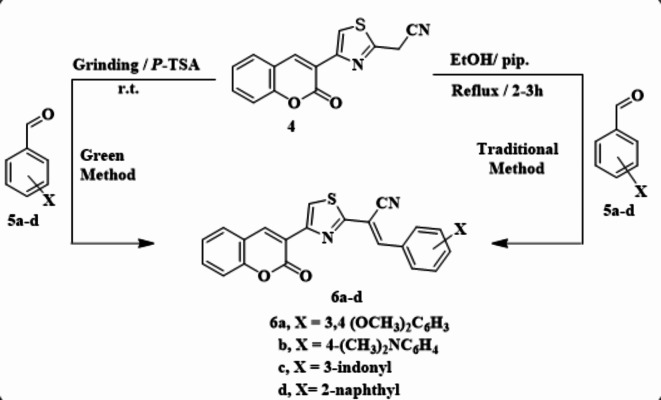
The Synthesis of aryl methylene derivatives **6a–d**.

The mass spectrum of **6d** exhibited a molecular ion peak at m/z = 406.23 (M^+^), consistent with the molecular formula C_25_H_14_N_2_O_2_S. Arylmethylenes **6a-d** can be obtained by the green protocol. Thus, grinding compound **4** with aromatic aldehydes **5a-d** in the presence of *p*-toluene sulfonic acid (*p*-TSA) at room temperature for 20 min providing the products with all properties of **6a-d** (Scheme 3).

Further, the condensation of compound **4** with pyrazole-4-carbaldehydes **7a-c** in ethanol containing a catalytic amount of piperidine furnished the arylmethylene derivatives **8a-c** (Scheme 2). From the IR spectrum of **8b**, absorption bands at ν = 2213 and 1727 cm^−1^ were observed corresponding to cyano and carbonyl groups, respectively^1^. H NMR revealed a singlet signal at 2.42 ppm attributed to the methyl proton and a multiplet signal at 7.40–7.95 ppm involving the aryl protons. In addition, four singlet signals attributed to olefin, thiazole, and coumarin. Pyrazole protons were observed at 8.27, 8.48, 8.72, and 9.17 ppm, respectively. Additionally, characteristic signals owing to methyl, nitrile, and carbonyl carbons were appeared at 21.41, 115.42, and 140.40 in the^13^C NMR spectrum. Furthermore, its mass spectrum showed m/z = 512.29 (M^+^), corresponding to the molecular formula C_31_H_20_N_4_O_2_S.

**Scheme 4:**
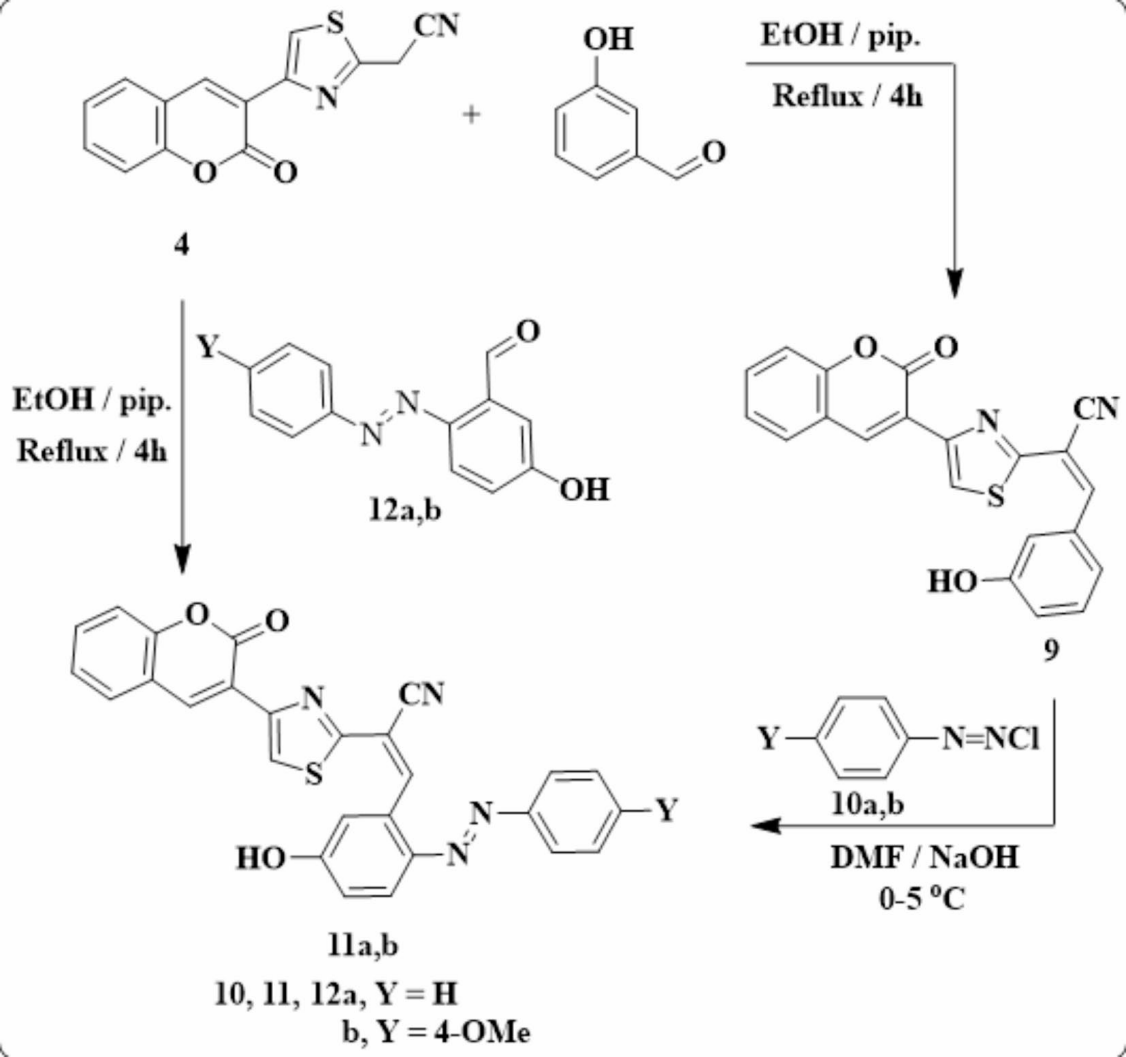
Synthetic route of arylazoderivatives **11a**,** b**.

Arylmethylene derivative **9** was formed by condensation of compound **4** with 3-hydroxybenzaldehyde under reflux in ethanol using a catalytic amount of piperidine (Scheme 4)^1^. H NMR spectrum of **9** appeared a multiplet signal for aryl and olefinic protons in the region of 7.15 to 8.92 ppm. A *D*_*2*_*O* exchangeable singlet signal was observed at 10.30 ppm for OH. IR chart of **9** exhibited absorption bands at ν 3412, 2216, and 1721 cm^−1^ for OH, CN, and CO groups. Its^13^C NMR spectrum also exhibited signals at 115.4, 116.3, 119.6, 120.6, 121.6, 123.8, 124.4, 125.2, 129.2, 129.6, 132.3, 132.6, 139.7, 147.3, 153.0, 153.2, 153.6, and 159.3. The latter compound was coupled with aryldiazonium chlorides **10a**,** b** in *N*,* N*-dimethylformamide (DMF) containing solid sodium hydroxide to afford arylazo derivatives **11a**,** b** (Scheme 2). The structures of **11a**,** b** were confirmed by spectral data. For example, the IR spectrum of compound **11b** exhibited absorption peaks at ν 3910, 2220, and 1723 cm^−1^ for the hydroxyl, nitrile, and carbonyl groups, respectively. A characteristic singlet signal at δ = 3.87 ppm is presented in^1^H NMR for methoxy proton, along with a *D*_*2*_*O* exchangeable singlet signal of OH at 9.10 ppm.

Another synthetic route for the formation of compounds **11a**,** b** proceeds by the reaction of **4** and 4-arylazo-3-hydroxybenzaldehydes **12a**,** b** with a catalytic amount of piperidine under reflux in ethanol, providing compounds similar in all aspects to compounds **11a**,** b** (Scheme 4).

**Scheme 5:**
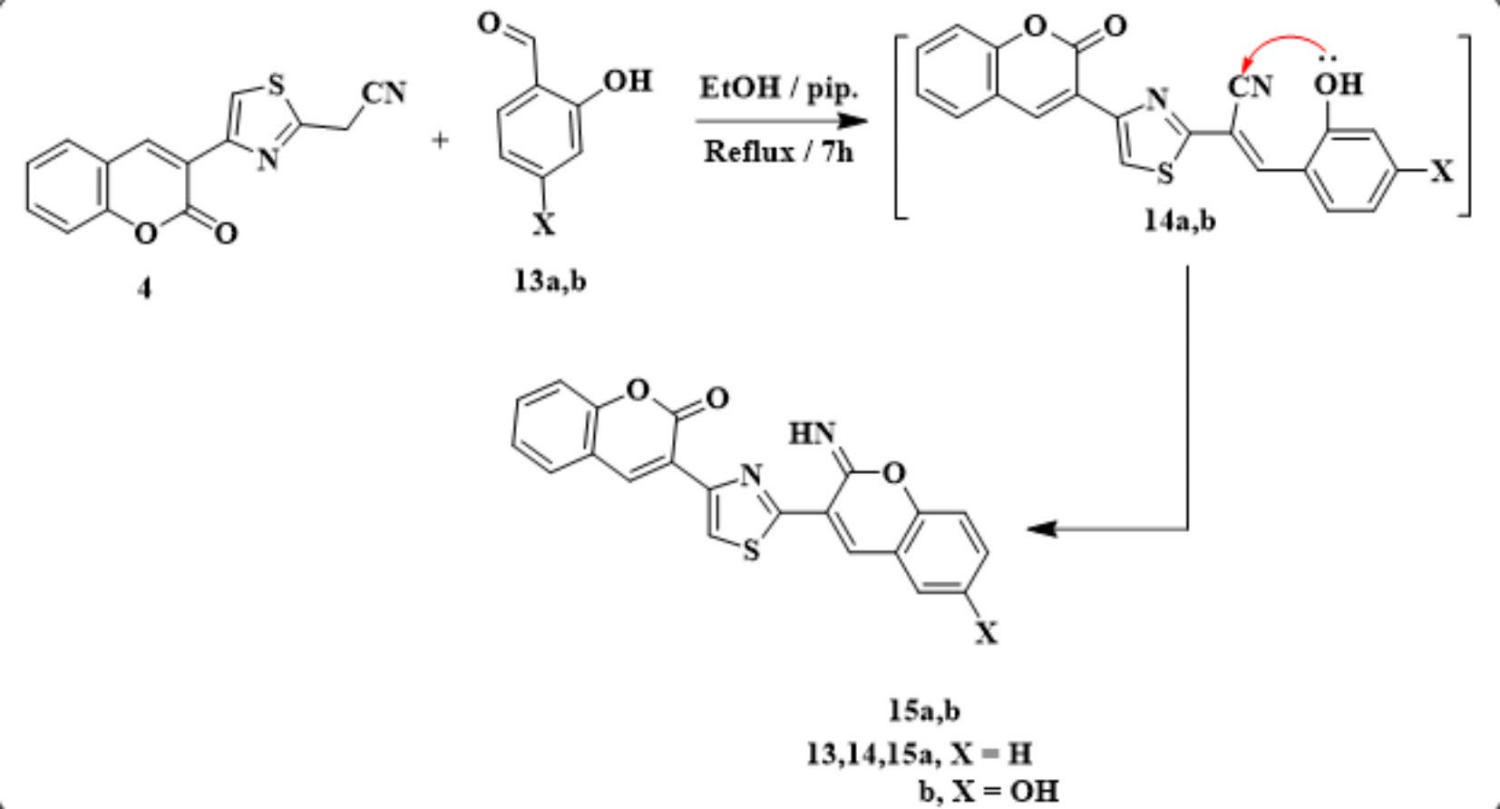
Synthetic way of aeylazo derivatives of **15a**,** b**.

Furthermore, condensation of **4** with salicylaldehyde derivatives **13a**,** b** in refluxing ethanol concerning a few amount of piperidine afforded the chromine derivatives **15a**,** b** (Scheme 5). Spectroscopic techniques were used to confirm the structure of compounds **15a**,** b**^1^. H NMR of **15a** showed a *D*_*2*_*O* exchangeable singlet signal at 10.54 ppm for NH proton, in addition to a multiplet signal at δ = 7.01–8.14 ppm involving aromatic protons. Absorption peaks at wavelength 3233, and 1723 cm^−1^ presented in its IR spectrum for NH and CO functions. The formation of **15a**,** b** proceeded by condensation with the elimination of water molecule, followed by the addition of hydroxyl group on the nitrile function to afford the final arylazo derivatives **15***via* the intermediate **14** (Scheme 5).

**Scheme 6:**
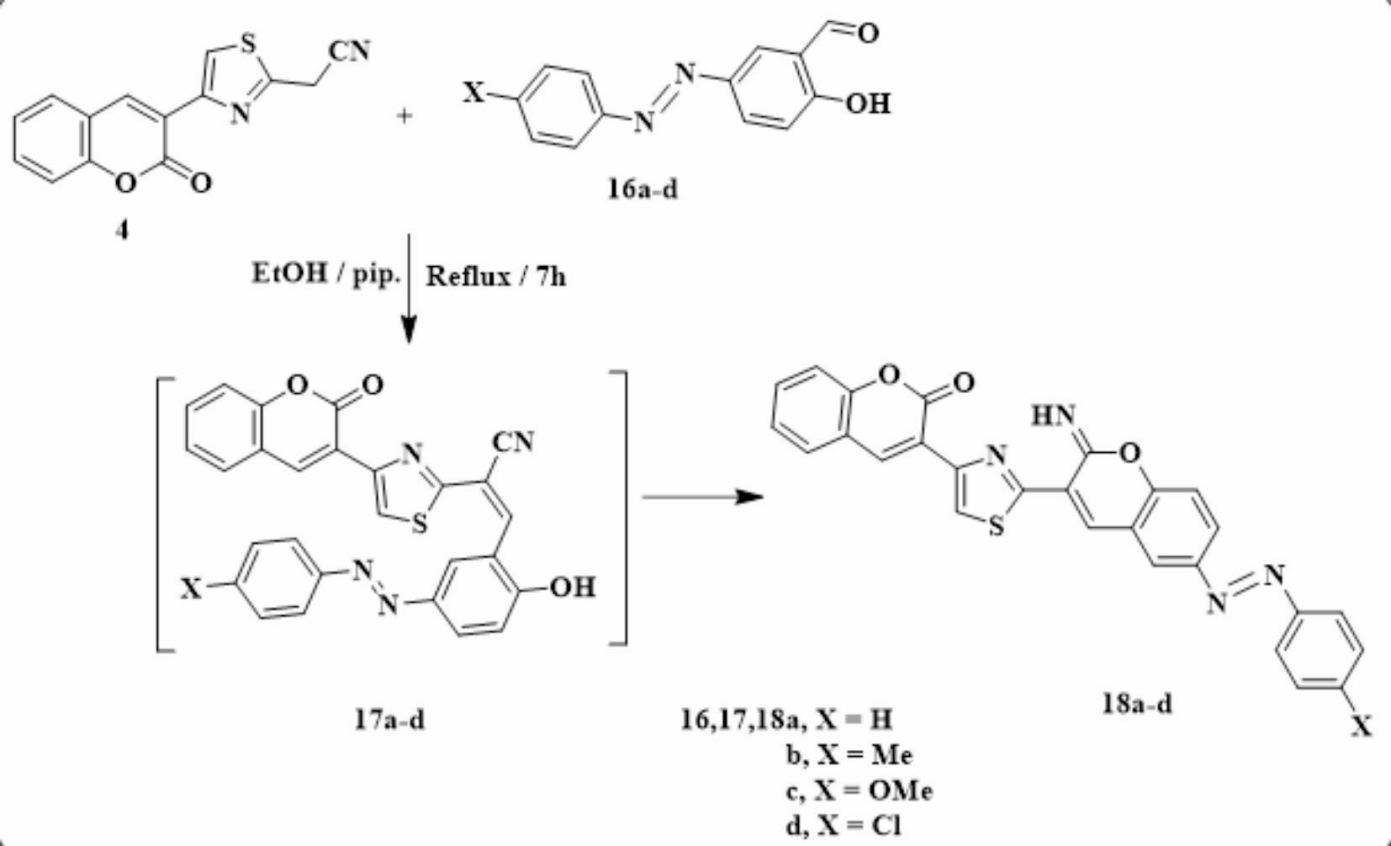
Synthetic way of arylazo derivatives of **18a-d**.

Similarly, condensation of **4** with arylazo salicyalaldehydes **16a-d** in refluxing ethanol with a few amount of piperdine resulted in arylazo derivatives **18a-d** (Scheme 6). The structure of compounds **18a-d** was approved by spectroscopic analysis^1^. H NMR of **18b** exhibited a singlet signal at 2.43 ppm due to the methyl proton. Additionally, a *D*_*2*_*O* exchangeable singlet signal appeared at 9.30 ppm for NH. Absorption bands at ν = 3310 and 1723 cm^−1^ were observed for NH and CO groups in its IR spectrum. Moreover, its mass spectrum showed m/z = 489.31 (M^+^-1), corresponding to the molecular formula C_28_H_18_N_4_O_3_S. Compounds **18a-d** formation proceeded by a condensation pathway, followed by the hydroxyl group was added to the CN group to yield the final arylazo derivatives **18a-d** through the intermediate **16** (Scheme 6).

### Coumarin derivatives encapsulated into lipid-chitosan nanocapsule  (NLC-Cs)

#### Droplet size and zeta potential

**Fig. 3:**
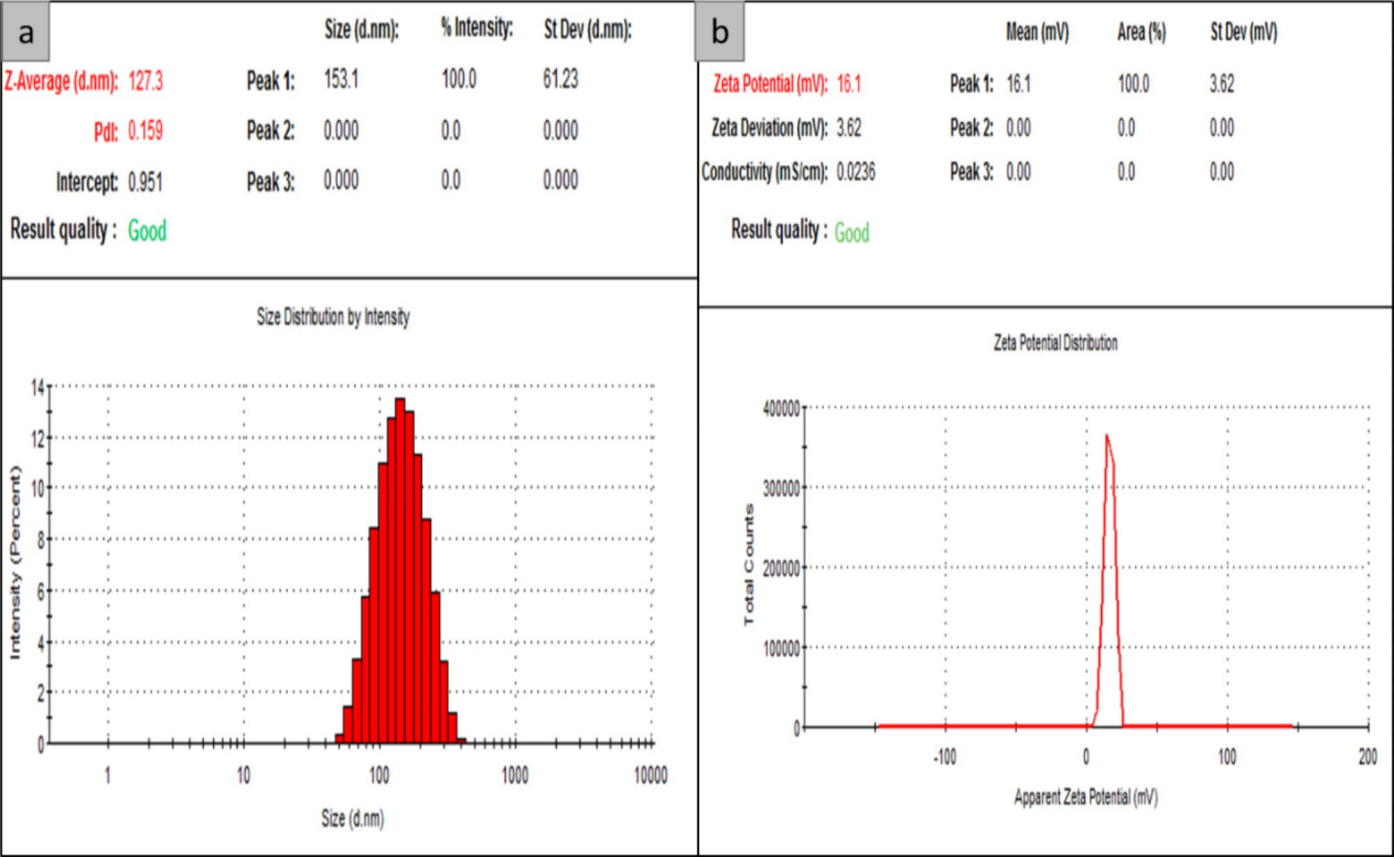
DLS and Zeta potential of the free NLC-Cs nanoformulation (without coumarin derivatives).

According to Fig. 3, the synthesized nps of NLC-Cs and **8b**-NLC-Cs displayed particle sizes of 127.3 and 184.5 nm while PDI were 0.15 and 0.298 when z.p recorded 16.1 and 14.5mV, respectively. The Dynamic Light Scattering (DLS) technique evaluates the Brownian motion of the vesicles or dispersed particles. The kinetic activity of dispersed particles in a liquid is described as random movement in all directions encouraging unbalanced collisions of particles of the dispersed phase (particles or particles) by molecules of the dispersion medium (solvent).

Consequently, an amount of energy is transferred, and works as a movement inducer. The transferred energy is constant and has a higher effect on smaller particles (Smaller move faster than large ones). Achieving enough dispersion of the microemulsion system is mandatory to diminish the multiple scattering effect of Brownian motion^56^. Enough dispersion will encourage small size particles which have a greater surface area than large ones, thus more surfactant is required to satisfy full coverage cover to this greater area^57^. For this reason, the unloaded NLC-Cs exhibited small size diameter of (127 nm) and the encapsulated **8b**-NLC-Cs showed particle size of (184 nm), respectively with little increase in size particle size of **8b**-NLC-Cs due to encapsulation of the hydrophobic **8b** need further addition of surfactant to follow up the increase in surface area and to get smaller sizes.

To identify whether the nanoformulation is homogenous or heterogeneous, PDI is used, poly dispersity index* is defined as when a collection of particles have the same shape, size, and mass. They are known to be uniform or homogenous. Numerically, PDI = Mw/Mn equals the ratio average molecular weight to the number average molecular weight. Smaller ratio means more homogeneity and narrow distribution^58^. The PDI values ranged from (0) for monodispersed or monomodal to (1) for polydisperse size distribution^59^. PDI values of non-encapsulated NLC-Cs and **8b**-NLC-Cs were between 0.1 and less than 0.3 confirming that the designated nps displayed poly dispersed size distribution. However, there is a slight increase in PDI’s value of **8b**-NLC-Cs, it is still below (0.3) and accepted to share some type of homogeneity in their population^60–62^.

#### Zeta potential

**Fig. 4:**
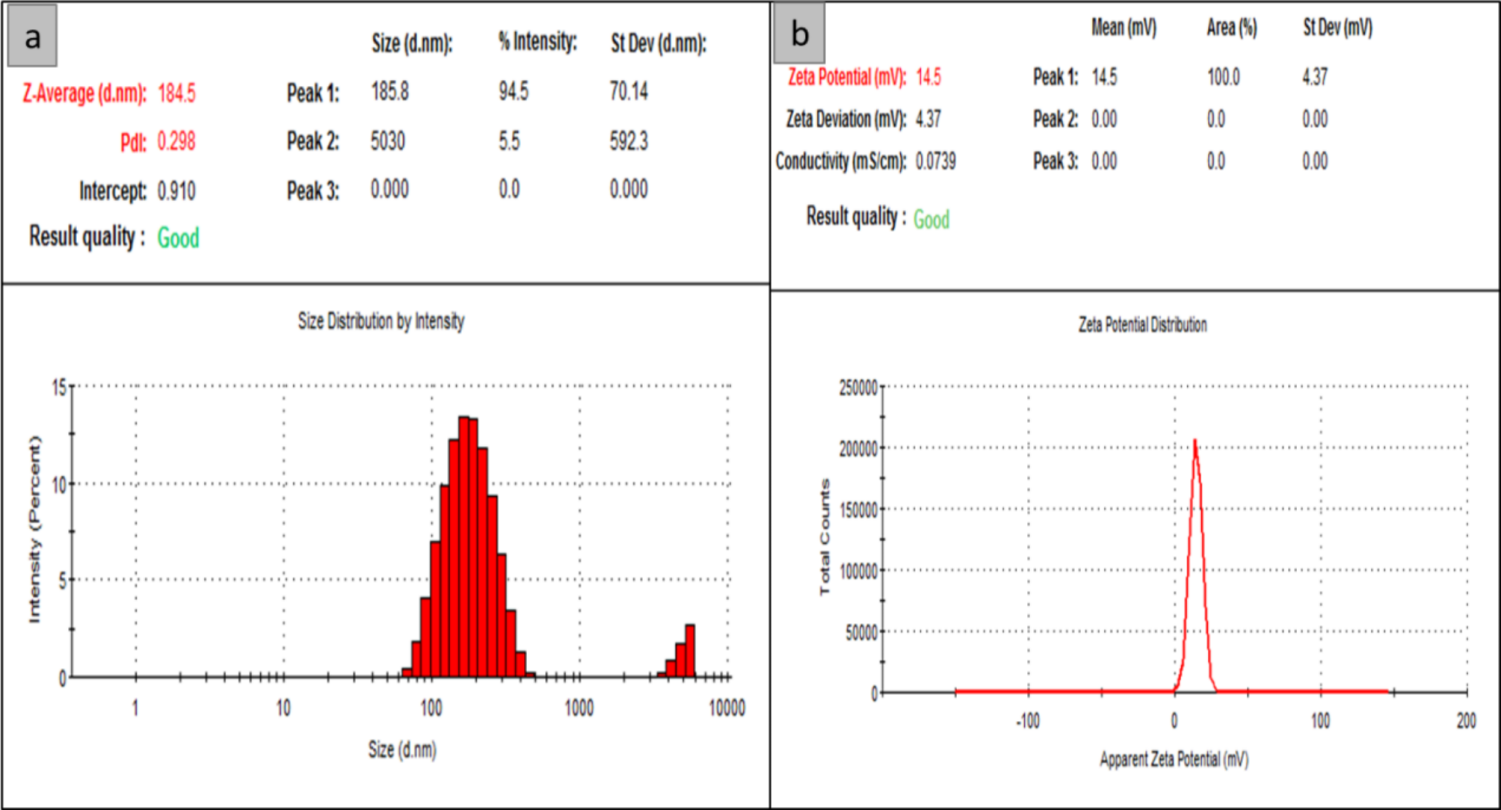
DLS and Zeta potential of the synthesized  **8b**-NLC-Cs nanoformulation.

The stability profile of nanoparticles is restricted to zeta potential (z.p), where the most stable nanoformulations’ z.p values are > (+ 30) and < (− 30). By the mean, stable nanoformulations have z.p values greater than + 30 and smaller than (− 30). Z.P of the free NLC-Cs and **8b**-NLC-Cs (with drug) were (+ 15.1mV) and (+ 14.5mV), respectively. The positive charge in the two formulations, as shown in Fig. 4, is due to the incorporation of chitosan and its free amino group in an acidic medium^63^. Total z.p charge conversion from negative charges for NLCs formulations^64^ to the positive values (for chitosan-NLC formulations), could confirm the deposition (coating) of chitosan layer on the surface of the NLC nanoparticles. As a result of high-density amino groups comes from the accumulation of chitosan, leading to increasing the density of positive charge clouds that affected the net particle charge. This shift, from negative to positive charges, could be attributed to the hydrogen bonding between chitosan as a polysaccharide (amino groups) and the lipid (glyceride head groups). That complex formation could give convenient justification for the coating mechanism of the concerned NLC-Cs nanoformulation. Similar studies focused on lipid carriers coated with a chitosan layer agreed with these results^65–67^. Pan^68,69^ reported the stability of the free NLC and its coated form (NLC-Cs) by following the measurements of z.p before and after coating, similar results were obtained and z.p converted from negative to positive charge as a net result of chitosan coating. As previously reported^70,71^, nanoparticle surface charge has a remarkable impact on the drug pharmacokinetic parameters, especially the positively charged nanoparticles that could interact with the negatively charged cell membrane components^72^. Furthermore, the small particle size will effectively improve bacteria cell internalization *via* endocytosis mechanism^73^.

#### NLC surface morphology by transmission electron microscope (TEM)

**Fig. 5:**
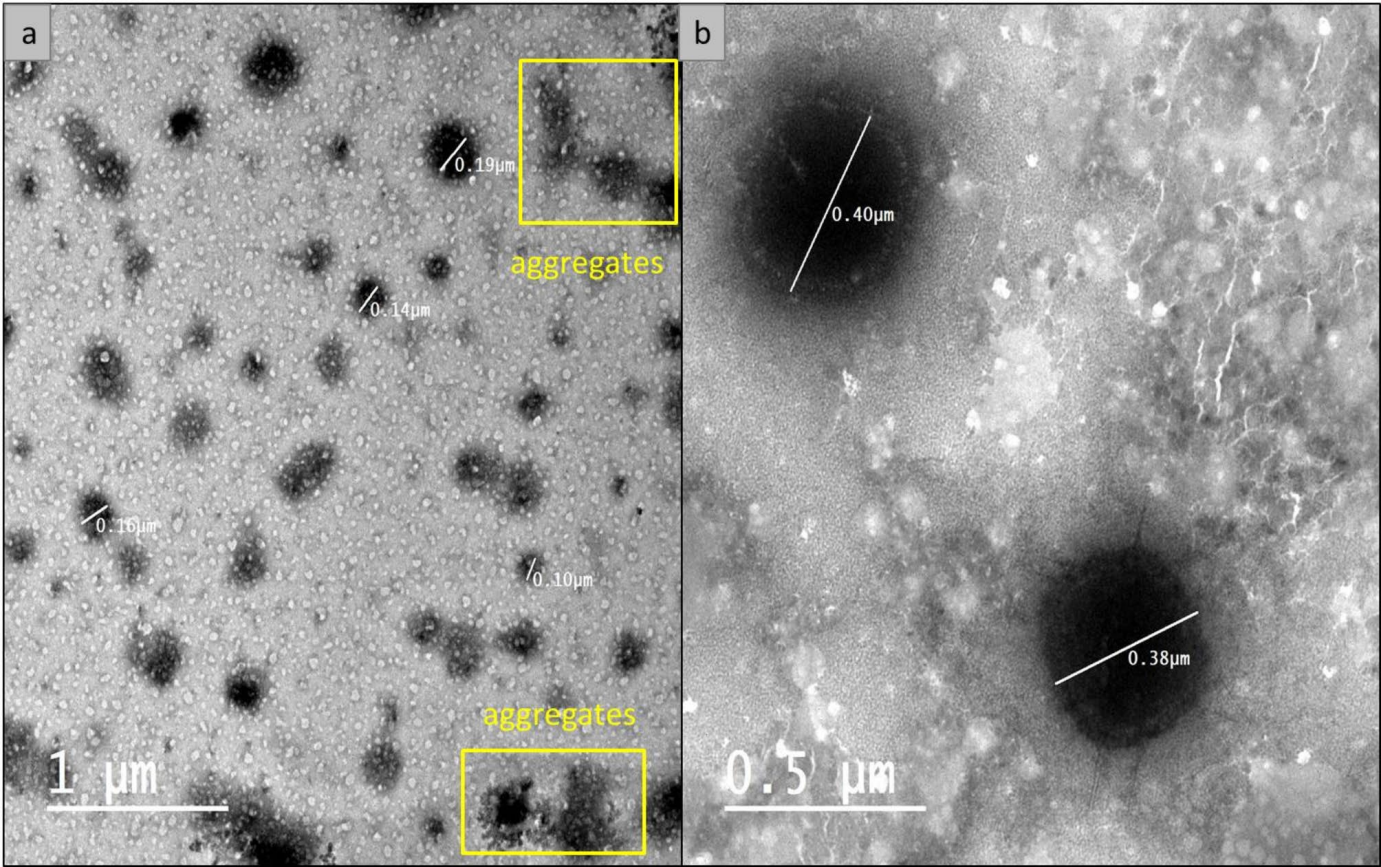
TEM micrograph of the synthesized coumarin derivatives encapsulated lipid-chitosan nanocapsule (**8b**-NLC-Cs): (**a**) TEM-field describes varied sizes distribution with little aggregations, (**b**) TEM-field describes two spherical and size-comparable nanoparticles.

A High-resolution transmission electron microscope (HR-TEM) is one of the most efficient ways to analyze the internal particle’s structure and size distribution of the nanoparticles. Non-encapsulated NLC-CS and capsulated **8b**-NLC-Cs showed morphological characteristics like those previously reported^74^. The morphology elucidation of the prepared NLC nanoparticles is described with regular, spherical and semi spherical particles with certain aggregates as described in Fig. 5a with size ranges of 100–200 nm, and 300–400 nm for NLC-Cs (without drug) and **8b**-NLC-Cs, respectively. Previous studies focused on the synthesis of NLC encapsulated with different active ingredients were confirmed the particle size in rage of 200–500 nm and sometimes exceed one micrometer^75–77^. The HR-TEM micrograph confirmed the same results obtained from DLS and PDI. The largeness in particle size measured by HR-TEM is not confusing with the particle size measured by DLS as its protocol is based on imaging a specific field contain few particles, nevertheless, DLS assessed on measuring average size, not specific particle size. Figure 5b shows the encapsulation of **8b**-NLC-Cs in the NLC’s vicinity as the inner core and the outer layer represented the lipid np coated with the chitosan layer. TEM imaging besides the positively charged z.p results could represent a convenient proof of chitosan coating.

#### Fourier transform infra-red (FTIR)

**Fig. 6:**
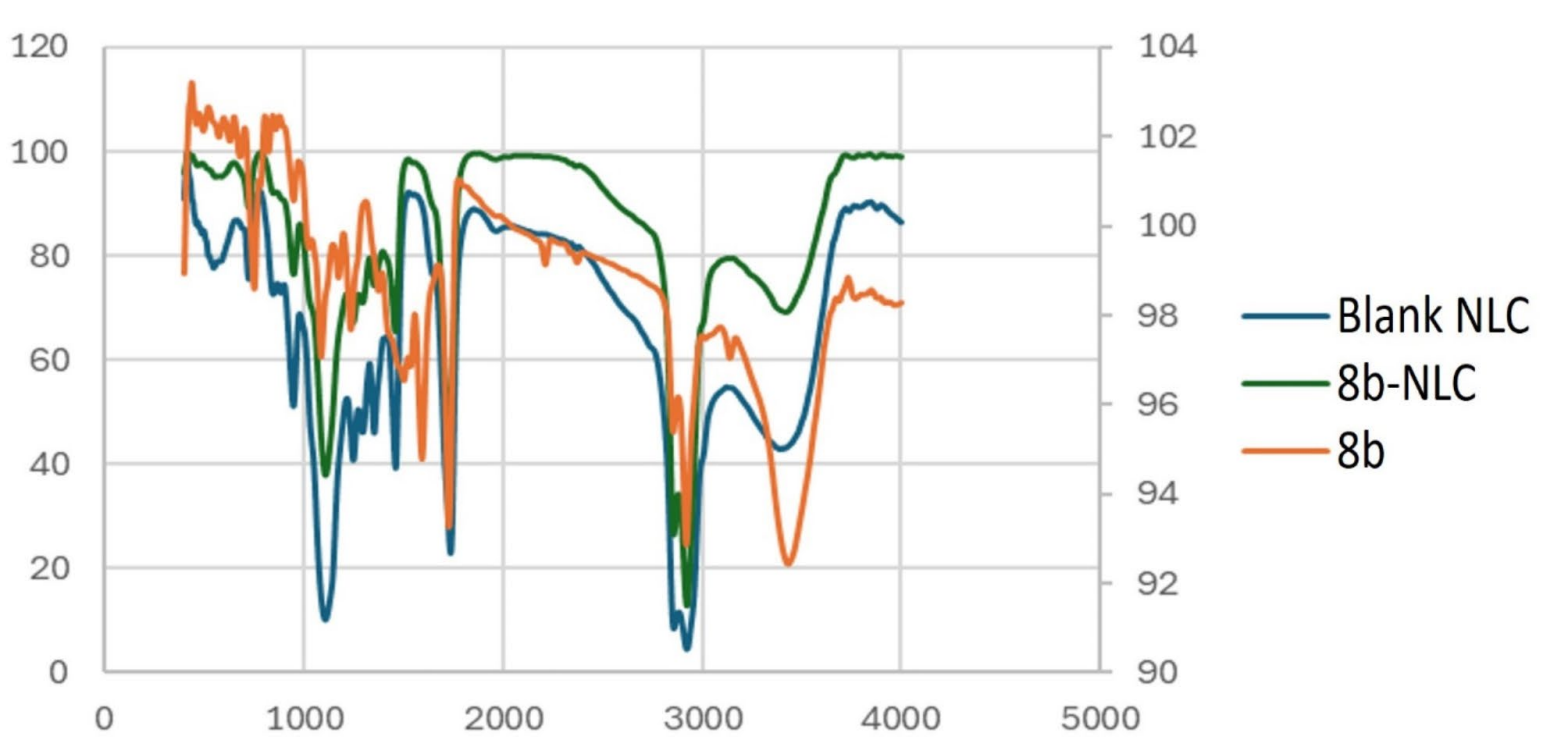
Fourier transform infrared (FTIR) of blank NLC, **8b** and **8b**-NLC-Cs.

The interaction manifestation between drug **8b**, and nanostructure lipid carrier was done using Fourier transform infra-red. The synthetic compound **8b** revealed remarkable sharp peaks at wave number of λ^−1^ = 2212, and 1727 cm^−1^ corresponding to the cyano group (C ≡ N) and carbonyl group (C = O), respectively as shown in Fig. 6. On the other hand, absence of such peaks supports the encapsulation of the compound **8b** to the vicinity of the NLC structure.

#### Drug loading capacity (DL) and entrapment efficiency (EE)

**Fig. 7:**
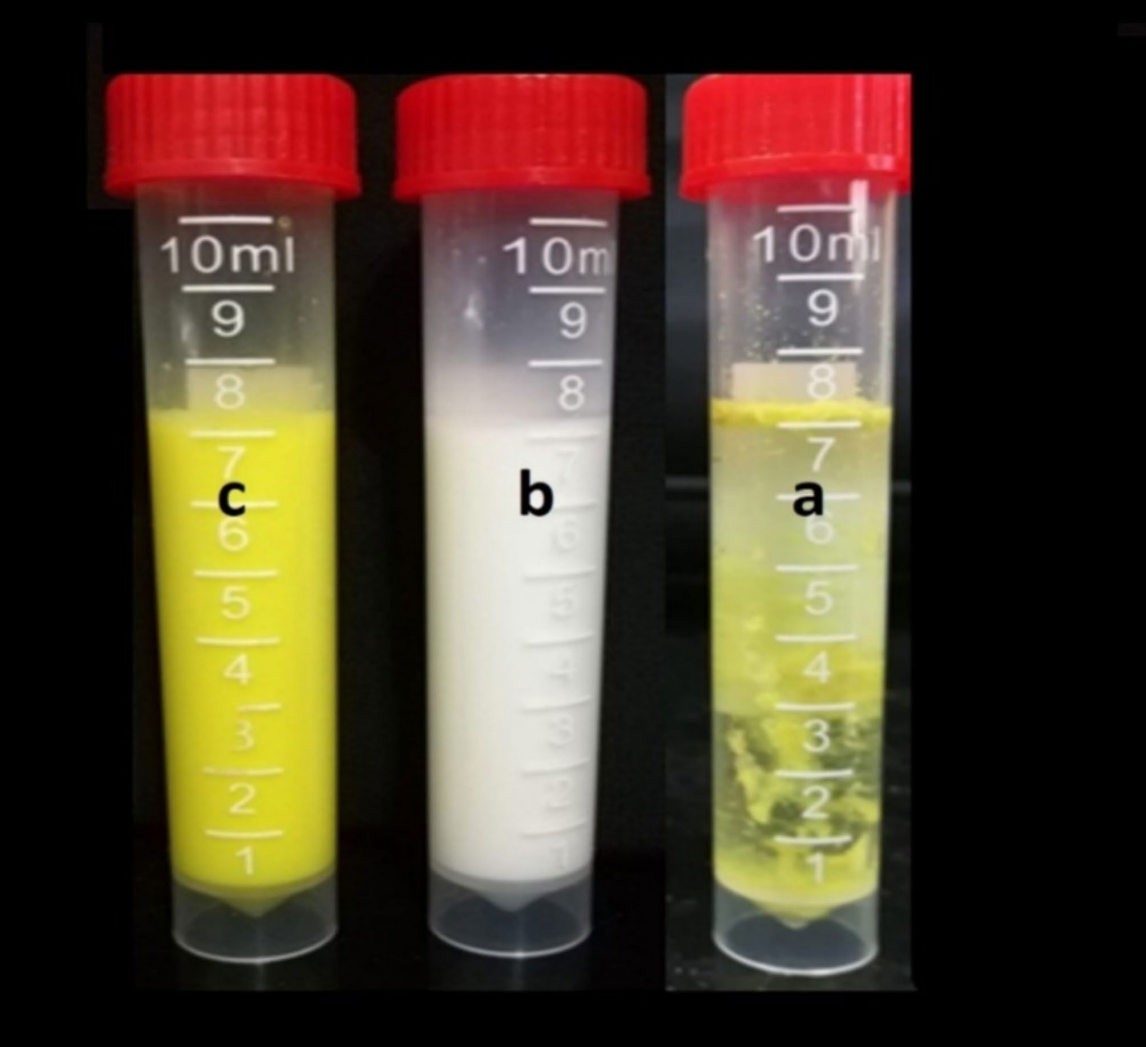
The solubility and dispersibility of the target drug and its conjugate nanoformulations in distilled water: (**a**) represented the in-solubility of the hydrophobic drug (**8b**) in water, (**b**) well-dispersed free nanostructure lipid carrier without drug (NLC-Cs) in water, (**c**) well-dispersed encapsulated nanostructure lipid carrier with the target drug (**8b**-NLC-Cs) in water.

The Encapsulation Efficiency percentage (%) and drug loading capacity of coumarin derivatives **8b** were determined after the separation of the unloaded **8b** from the NLC-Cs nanoformulation using ultrafiltration centrifugation. The entrapment efficiency (EE) or the percent of drug content successfully encapsulated or entrapped into the nanoparticles was calculated as the ratio between the actual quantities of drug entrapped divided by the total drug added. Whereas, the DL was defined as, the amount of drug loaded per unit weight of the nanoparticle, indicating the percentage of mass of nanoparticle that is due to the encapsulated drug and calculated by, the amount of total entrapped drug divided by the total nanoparticle weight. The EE and DL were found to be  91%, and 68.25%, respectively. Therefore, most of the drug (27.3 mg), from the original amount of 30 mg, was entrapped effectively in the NLC-Cs system. The elevated loading efficiencies might be attributable to the hydrophobic nature and significant insolubility in water (Fig.7)  of the coumarin derivative **8b**, which promotes the incorporation into the vicinity of the liquid lipid core^78^. Consequently, delayed leakage of **8b** from the lipid core of the NLC-Cs nanoparticles to the surroundings through centrifugation^79^ that may raise convenient interpretation of the higher entrapment efficiency.

#### Biological section

**Fig. 8:**
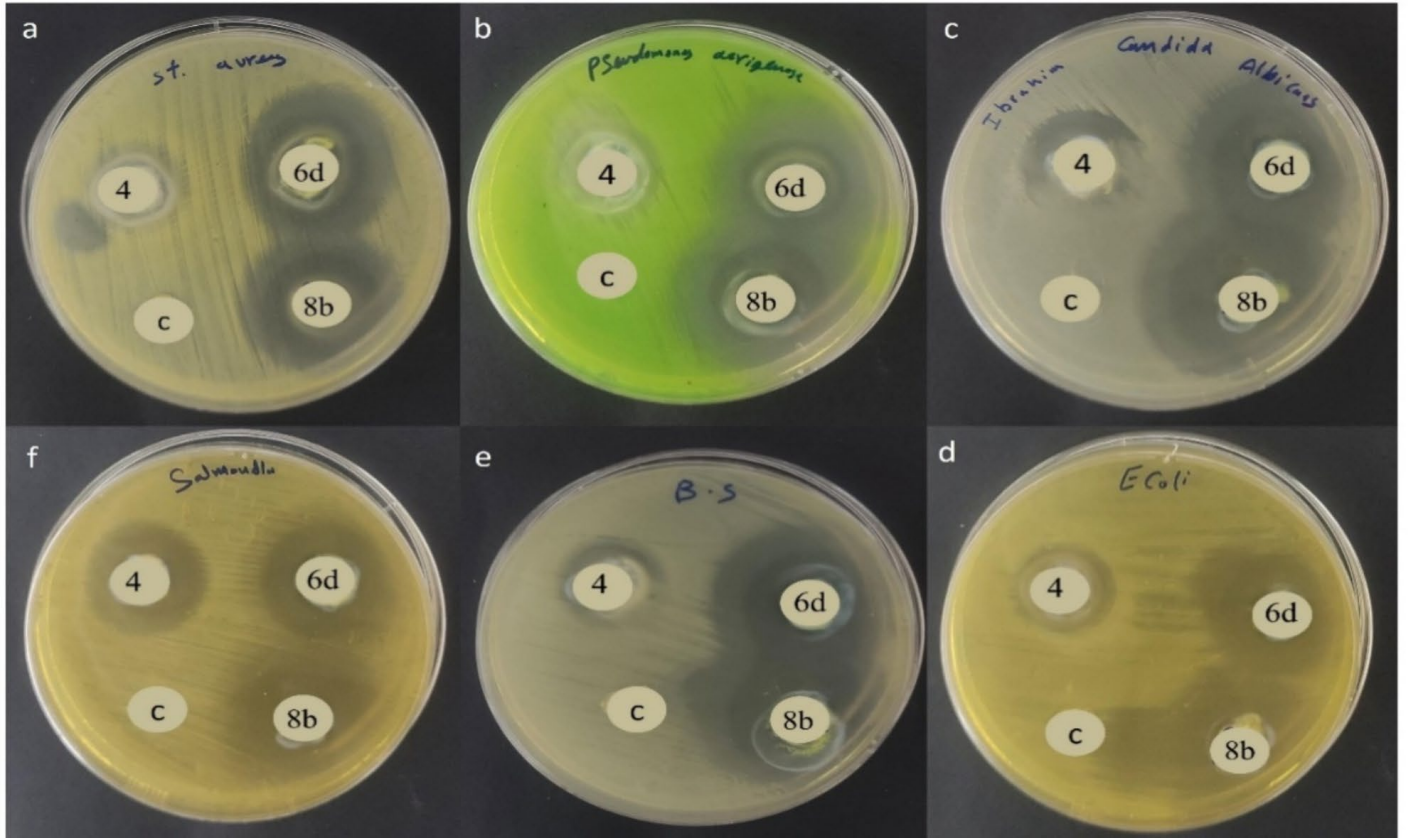
Antimicrobial evaluation of the coumarin derivatives after nanoformulation: (**a**) *S. aureus*, (**b**) *P. aeruginosa*, (**c**) *C. albicans*, (**d**) *E. coli*, (**e**) *B. subtilis*, (**f**) *S. typhi*.

Antimicrobial evaluation were assessed through determining the inhibition zone diameter and minimal inhibitory concentration for all the synthesized compounds of **4**, **6a-d**, **8a-c**, **9**, **11a**,** b**, **15a**,** b**, and **18a-d** against six pathogens of *B. subtilis*, *S. aureus*, *E. coli*, *P. aeruginosa*, *S. typhi*, *and C. albicans*. The screening results displayed in Table 1 manifested that all tested materials had broad activity (wider i.z) against all microbes except *S. typhi*. The bacterial susceptibility of *S. aureus* and *C. albicans* substantially affected by compound **8b** (26, and 30 mm, respectively). Due to their high antimicrobial susceptibility to *B. subtilis*, *S. aureus*, *E. coli*,* C. albicans* and *P. aeruginosa*. Compounds **4**, **6d**, and **8b** were chosen to be nano-encapsulated to the vicinity of lipid-chitosan nanocapsule (NLC-Cs). Coumarin-based NLC-Cs nanoformulations, were tested towards the same pathogens and inhibition zones in addition; minimum inhibitory concentration were performed (Tables 2 and 3) After nano encapsulation, significant changes in the inhibition zone diameter have been made. Compound **6d** accomplished i.z of 23, 27, 20, 21, NA and 22 mm before nano while the i.z after nano increased to 34, 28, 30, 31, 26, and 35 mm. Similarly, **8b**-NLC-Cs nanoformulation displayed unique antimicrobial activity with i.z of 40, 25, 28, 33, 29 and 40 mm, while **8b** before nanoformulation were 22, 24, 17, 18, NA and 30 mm, against *B. subtilis*, *S. aureus*, *E. coli*, *P. aeruginosa*, *S. typhi*, and *C. albicans*, respectively (Fig. 8). It is noteworthy that all the tested drugs after nano encapsulation gained powerful inhibitory effect against *S. typhi* bacteria after they had no activity before. Minimal inhibitory concentration represents the lowest concentration that is required to inhibit bacterial growth. The conjugate **4-NLC-Cs**, **6d-NLC-Cs** and **8b-NLC-Cs** nps demonstrated prospective suppression in the MIC values as example **6d**-NLC-Cs reduced from 62.5 to 0.48 µg/ml (130 folds) while **8b**-NLC-Cs minimized from 15.6 to 0.24 µg/ml (65 folds) against *S. aureus*. Perhaps the distinctive thing about the structural formula of such compounds is not only that they possess coumarin moieties, but also that they contain thiazole and pyrazole, which work together to mostly enhance the biological activity^80–82^. Similar results were concluded by Vedula and co-workers^83^ who reported the microwave irradiation one-pot synthesis of a new series of hybrid coumarin based thiazole, after antimicrobial evaluation against a gram-positive spheroid firmicute, potential MIC results were obtained (less than 5 µg/ml). Synergistic antimicrobial effect has declared in the one-pot, three components synthesis of thioether liked 4-hydroxy coumarin and promising anti-tuberculosis results were concluded^84^. Recent work frames based on coumarin nucleus not only proved their unique antimicrobial, but their work took their steps to be in the global market and they have taken approval from the FDA as antibiotics like aminocoumarin, novobiocin and clorobiocin^85^. The chitosan in the outer layer capsule investigated as an antimicrobial material against a wide range of different types of microorganisms such as gram-positive and gram-negative bacteria^86,87^. The inhibition model of chitosan has been proposed, the most acceptable being the interactions between positively charged chitosan molecules and negatively charged microbial cell membrane. In this model the interaction is mediated by the electrostatic forces between NH_3_^+^ and the cell membrane residues^88^ causing severe damage in the cell wall and as a results cell rupture^89^. The designed coumarins which contains more than active species like coumarin itself, thiazole, naphthalene and/or pyrazoline groups in addition to chitosan as an outer shell layer nanocarrier, make the microbial strains more sustainable. Cumulatively, synergistic effects were generated, that could inhibit microbes effectively with promising MIC values.

**Table 1:** Antimicrobial screening (inhibition zone diameter) of the synthetic coumarin derivatives.

Compd. no.	Types of pathogens with inhibition zone diameter of tested coumarins (mm)					
	B. Subtilis	S. aureus	E. coli	*P*. aeruginosa	S. typhi	C. albicans
4	24 ± 0.76	28 ± 0.57	22 ± 1.15	19 ± 0.57	NA	76 ± 0.76
**6a**	19 ± 0.28	NA	NA	NA	NA	16 ± 0.00
**6b**	21 ± 0.50	25 ± 0.76	20 ± 0.50	20 ± 0.00	NA	0.57 ± 27
**6c**	23 ± 0.28	24 ± 0.28	16 ± 0.00	22 ± 0.50	NA	28 ± 0.76
**6d**	**23 ± 0.28**	**27 ± 0.57**	**20 ± 0.76**	**21 ± 0.28**	**NA**	**22 ± 0.57**
**8a**	20 ± 0.28	24 ± 0.57	19 ± 0.00	18 ± 0.00	NA	25 ± 0.50
**8b**	**24 ± 0.76**	**26 ± 0.57**	**18 ± 0.00**	**19 ± 0.50**	**NA**	**30 ± 0.57**
**8c**	21 ± 0.28	25 ± 0.57	18 ± 0.00	17 ± 0.28	NA	27 ± 0.76
**9**	18 ± 0.00	NA	NA	NA	NA	20 ± 0.28
**11a**	17 ± 0.50	NA	NA	NA	NA	NA
**11b**	18 ± 0.28	NA	16 ± 0.57	NA	NA	16 ± 0.76
**15a**	22 ± 0.00	NA	NA	NA	NA	14 ± 0.28
**15b**	19 ± 0.57	NA	NA	NA	NA	NA
**18a**	19 ± 0.76	22 ± 0.57	22 ± 0.57	25 ± 0.76	NA	24 ± 0.28
**18b**	21 ± 0.57	23 ± 0.76	19 ± 0.28	20 ± 0.00	NA	26 ± 0.57
**18c**	25 ± 0.50	21 ± 0.00	16 ± 0.28	23 ± 0.76	NA	22. ± 0.00
**18d**	22 ± 0.28	25 ± 0.76	20 ± 0.00	20 ± 0.57	NA	20 ± 0.00
**Gentamicin**	22 ± 0.28	15 ± 0.00	17 ± 0.28	16 ± 0.00	23 ± 0.57	20 ± 0.50

Significant values are given in bold.

**Table 2:** Minimal inhibitory concentration (MIC) and number of activity folds of the most active coumarin compounds before and after nano-encapsulation.

Pathogen	Comp 4 MIC (µg/ml)		No. folds	Comp 6d MIC (µg/ml)		No folds	Comp 8b MIC (µg/ml)		No folds
	Before	After nano		Before	After nano		Before	After nano	
*B. subtilis*	31.25	15.6	2	31.25	1.95	16	15.6	0.48	32.5
*S. aureus*	62.5	62.5	1	62.5	0.48	130	15.6	0.24	65
*E. coli*	15.6	7.8	2	15.6	15.6	1	15.6	0.97	16
*P. aeruginosa*	31.25	7.8	4	31.25	7.8	4	15.6	0.48	32.5
*S. typhi*	**125**	**62.5**	**2**	62.5	15.6	4	**250**	**31.25**	8
*C. albicans*	62.5	62.5	1	62.5	1.95	32	31.25	0.48	65

Significant values are given in bold.

#### Suggestion of the anti-bacterial mode of action

The mode of action of coumarin-NLC-Cs was studied by tracking the morphological changes of the *P. aeruginosa* before and after treatment using transmission electron microscope. The **8b-**NLC-Cs nps was tested for its DNA gyrase inhibition using novobiocin, coumarin-based antibacterial, and DNA gyrase inhibitor, as a reference control.

#### I-TEM examination of bacteria

**Fig. 9:**
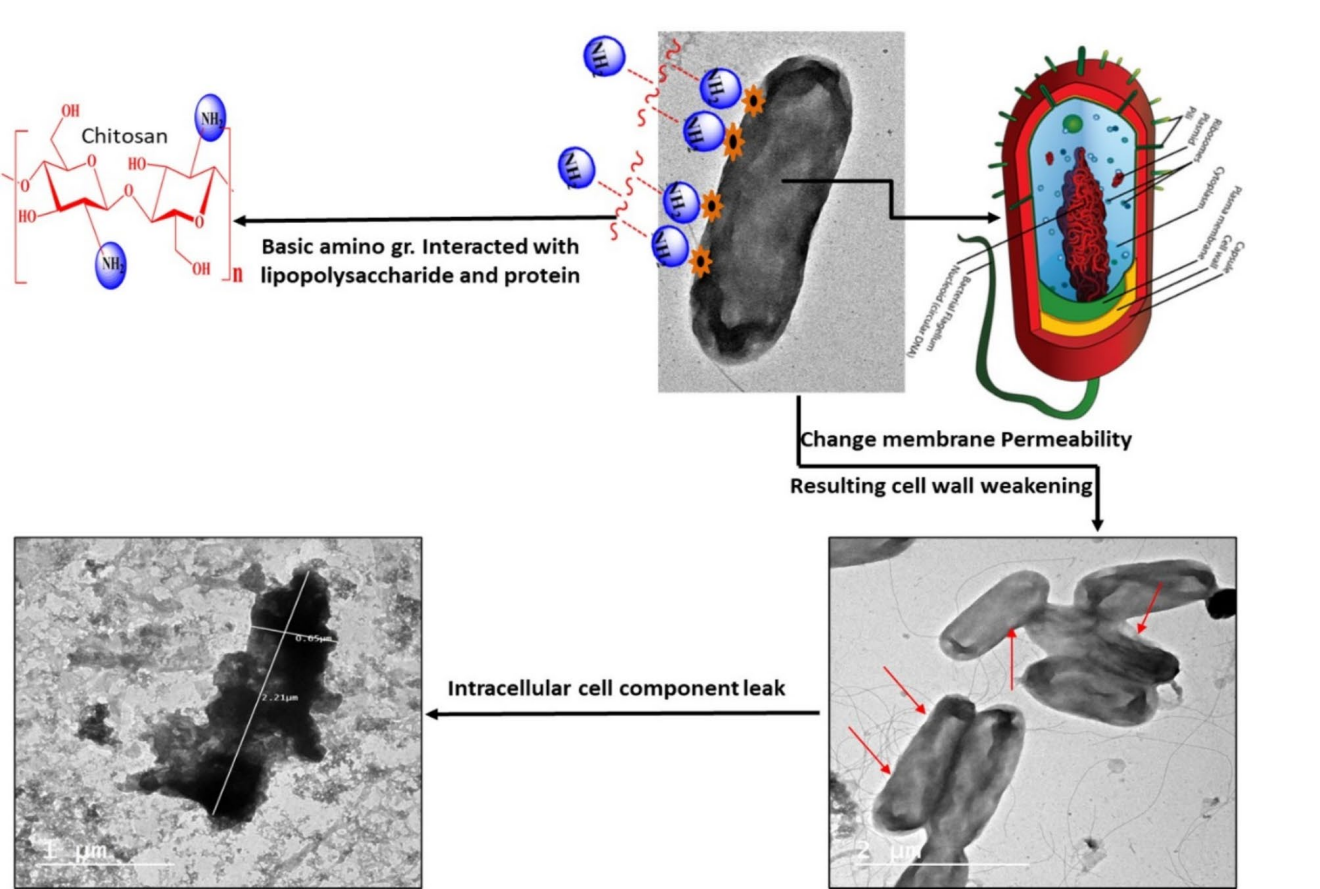
The role of the combination between coumarin and chitosan in interpreting the anti-bacterial mode of the *P. aeruginosa* strain after treatment with **8b**-NLC-Cs nanoformulation (the 3D bacteria model uploaded from: https://breannarachelgorry.weebly.com/what-does-p-aeruginosa-bacteria-looklike.html).

**Fig. 10:**
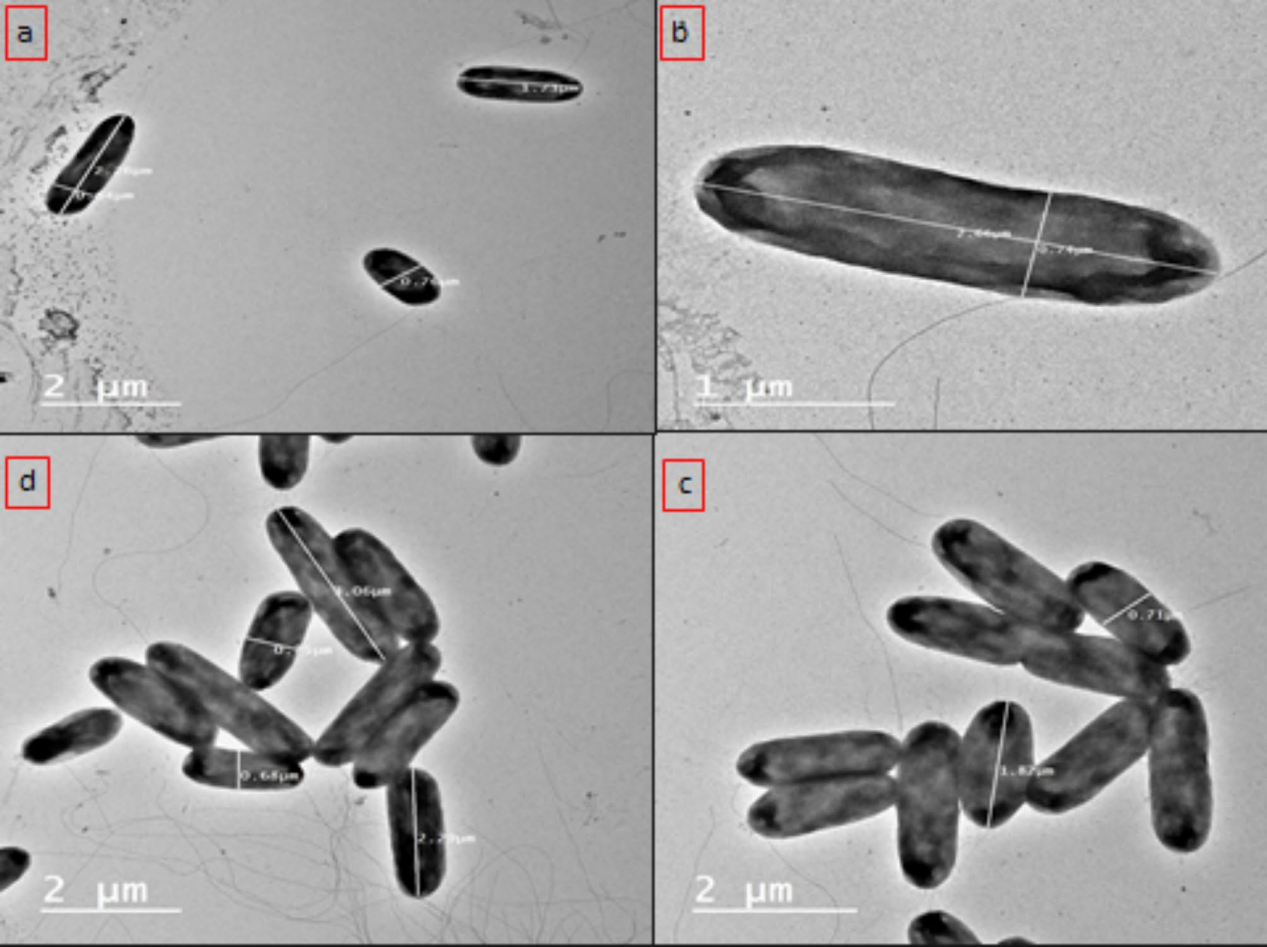
(**a**–**d**) Represent a transmission electron microscope (TEM) examination of untreated *P. aeruginosa* with normal sizes and dimensions.

**Fig. 11:**
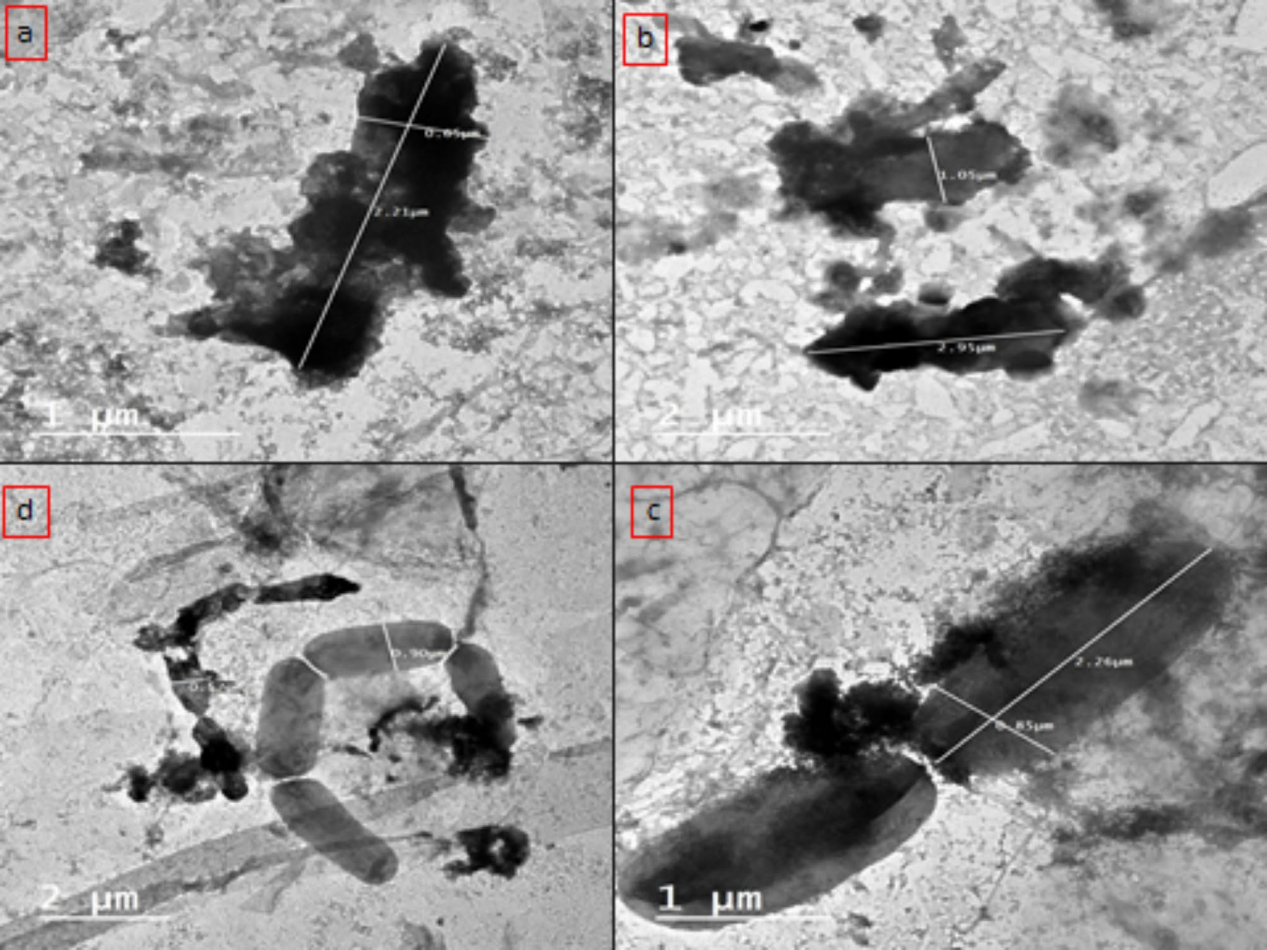
Transmission electron microscope (TEM) examination of the treated *P. aeruginosa* by **8b**-NLC-Cs nanoformulation at different stages: (**a**,**b**) complete inhibition of *P. aeruginosa*, (**c**) cell wall permeability changes and lysis starting up, (**d**) two types of treated cells of were found (cells with ruptured cell wall and cells with lysis starting up).

The structure of  **8b**-NLC-Cs basically contains three main components, the biologically active **8b** presented in the vicinity of NLC which mainly consists of different liquid and solid lipids (second component), and all of that was surrounded by chitosan layer, as proven before by TEM and z.p studies. When the conjugate **8b** In the beginning, before any drug release, 8b-NLC-Cs started to contact the bacterial cells with the chitosan layer (Fig. 9). Consequently, the chitosan terminated amino groups reacted with the negatively charged bacteria cell surface. Attraction forces generated because of polycationic chitosan with lipopolysaccharides and proteins found in the bacterial cell wall proposed to change the chemistry of bacteria surface, changing in the permeability barrier, leakage inside cells (lysis) and causing membrane rupturing and intracellular components leaking out the cell with preventing the essential nutrients from entering the cell^90^. Moreover, in consistent with the results of the study herein, some researchers reported complete cell membrane damage with higher concentrations of chitosan^91^. As referred in Figs. 10 and 11, which presented the bacteria cells of *P. aeruginosa* before and after treatment with the **8b**-NLC-Cs nanoformulation, the bacteria cells affected potentially causing severe cell wall damage.

#### II-DNA gyrase inhibition

**Table 3:** DNA gyrase enzyme activity assay.

Sample	Conc.	Log conc.	Inhibition %	M. wt (g/mol)	IC_50_ ± SD (µg/ml)	IC_50_ (µM) ≡ (µmol/L)
4	100	2	72	268.29	9.67 ± 0.57	36.04 ± 1.11
	10	1	57			
	1	0	21			
	0.1	−1	2.8			
EC (control)	–	–	0			
6d	100	2	73	407.23	12.1 ± 0.71	29.71 ± 1.74
	10	1	51			
	1	0	17			
	0.1	−1	0.5			
EC (control)	–	–	0			
8b	100	2	78	512.29	2.34 ± 0.14	4.56 ± 0.52
	10	1	62			
	1	0	42			
	0.1	−1	27			
EC (control)	–	–	0			
Doxo	100	2	81	543.52	1.57 ± 0.09	2.88 ± 0.16
	10	1	71			
	1	0	51			
	0.1	−1	22			
EC (control)	–	–	0			

In bacteria, the negative supercoil of DNA is generated and maintained by DNA gyrase, a type IIA topoisomerase, which is mainly involved in DNA replication and transcription. Gyrase introduces a negative supercoil into DNA at the cost of hydrolyzing ATP, and this negative supercoil generates reversible transient double-strand beaks, thereby relieving supercoil from the external forces acting on DNA during cell growth, DNA replication, and transcription, and other intracellular processes DNA (over-wound DNA) is relaxed .

Increased negative supercoiling in front of the moving polymerase and relaxation behind it is considered essential for DNA replication and gene transcription by both the transcription machinery. The inhibitory activity of the synthesized compounds against DNA gyrase enzyme was evaluated in (Table 3). The results showed that compound **8b** revealed good inhibitory activity with a reduced IC_50_ value of 4.56 µM, while the positive control revealed had an IC_50_ value of 2.88 µM. In a similar study, novel *N*-phenyl pyrrolamide derivatives were synthesized and evaluated for their ability to inhibit DNA gyrase and topoisomerase IV from bacterial sources (*S*,* aureus and E. coli*) with reduced IC_50_ of 47 nM^92^.

## Experimental

### Materials and methods

#### Materials

2-hydroxy benzaldehyde, ethyl acetoacetate, methanol, piperidine, bromine water, acetic acid, iron sulfide, malononitrile, hydrochloric acid, absolute ethanol, 3,4-dimethoxybenzaldehyde, *N*,* N*-dimethoxyaminobenzaldehyde, indole-3-carboxaldehyde, 2-naphthaldehyde, 3-hydroxy benzaldehyde, vanillin, 4-hydroxybenzaldehyde, 2,4-dihydroxybenazldehyde, hydrochloric acid, sodium hydroxide, sodium carbonate, *p*-toluene sulfonic acid (*P*TSA), phosphorus oxychloride, acetophenone, 4-methylacetophenone, 4-chloroacetophenone, aniline, 4-chloroaniline, 4-methylaniline, 4-methoxyaniline and phenyl hydrazine, dimethyl sulfoxide (DMSO), stearic acid, oleic acid, Tween 20 and deionized water were purchased from Alfa Aesar, Germany. Whereas the chitosan low M.W and pluronic F127 were purchased from Merck (Sigma Aldrich), Germany. Solvents like commercial ethanol, methanol, dimethylformamide, and glacial acetic acid were purchased from a local supplier, El Gomhoria Company, Cairo Egypt. All chemicals and solvents are used without purification. All measured melting points are uncorrected and were tested in our Lab using electro-thermal capillary melting point apparatus (6100).

FTIR spectra were recorded on a Perkin Elmer 1430 spectrophotometer. Mass spectra were examined by Shimaduze GMCS-GB1000 EX mass spectrophotometer at 70ev at Micro analytical Center Laboratory, Cairo University. Elemental analysis was carried out at the Microanalyses Center of Cairo University, Giza, Egypt, and the results are within ± 0.4% of the theoretical values.

The results of^1^H NMR and^13^C NMR spectroscopy in deuterated dimethyl sulfoxide at mercury 400 and 100 MHz on a Varian Gemini NMR spectrophotometer using tetramethylsilane as internal reference are predicted as δ value, Faculty of Pharmacy, Cairo University, Giza, Egypt.

The starting materials 3-acetyl-2*H*-chromen-2-one and 3-(2-bromoacetyl)-2*H*-chromen-2-one were prepared according to the previous reports^93,94^.

##### Synthesis of 2-(4-(2-oxo-2 H-chromen-3-yl) thiazol-2-yl) acetonitrile 4

A mixture of 2-cyanothioacetamide **3** (0.01 mol, 1 g) and 3-(2-bromoacetyl)-2*H*-chromen-2-one **2** (0.01 mol, 2.67 g) were stirred under reflux conditions in absolute ethanol (30 ml) for 2 h. The precipitate so formed was collected and filtered off then recrystallized from ethanol.

Off white crystals; yield 77%; m.p. 188–190 °C; IR (KBr, cm^−1^), 2253 (CN), 1739 (CO);^1^H NMR (DMSO*d*_*6*_):δ = 4.64 (s, 2 H, CH_2_), 7.39–7.46 (m, 2 H, Ar), 7.62–7.67 (m, 1 H, Ar), 7.92–7.95 (m, 1 H, Ar), 8.74 (s, 1 H, coumarin-H), 8.75 (s, 1 H, thiazole-H); Anal. Calcd for C_14_H_8_N_2_O_2_S (268.29): C, 62.68; H, 3.01; N, 10.44; S, 11.95. Found: C, 62.61; H, 2.89; N, 10.12; S, 11.85%.

##### General procedure for synthesis of compounds 6a-d

Method A:

2.68 g (0.01 mol) of 2-(4-(2-oxo-2*H*-chromen-3-yl)thiazol-2-yl)acetonitrile **4** was added to 0.01 mol of aromatic aldehydes in 20 ml absolute ethanol in a round bottom flask with reflux for 3 h with the addition of 4–5 drops of piperidine. After the reaction completion, the flask attained to cool, to form yellow precipitate, then filtered off and washed several times with ethanol and recrystallized from 1,4-dioxane.

Method B:

2.68 g (0.01 mol) of 2-(4-(2-oxo-2*H*-chromen-3-yl) thiazol-2-yl) acetonitrile **4** was added to aromatic aldehydes **5a-d** (0.01 mol) with addition of 0.005 gram of *P*-TSA. The mixture was grinding at room temperature for 20 min. After the reaction completion, water was added, and the precipitate was filtered off and washed several times with ethanol, and recrystallized from a mixture of EtOH and DMF.

**(3-(3**,**4-Dimethoxyphenyl)-2-(4-(2-oxo-2*H*-chromen-3-yl)thiazol-2-yl)acrylonitrile 6a**:

Yellow crystals; yield; 80% (A), 88% (B); m.p. 280–282 °C; IR (KBr, cm^−1^): 2233 (CN), 1730 (CO);^1^H NMR (DMSO*d*_*6*_): δ = 3.83 (s, 3 H, OCH_3_), 3.86 (s, 3 H, OCH_3_) 7.41 (d, 1 H, *J* = 8.4 Hz, Ar), 8.21 (m, 6 H, CH and Ar), 8.54 (d, 1 H, *J* = 8.4 Hz, Ar), 8.77 (s, 1 H, coumarin-H), 8.42 (s, 1 H, thiazole-H); Anal. Calcd for C_23_H_16_N_2_O_4_S (416.45): C, 66.33; H, 3.87; N, 6.73; S, 7.70. Found: C, 66.14; H, 3.77; N, 6.62; S, 7.69%.

**3-(4-(*N***,*** N*-Dimethylamino)phenyl)-2-(4-(2-oxo-2*H*-chromen-3-yl)thiazol-2-yl)acrylo-nitrile 6b**:

Yellow crystals; yield; 78% (A); 80% (B); m.p. 288–290 °C; IR (KBr, cm^−1^): 2215 (CN), 1735 (CO);^1^H NMR (DMSO*d*_*6*_): δ = 3.40 (s, 3 H, CH_3_), 3.43 (s, 3 H, CH_3_), 7.94 (d, 2 H, *J* = 9 Hz, Ar), 8.78 (s, 1 H, coumarin-H), 8.43 (m, 7 H, CH and Ar) 8.71 (s, 1 H, thiazole-H); m/z = 399.19 (M^+^,54.1%), 384.33 (14.6%), 229.29 (12.1%), 299.60 (19.3%), 174.18 (11.1%), 140.10 (14.6%), 127.21 (20%), 115.24 (22%), 102.22 (100%); Anal. Calcd for C_23_H_17_N_3_O_2_S (399.10): C, 69.15; H, 4.29; N, 10.52; S, 8.03. Found: C, 69.33; H, 4.12; N, 10.29; S, 8.21%.

**3-(1*H*-Indol-3-yl)-2-(4-(2-oxo-2*H*-chromen-3-yl) thiazol-2-yl)acrylonitrile 6c**:

Pale yellow crystals; yield; 70% (A); 76% (B); m.p. 286–288 °C; IR (KBr, cm^−1^): 3240 (NH), 2214 (CN), 1707 (CO);^1^H NMR (DMSO*d*_*6*_): δ = 7.28–7.31 (m, 3 H, Ar), 7.42–7.51 (m, 2 H, Ar), 7.57–7.70 (m, 2 H, Ar), 7.94–8.01 (m, 2 H, Ar), 8.12–8.14 (m, 1 H, Ar), 8.46 (s, 1 H, coumarin-H), 8.53 (s, 1 H, CH), 8.64 (s, 1 H, Ar), 8.98 (s, 1 H, thiazole-H), 12.38 (s, 1 H, NH);^13^C NMR (DMSO): 110.8, 113.2, 116.4, 119.6, 120.1, 121.9, 123.8, 125.3, 129.6, 130.5, 132.7, 136.7, 138.6, 140.5, 152.9, 159.1;m/z = 395.25 (M^+^, 18.3%), 361.21 (16.2%), 272.23 (16.90%), 263.23 (49.8%), 171.22 (26.8%), 145.18 (42.8%), 102.19 (100%), 73.26 (33.4%); Anal. Calcd for C_23_H_13_N_3_O_2_S (395.07): C, 69.86; H, 3.31; N, 10.63; S, 8.11. Found: C, 69.70; H, 3.49; N, 10.87; S, 8.30%.

**3-(Naphthalen-2-yl)-2-(4-(2-oxo-2*H*-chromen-3-yl) thiazol-2-yl) acrylonitrile 6d**:

Brownish-yellow crystals; yield; 72% (A); 78% (B); m.p. 288–290 °C; IR (KBr, cm^−1^): 2216 (CN), 1717 (CO);^1^H NMR (DMSO*d*_*6*_): δ = 7.14–7.49 (m, 2 H, Ar), 7.29–7.70 (m, 3 H, Ar), 7.94–8.12 (m, 9 H, CH and Ar), m/z = 406.23 (M^+^, 70%), 350.23 (12.2%), 289.14 (41.9%), 274.28 (16.8%), 249.28 (10.8%), 171.22 (22.7%), 145.22 (32.8%), 102.22 (100%); Anal. Calcd for C_25_H_14_N_2_O_2_S (406.08): C, 73.87; H, 3.47; N, 6.89; S, 7.89. Found: C, 73.70; H, 3.96; N, 6.63; S, 7.70%.

##### The general procedure for the synthesis of compounds 8a-c

A mixture of 2-(4-(2-oxo-2*H*-chromen-3-yl)thiazol-2-yl) acetonitrile 4 (0.01 mol) was added to pyrazole-4-carbaldehydes **7a-c** (0.01 mol) in 20 ml absolute ethanol was refluxed for 5 h with the addition of a catalytic amount of piperidine. The yellow precipitate was filtered off and washed with ethanol and recrystallized from DMF.

**3-(1**,** 3-Diphenyl-1*H*-pyrazol-4-yl)-2-(4-(2-oxo-2*H*-chromen-3-yl)-thiazol-2-yl) acrylonitrile 8a**:

Yellow crystals; yield; 75%; m.p. 278–279 °C; IR (KBr, cm^−1^): 2211 (CN), 1719 (CO);^1^H NMR (DMSO*d*_*6*_): δ = 6.46–7.71 (m, 10 H, Ar), 7.79–7.94 (m, 4 H, Ar), 8.12 (s, 1 H, coumarin-H), 8.30 (s, 1 H, CH), 8.45 (s, 1 H, thiazole-H), 8.51 (s, 1 H, pyrazole-H); Anal. Calcd for C_30_H_18_N_4_O_2_S (243.33): C, 72.27; H, 3.64; N, 11.24; S, 6.43. Found: C, 72.47; H, 3.43; N, 11.48; S, 6.62%.

**2-(4-(2-Oxo-2*H*-chromen-3-yl) thiazol-2-yl)-3-(1-phenyl-3-(*p*-tolyl)-1*H*-pyrazol-4-yl) acrylonitrile 8b**:

Yellow crystals; yield; 73%; m.p. 284–286 °C; IR (KBr, cm^−1^): 2213 (NH), 1727 (CO);^1^H NMR (DMSO*d*_*6*_): δ = 2.42 (s, 3 H, CH_3_), 7.40–7.47 (m, 5 H, Ar), 7.59–7.68 (m, 5 H, Ar), 7.87–7.95 (m, 3 H, Ar), 8.27 (s, 1 H, CH), 8.48 (s, 1 H, thiazole-H), 8.72 (s, 1 H, coumarin-H ), 9.17 (s, 1 H, pyrazole-H);^13^C NMR (DMSO*d*_*6*_): δ = 21.41, 115.42, 116.49, 119.38, 119.88, 122.02, 124.70, 127.57, 128.03, 128.25, 128.62, 129.18, 129.71, 129.82, 131.17, 131. 90, 132.14, 136.63, 136.92, 139.10, 139.30, 139.40, 140.40; m/z = 512.29 (5.2%), 478.26 (4.3%), 395.12 (4.6%), 309,034 (3.1%), 207.31 (3.0%), 145.22 (12.4%), 115.25 (18.3%), 77 (100%); Anal. Calcd for C_31_H_20_N_4_O_2_S (512.29): C, 72.64; H, 3.93; N, 10.93; S, 6.26. Found: C, 72.46; H, 3.74; N, 10.69; S, 6.45%.

**3-(3-(4-Chlorophenyl)-1-phenyl-1*H*-pyrazol-4-yl)-2-(4-(2-oxo-2*H*-chromen-3-yl) thiazol-2-yl)acrylonitrile 8c**:

Yellow crystals; yield; 73%; m.p. 296–298 °C; IR (KBr, cm^−1^): 2212 (CN), 1741 (CO);^1^H NMR (DMSO*d*_*6*_): δ = 7.50–7.62 (m, 6 H, Ar), 7.69–7.73 (m, 5 H, Ar), 7.96–8.28 (m, 3 H, CH and Ar), 8.54 (s, 1 H, thiazole-H), 8.77 (s, 1 H, coumarin-H ), 9.65 (s, 1 H, pyrazole-H); m/z = 531.29 (10.1%), 492.38 (4.7%), 488.79 (2.2%), 443.11 (3.4%), 394.31 (18.4%), 319.34 (3.2%), 304.37 (7.2), 226.19 (6%), 204.18 (8%), 145.22 (29.5), 130.25 (12.6%), 115.20 (18%), 102.21 (96.5%), 77.21 (100%); Anal. Calcd for C_30_H_17_ClN_4_O_2_S (532.08): C, 67.60; H, 3.21; Cl, 6.65; N, 10.51; S, 6.02. Found: C, 67.41; H, 3.39; N, 10.75; S, 6.21%.

**3-(3-Hydroxyphenyl)-2-(4-(2-oxo-2*H*-chromen-3-yl) thiazol-2-yl)-acrylonitrile (9)**:

2-(4-(2-Oxo-2*H*-chromen-3-yl) thiazol-2-yl) acetonitrile **4** (0.01 mol) was added to 3-hydroxybenzaldehyde (0.01 mol) in 15 ml absolute ethanol. The mixture was refluxed with a catalytic amount of piperidine for 3 h. The yellow solid was collected and filtered then crystallized by a mixture of ethanol and DMF.

Pale yellow crystals; yield; 78%; m.p. 262–264 °C; IR (KBr, cm^−1^): 3412 (OH), 2216 (CN), 1721 (CO);^1^H NMR (DMSO*d*_*6*_): δ = 7.15–7.24 (m, 1 H, Ar), 7.35–7.41 (m, 1 H, Ar), 7.45 (t, 2 H, *J* = 7.7 Hz, Ar), 7.58 (t, 1 H, *J* = 7.7 Hz, Ar), 7.68 (d, 1 H, *J* = 7.6, Ar), 7.83 (d, 1 H, *J* = 7.7, Ar), 8.40 (s, 1 H, coumarin-H), 8.58 (s, 1 H, thiazole-H), 8.89–8.92 (m, 2 H, Ar), 10.30 (s, 1 H, OH);^13^C NMR (DMSO): 115.4, 116.3, 119.6, 120.6, 121.6, 123.8, 124.4, 125.2, 129.2, 129.6, 132.3, 132.6, 139.7, 147.3, 153.0, 153.2, 153.6, 159.3; Anal. Calcd for C_21_H_12_N_2_O_3_S (372.40): C, 67.73; H, 3.25; N, 7.52; S, 8.61. Found: C, 67.92; H, 3.43; N, 7.76; S, 8.80%.

##### Synthesis of compounds 11a, b

**Method A**:

Compound **9** (0.01 mol) was coupled with aryl diazonium chlorides **10a-c** (0.01 mol) under stirring at 0–5 ^o^C in DMF (5 ml) containing 0.01 mol of solid sodium hydroxide. The color solid so formed was filtered, washed with water, dried, and recrystallized from DMF.

**Method B**:

2-(4-(2-Oxo-2*H*-chromen-3-yl) thiazol-2-yl) acetonitrile **4** (0.01 mol) was reacted with arylazo of 3-hydrobenzaldehydes **12a-c** (0.01 mol) in 20 ml absolute ethanol containing few drops of piperidine. The mixture was refluxed for 4 h. The colored solid was collected and filtered then recrystallized from DMF.

**3-(5-Hydroxy-2-(-phenyldiazenyl) phenyl)-2-(4-(2-oxo-2*H*-chromen-3-yl) thiazol-2-yl) acrylonitrile (11a)**:

Orange crystals; yield; 65% (A), 70% (B); m.p. >300 °C; IR (KBr, cm^−1^): 3830 (OH), 2218 (CN), 1728 (CO);^1^H NMR (DMSO*d*_*6*_):δ = 6.86 (s, 1 H, Ar), 7.25–7.66 (m, 7 H, Ar), 8.08 (m, 2 H, Ar), 8.18 (s, 1 H, coumarin-H), 8.28 (s, 1 H, CH), 8.38 (s, 1 H, thiazole-H), 10.01 (s, 1 H, OH); Anal. Calcd for C_27_H_16_N_4_O_3_S (476.51): C, 68.06; H, 3.38; N, 11.76; S, 6.73. Found: C, 68.24; H, 3.58; N, 11.51; S, 6.92%.

**3-(5-Hydroxy-2-((4-methoxyphenyl) diazenyl) phenyl)-2-(4-(2-oxo-2*H*-chromen-3-yl) thiazol-2-yl)acrylonitrile (11b)**:

Deep orange crystals; yield; 68% (A), 74% (B); m.p. >300 °C; IR (KBr, cm^−1^): 3910 (OH), 2220 (CN), 1723 (CO);^1^H NMR (DMSO*d*_*6*_):δ = 3.87 (s, 3 H, OCH_3_), 7.13–7.18 (m, 2 H, Ar), 7.43–7.51 (m, 3 H, Ar), 7.84-8.0 (m, 5 H, Ar), 8.55–8.57 (m, 2 H, Ar), 8.87 (s, 1 H, coumarin-H), 8.93 (s, 1 H, thiazole-H), 9.10 (s, 1 H, OH); Anal. Calcd for C_22_H_18_N_4_O_4_S (506.53): C, 66.39; H, 3.58; N, 11.06; S, 6.33. Found: C, 66.58; H, 3.76; N, 11.32; S, 6.53%.

##### Synthesis of compounds 15a, b

Compound **4** (0.01 mol) was refluxed with salicyalaldehydes **13a**,** b** (0.01 mol) in 15 ml absolute ethanol with a few drops of piperidine. The mixture was refluxed for 7 h. The solid so formed was filtered and recrystallized from DMF.

**3-(2-(2-Imino-2*H*-chromen-3-yl) thiazol-4-yl)-2*H*-chromen-2-one (15a)**:

Red crystals; yield; 70%; m.p. >300 °C; IR (KBr, cm^−1^): 3233 (NH), 1723 (CO);^1^H NMR (DMSO*d*_*6*_):δ = 7.01 (d, 1 H,*J* = 6.8 Hz, Ar), 7.45–7.50 (m, 1 H, Ar), 7.59–7.62 (m, 1 H, Ar), 7.75 ( d, 1 H, *J* = 8.6 Hz, Ar), 7.86–7.93 (m, 3 H, Ar), 7.86–7.93 (m, 3 H, Ar), 8.14 ( d, 1 H, *J* = 8.4 Hz, Ar), 10.54 (s, 1 H, NH); Anal. Calcd for C_21_H_12_N_2_O_3_S (372.40): C, 67.73; H, 3.25; N, 7.52; S, 8.61. Found: C, 67.55; H, 3.44; N, 7.77; S, 8.43%.

**3-(2-(6-Hydroxy-2-imino-2*H*-chromen-3-yl) thiazol-4-yl)-2*H*-chromen-2-one (15b)**:

Red crystals; yield; 73%; m.p. >300 °C; IR (KBr, cm^−1^): 3240 (NH), 1737 (CO);^1^H NMR (DMSO*d*_*6*_):δ = 7.53–7.57 (m, 3 H, Ar), 7.66–7.69 (m, 1 H, Ar), 7.90 (d, 2 H, *J* = 8.6 Hz, Ar), 7.98-8.0 (m, 4 H, Ar), 11.66 (s, 1 H, NH); 12.79 (s, 1 H, OH); Anal. Calcd for C_21_H_12_N_2_O_4_S (388.40): C, 64.94; H, 3.11; N, 7.21; S, 8.25. Found: C, 64.76; H, 3.31; N, 7.46; S, 8.44%.

##### Synthesis of compounds 18a-d

Compound **4** (0.01 mol) was heated with salicyalaldehydes **16a-d** (0.01 mol) in 20 ml absolute ethanol with a few drops of piperidine. The reaction mixture was refluxed for 7 h. The colored solid was filtered off and recrystallized from DMF.

**3-(2-(2-Imino-6-(phenyldiazenyl)-2*H*-chromen-3-yl) thiazol-4-yl)-2*H*-chromen-2-one (18a)**:

Brownish red crystals; yield; 67%; m.p. >300 °C; IR (KBr, cm^−1^): 3310 (NH), 1723 (CO);^1^H NMR (DMSO*d*_*6*_):δ = 7.43–7.50 (m, 5 H, Ar), 7.63–7.74 (m, 4 H, Ar), 7.90–8.11 (m, 2 H, Ar), 7.68–7.84 (m, 2 H, Ar), 8.31 (s, 1 H, coumarin-H), 8.59 (s, 1 H, thiazole-H), 9.31 (s, 1 H, NH); Anal. Calcd for C_27_H_16_N_4_O_3_S (476.51): C, 68.06; H, 3.38; N, 11.76; S, 6.73. Found: C, 68.26; H, 3.57; N, 11.50; S, 6.91%.

**3-(2-(2-Imino-6-(*p*-tolyldiazenyl)-2*H*-chromen-3-yl) thiazol-4-yl)-2*H*-chromen-2-one (18b)**:

Brownish red crystals; yield; 67%; m.p. >300 °C; IR (KBr, cm^−1^): 3310 (NH), 1723 (CO);^1^H NMR (DMSO*d*_*6*_):δ = 2.43 (s, 3 H, CH_3_), 7.43–7.67 (m, 3 H, Ar), 7.81–7.95 (m, 4 H, Ar), 8.02–8.28 (m, 2 H, Ar), 8.49–8.54 (m, 2 H, Ar), 8.70 (s, 1 H, coumarin-H), 8.83 (s, 1 H, thiazole-H), 9.30 (s, 1 H, NH); m/z = 589.31 (M^+ 1^-1, 5%), 371.24 (6.4%), 355 (3.6%), 346 (4.14%), 283 (3.12%), 228 (5.16%), 210 (5.63), 200 (6.94%), 145 (19.08), 130 (8.32%), 118 (15.93%), 91 (100%); Anal. Calcd for C_28_H_18_N_4_O_3_S (490.54): C, 68.56; H, 3.70; N, 11.42; S, 6.54. Found: C, 68.28; H, 3.51; N, 11.67; S, 6.71%.

**3-(2-(2-Imino-6-(4-methoxyphenyldiazenyl)-2*H*-chromen-3-yl) thiazol-4-yl)-2*H*-chromen-2-one (18c)**:

Red crystals; yield; 67%; m.p. >300 °C; IR (KBr, cm^−1^): 3310 (NH), 1724 (CO);^1^H NMR (DMSO*d*_*6*_):δ = 3.89 (s, 3 H, OCH_3_), 7.12–7.18 (m, 3 H, Ar), 7.43–7.51 (m, 3 H, Ar), 7.67–7.71 (m, 1 H, Ar), 7.84 (d, 1 H, *J* = 8.8 Hz, Ar), 7.92–7.95 (m, 4 H, Ar), 8.27 (s, 1 H, coumarin-H), 8.87 (s, 1 H, thiazole-H), 9.30 (s, 1 H, NH); m/z = 589.31 (M^+ 1^-1, 5%), 371.24 (6.4%), 355 (3.6%), 346 (4.14%), 283 (3.12%), 228 (5.16%), 210 (5.63), 200 (6.94%), 145 (19.08), 130 (8.32%), 118 (15.93%), 91 (100%); Anal. Calcd for C_28_H_18_N_4_O_4_S (506.54): C, 66.39; H, 3.58; N, 11.06; S, 6.33. Found: C, 68.28; H, 3.51; N, 11.67; S, 6.71%.

**3-(2-(6-((4-Chlorophenyl) diazenyl)-2-imino-2*H*-chromen-3-yl) thiazol-4-yl)-2*H*-chromen-2-one (18d)**:

Brownish red crystals; yield; 70%; m.p. >300 °C; IR (KBr, cm^−1^): 3320 (NH), 1726 (CO);^1^H NMR (DMSO*d*_*6*_):δ = 7.16 (d, 1 H, *J* = 8.8 Hz, Ar), 7.32–7.45 (m, 3 H, Ar), 7.56–7.65 (m, 4 H, Ar), 7.81–7.88 (m, 3 H, Ar), 7.94 (d, 1 H, *J* = 8.8 Hz, Ar), 8.30 (s, 1 H, coumarin-H), 8.58 (s, 1 H, thiazole-H), 9.31 (s, 1 H, NH); Anal. Calcd for C_27_H_15_NCl_4_O_3_S (510.95): C, 63.47; H, 2.71; Cl, 6.94; N, 10.97; S, 6.27. Found: C, 63.28; H, 2.51; N, 10.71; S, 6.45%.

### Biological part

#### Drug delivery

##### Preparation of Coumarin derivatives encapsulated into nanostructured lipid-chitosan conjugates carrier (NLC-CS)

The NLC-CS was synthesized by hot homogenization-ultrasonication method^76^ with some modifications as follows, about 1.5 g of stearic acid and 3 g oleic acid were placed in a 50 ml beaker (B1, oil phase) and heated up to 72 °C utile all the solid lipid converted into a homogeneous melt was obtained then the temperature adjusted to be fixed using a water bath (80–85 °C). In another 50 ml beaker (B2, drug), 30 mg of coumarin derivatives dissolved in 5 ml of methanol-chloroform mixture (1:3) with heating utile all coumarin derivative were completely soluble. On hot (B2) added to (B1) with heating to make sure that all solvents have been eliminated (use the digital balance to weigh the net solution before and after solvent addition) and complete clear molten obtained (B3). In the last beaker (B4, aqueous phase), 4 ml chitosan 1% (stock solution prepared by dissolving 1 g of low M.W chitosan in 100 ml 2% acetic acid), 10 mL pluronic F127 (stock solution prepared by dissolving 1 g in 100 ml distilled water) and 10 ml tween 20 were added and well-stirred for 5 min. Add (B4) to (B3) with stirring until clear microemulsion obtained, then attain the final mixture to cool by adding 30 ice-old water with sonication using probe sonicator (400 W, time interval 4s) for 10 min in ice bath. The final dispersion was completed to  100 ml. Without cry-protectant, the dispersion converted to a semi-solid substance via freeze lyophilization for two days at -50 °C to perform the required investigations.

#### Particle size and surface charge

The hydrodynamic size and polydispersity index (PDI) measurements were evaluated with the assistance of the Dynamic Light Scattering technique (DLS) at the angle of 173° at room temperature. A crucial measure to test the quality and stability of nanoparticles through the manifestation of electrochemical equilibrium between particles and the liquid system^51,54^. Zeta potential (z.e) is monitored by measuring the frequency of the scattered light shifting at 12°. The particle size and PDI were measured at the Egyptian Petroleum Institute (EPRI), Cairo, Egypt using (Zeta sizer Nano series (HT), Nano ZS, Malvern Instruments; UK). Zeta potential was measured by (NanoBrookZetapals particle sizing software ver.5.23, Brookhaven’s Instrument Corp., Holtsville, NY, USA). About 5–10 mg of semi-solid lyophilized nanoparticles were dissolved in 10 mL distilled water, sonication was applied for 5 min until homogeneous dispersion was obtained and the required quantity was placed in a quartz cell to be analyzed.

#### NLC surface morphology by transmission electron microscope (TEM)

The internal morphology manifestation of the prepared nanoparticles is one of the crucial fundamental analyses used to detect the homogeneity and regularity of the prepared NPs. Internal morphology visualization was examined by field transmission electron microscopy (HR-TEM, JSM-7100 F) in the Egyptian Petroleum Research Institute (EPRI), Cairo, Egypt. The images were recorded with JEOL JEM-2100-115 high-resolution transmission electron microscopes with an accelerating voltage of 200 kV. One microliter of the prepared NPs was diluted with distilled water in a ratio (1:200) and placed on a 200 mesh carbon-coated grid. The solution was attained two minutes after removing excess using a cellulose filter. For negative staining, a drop of 2% (w/w) phosphotungstic acid PTA was added to the grid for 10 s and the excess was removed by filter paper^55,76^.

#### Drug encapsulation efficacy (EE)

The encapsulation (entrapment) efficiency of **8b**-NLC-Cs NPs was determined by the indirect ultrafiltration centrifugation method^95–97^. One milliliter of freshly prepared Coum-NLC-Cs NPs was placed in a vivspin20 centrifugal concentrator tube (MWCO 5k Da) and centrifuged at 4500 rpm for 20 min at 4 °C. The supernatant, which contains free or non-encapsulated drug, was separated by decantation and dissolved in DMSO with the assistance of probe sonication (Scientz, ultrasonic homogenizer-HD, Ningbo Scientz Biotechnology Co., Ltd., China) utile clear solution obtained. The non-encapsulated drug concentration was then determined by a UV spectrophotometer (Genway spectrophotometer 6305, Japan), DMSO was used as blank and a known concentration of **8b** in DMSO was prepared as a standard and measurements was done at 450 nm using 1 cm glass cuvette at room temperature and consequently, non-encapsulated drug (free drug) calculated from:

$$\frac{{{\text{Absorbance}}\,\,{\text{of}}\,\,{\text{the}}\,\,{\text{sample}}\,\,{\text{(free}}\,\,{\text{drug}}\,\,{\text{or}}\,\,{\text{supernatent)}}}}{{{\text{Absorbance}}\,\,{\text{of}}\,\,{\text{standard}}\,\,{\text{8b}}}} \times {\text{standard}}\,\,{\text{concentration}}$$

Next, the amount of the entrapped drug was obtained by subtracting the amount of free drug from the total drug incorporated in 1 ml dispersion we started before as follows:

Entrapped drug = Total drug incorporated - free drug (supernatant).

Therefore, the entrapment efficiency (EE) and drug loading capacity (DL) were determined by the following equations:

$$\begin{gathered} {\text{EE}}\% =\frac{{{\text{Amount of}}\,\,{\text{the Entrapped}}\,\,{\text{Drug}}}}{{{\text{Amount}}\,\,{\text{of}}\,\,{\text{Total}}\,\,{\text{Drug}}\,\,{\text{Added}}}} \times 100 \hfill \\ DL\% =\frac{{{\text{Amount of}}\,\,{\text{the Entrapped}}\,\,{\text{Drug}}}}{{{\text{}}\,\,{\text{nanoparticle}}\,{\text{weight}}}} \times 100 \hfill \\ \end{gathered}$$

As some drugs are adsorbed to the ultrafiltration membrane to some extent^98^, that makes an error in the estimation of the entrapped drug due to high the hydrophobicity of **8b**, the drug absorbability to the ultrafiltration membrane was investigated by measuring the filtration of simple drug solution of known concentrations through the membrane and measuring drug concentrations in the ultra-filtrate to determine the adsorbed drug and avoid it in all measurements.

#### In-vitro antimicrobial screening (inhibition zone)

The most common technique for determining antimicrobial susceptibility testing is the well diffusion test. The quantitative result showed inhibition zones in millimeters. The CLSI (Clinical and Laboratory Standards Institute) has proposed a well-diffusion method for susceptibility tests. One significant advantage of this disk diffusion method is that results can be obtained after 16 to 48 h of incubation (a shorter incubation time than by the M38)^99,100^. Antimicrobial activity was assessed using the agar well diffusion method against strains of *Bacillus Subtilis* (ATCC 6633), *Staphylococcus Aureus* (ATCC 6538), *Escherichia coli* (ATCC 8739), *Pseudomonas aeruginosa* (ATCC 90274), *Salmonella typhi*,* Candida albicans (*ATCC 10221*)*. The agar plate surface was inoculated by spreading a part of the microbial inoculum over the entire agar surface. A hole with a diameter of 6 to 8 mm was punched aseptically with a sterile cork borer or a tip, and a volume of 100 µl of the antimicrobial agent solution (under investigation) at a specific concentration was introduced into the well. Then, the plates were incubated under suitable conditions depending on the tested microorganism. The antimicrobial agent diffuses in the agar medium and inhibits the growth of the microbial strain tested^101^. The incubation of the inoculated microdilution tubes or microdilution trays was performed at 35 ± 2 °C for 16–48 h depending on the nature of the microbial strain, in an ambient air incubator within 15 min of adding the inoculum. After incubation times, the resulting inhibition zone diameters (in mm) surrounding the wells should be measured to the nearest whole millimeter at the point at which there is a prominent reduction in growth. NA expressed no activity, 10 mg/ml of all samples were dissolved in DMSO. Gentamycin was used as a positive control.

#### Determination of minimal inhibitory concentration (MIC)

The minimum inhibitory concentration is shown to be the lowest concentration of an antimicrobial agent that prevents visible growth of a microorganism in broth dilution susceptibility test. Based on the broth micro dilution method^102^, two-fold dilutions of antimicrobial agent in broth were prepared. Where 10.0 mg of each sample was dissolved in 10 ml distilled water (1000 µg/ml) was prepared as a stock solution and serial two-fold dilutions indexed to the base 2 (1000, 500, 250, 125,…0.061 µg/ml) were prepared. Dispense the antimicrobial/broth solutions into plastic microdilution trays. The most convenient method of preparing microdilution trays is by using the dispensing device and antimicrobial dilutions made in at least 10 ml of broth. The dispensing device then delivers 0.1 (± 0.02) ml into each 96 well of a standard tray. Turbidity indicates growth of the microorganism and the MIC is the lowest concentration where no growth is visually observed.

## Conclusion

Novel coumarin-based compounds were synthesized and characterized. All target compounds investigated their antimicrobial properties and tested against *B. subtilis*,* S. aureus*,* E. coli*,* P. aeruginosa*,* S. typhi*, and *C. albicans*. Coumarin derivatives were effective against *S. typhi among other tested bacteria*. The most promising compounds **4**, **6d**, and **8b** were selected encapsulated in vicinity of nanostructured lipid carrier and chitosan (NLC-Cs). The antimicrobial investigations were performed again for **4**-NLC-Cs, **6d**-NLC-Cs, and **8b**-NLC-Cs. After nanoformulation, all nanoparticles, especially **8b**-NLC-Cs, showed potent effects against all organisms tested. Furthermore, compound **8**-NLC-Cs exhibited minimum inhibitory concentration (MIC) = 0.48 and 0.24 µg/ml against *S. aureus* and *C. albicans*, respectively. This means that the MIC were inhabited 65 times more than the MIC before nano. Additionally, when compounds **4**,** 6d**, and **8b** were assessed against DNA-Gyrase enzyme, **8b** showed a perfect inhibition with IC_50_ = 4.56 µM. TEM images revealed different stages of structural defects of cell wall lysis and spit its components out after treatment with the modified and developed coumarin-NLC-Cs analogous, and required results were obtained.

## Electronic supplementary material

Below is the link to the electronic supplementary material.

Supplementary Material 1

## Data Availability

All data generated or analyzed during this study are included in this current article and its supplementary information files.
