# Supplementary material for: Design, synthesis of some novel coumarins and their nanoformulations into lipid-chitosan nanocapsule as unique antimicrobial agents
Source: Sci Rep. 2024 Dec 23;14:30598. doi: 10.1038/s41598-024-79861-7 (PMC11666591; doi:10.1038/s41598-024-79861-7)
Supplement: Supplementary file 1 — Supplementary Material 1 [file 41598_2024_79861_MOESM1_ESM.docx]

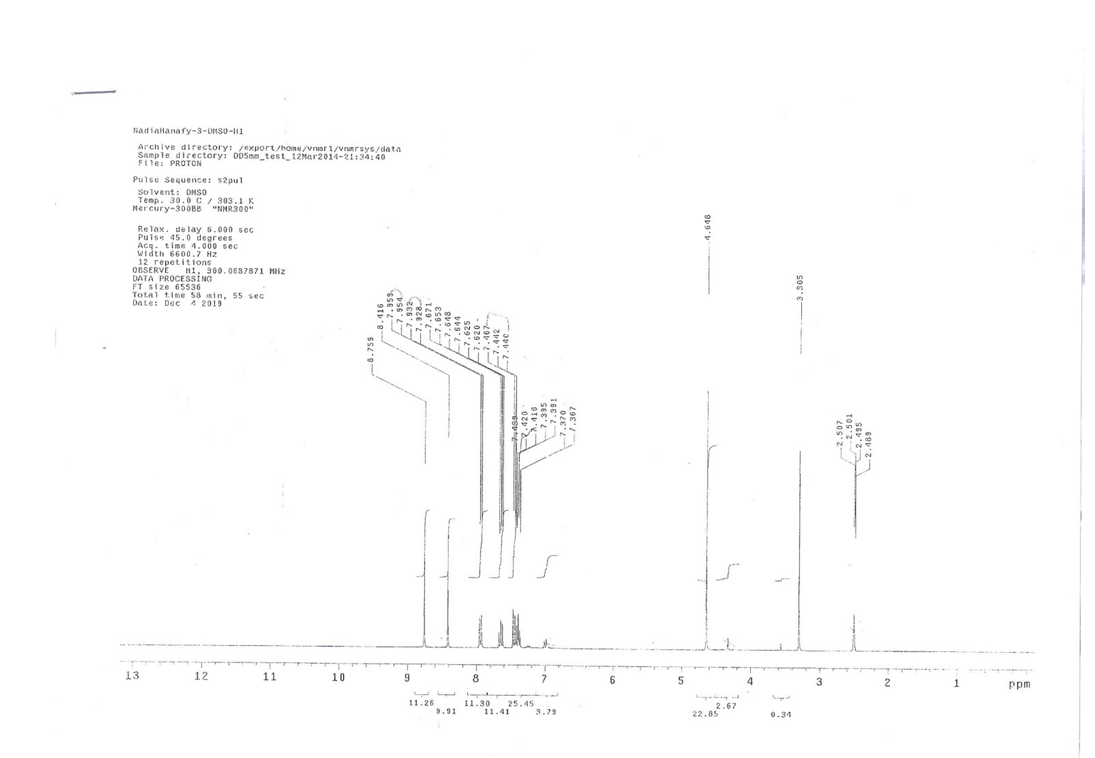


**S1. ^1^H NMR of compound 4.**

**
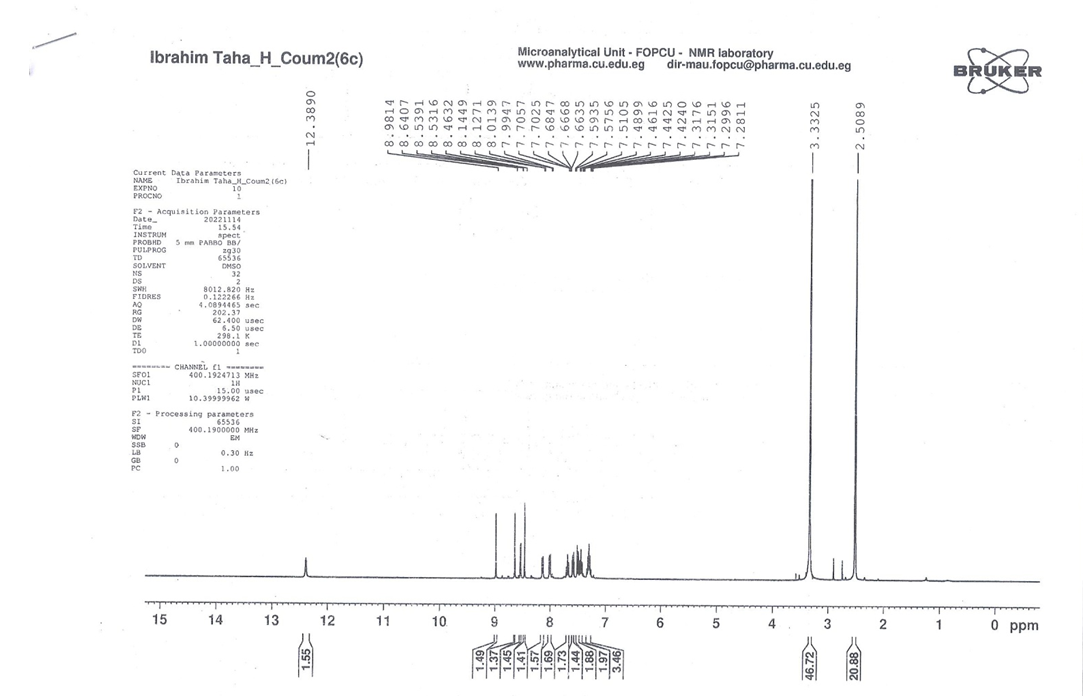
**

**S2. ^1^H NMR of compound 6c**

**
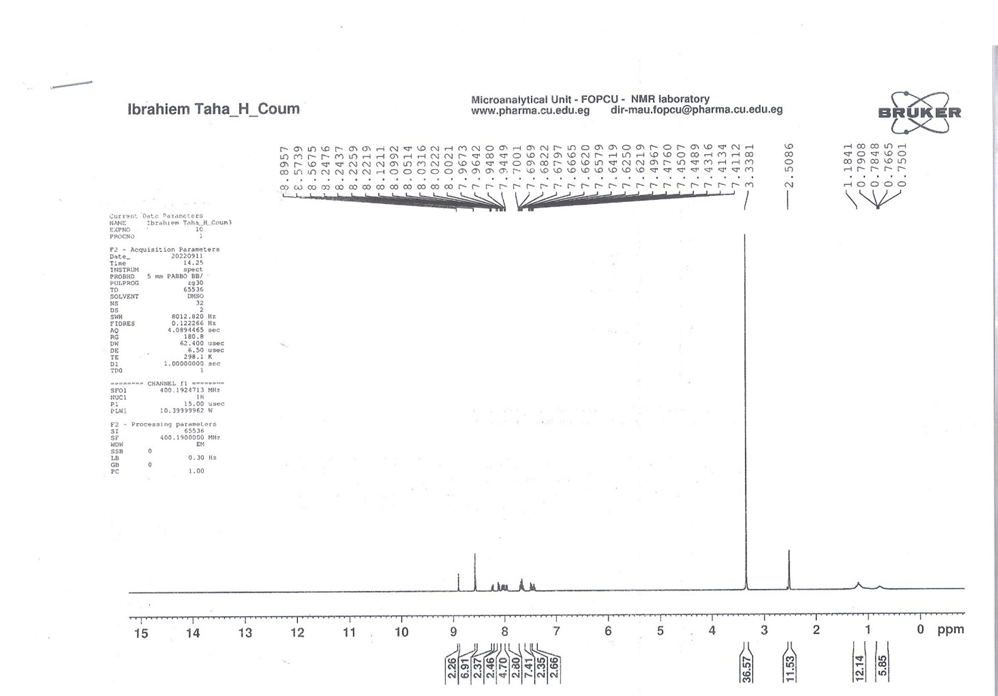
**

**S3. ^1^H NMR of compound 6d**

**
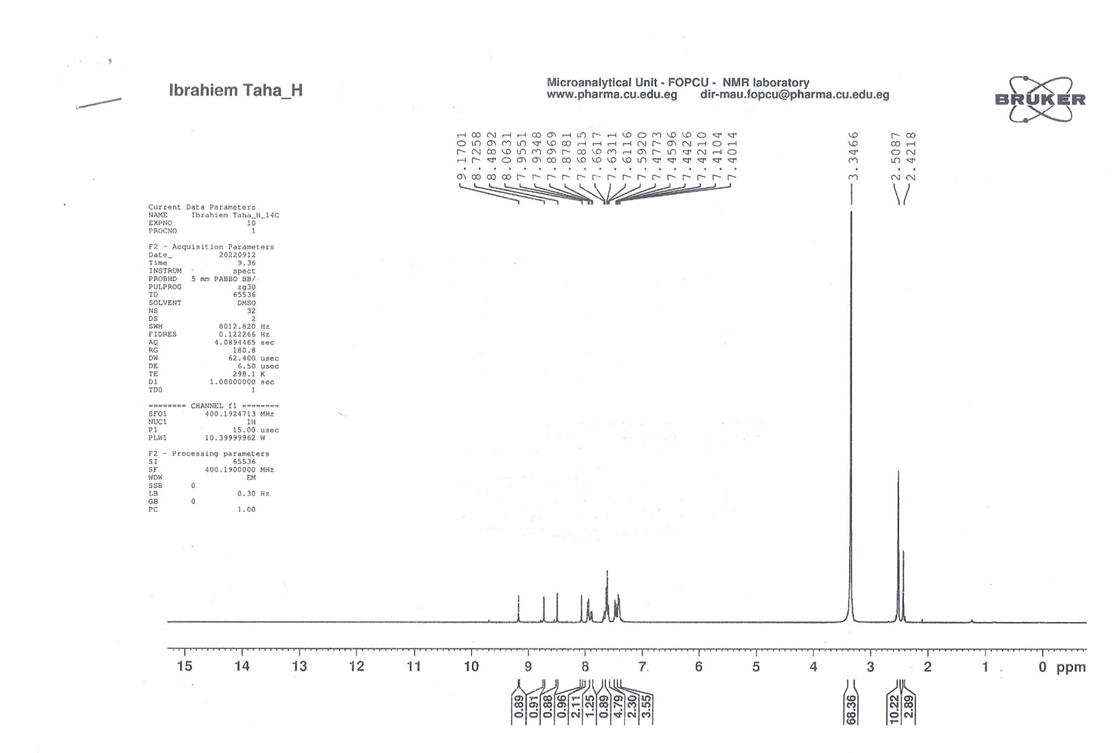
**

**S4. ^1^H NMR of compound 8b**

**
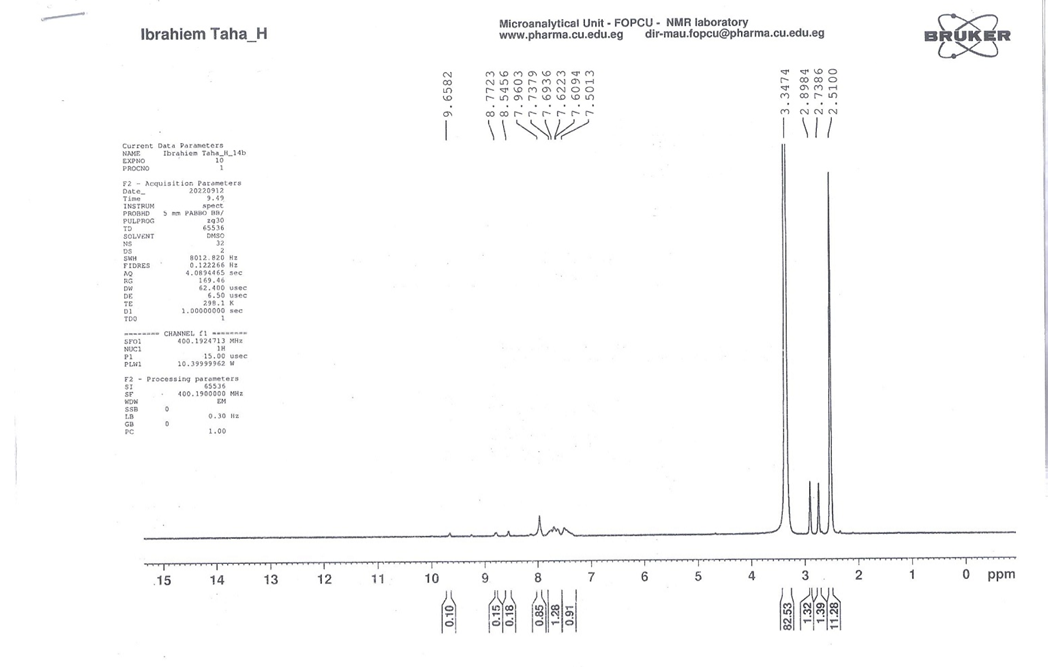
**

**S5. ^1^H NMR of compound 8c**

**
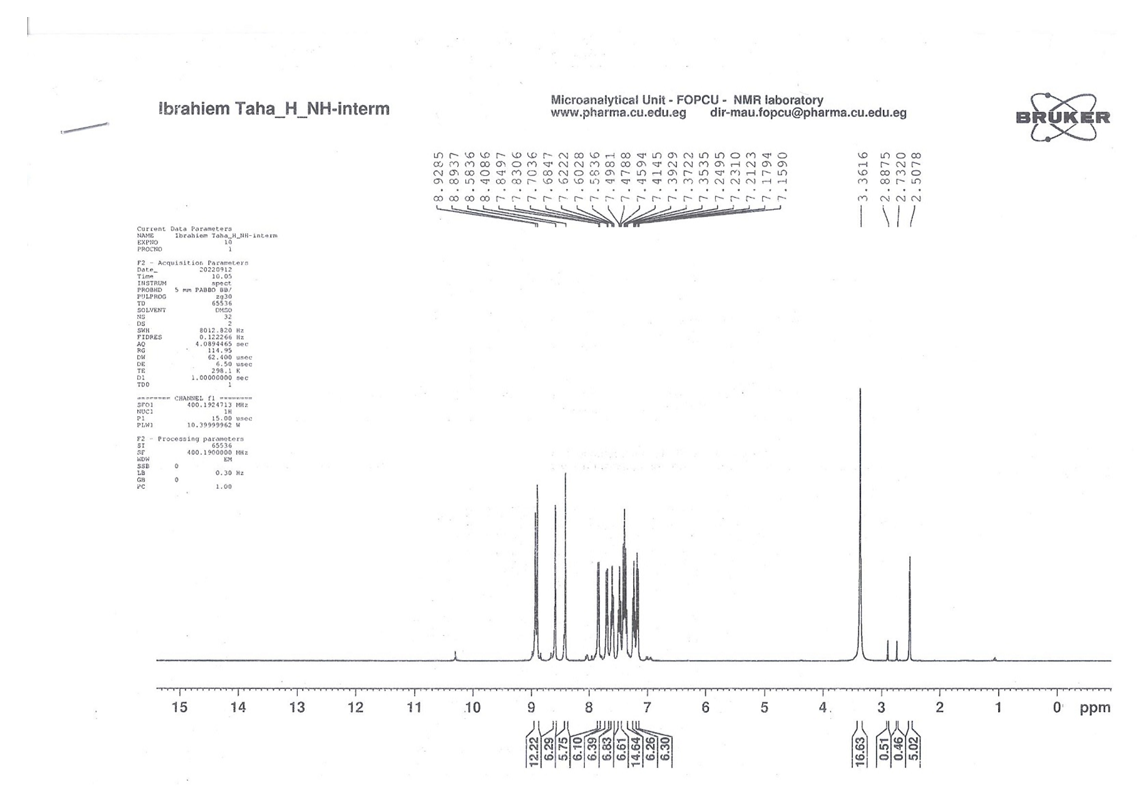
**

**S6. ^1^H NMR of compound 9**

**
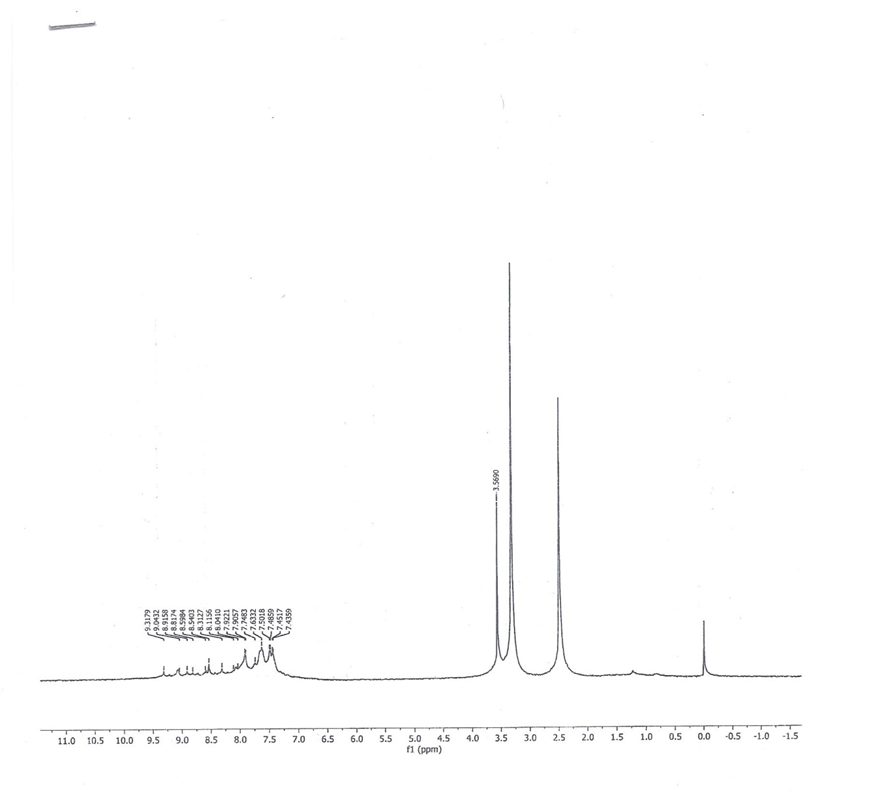
**

**S7. 1H NMR of compound 11a**

**
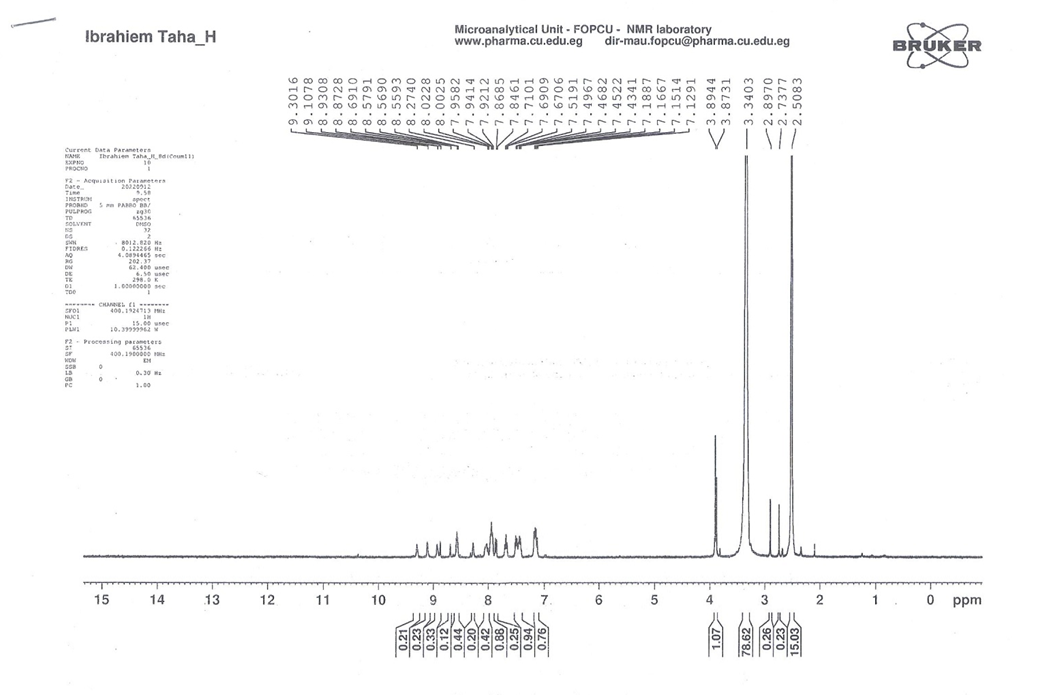
**

**S8. ^1^H NMR of compound 11b**

**
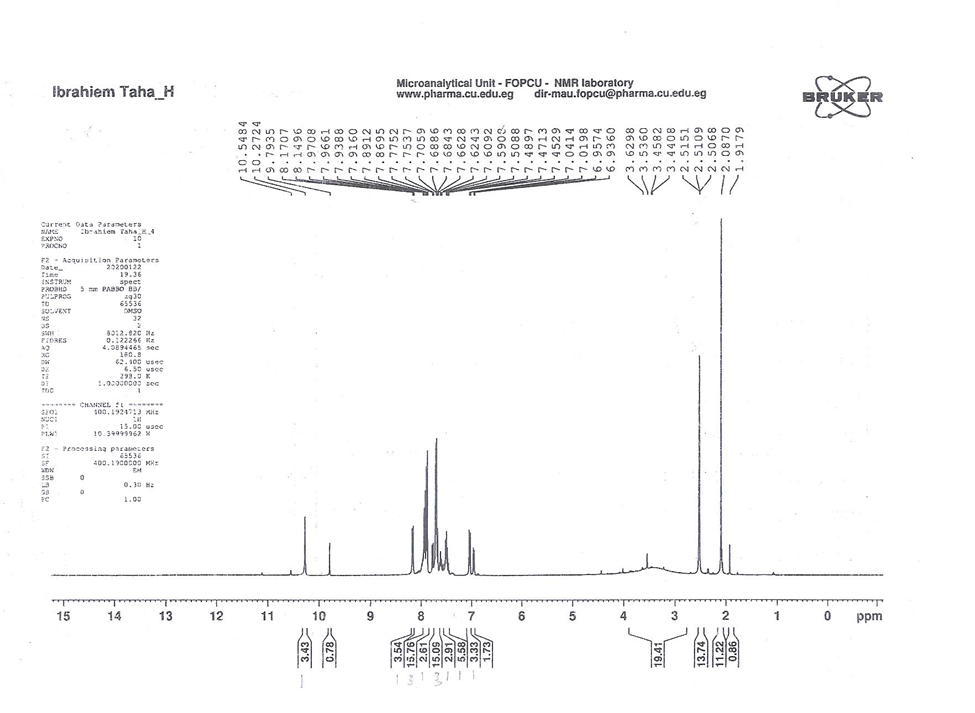
**

**S9. ^1^H NMR of compound 15a**


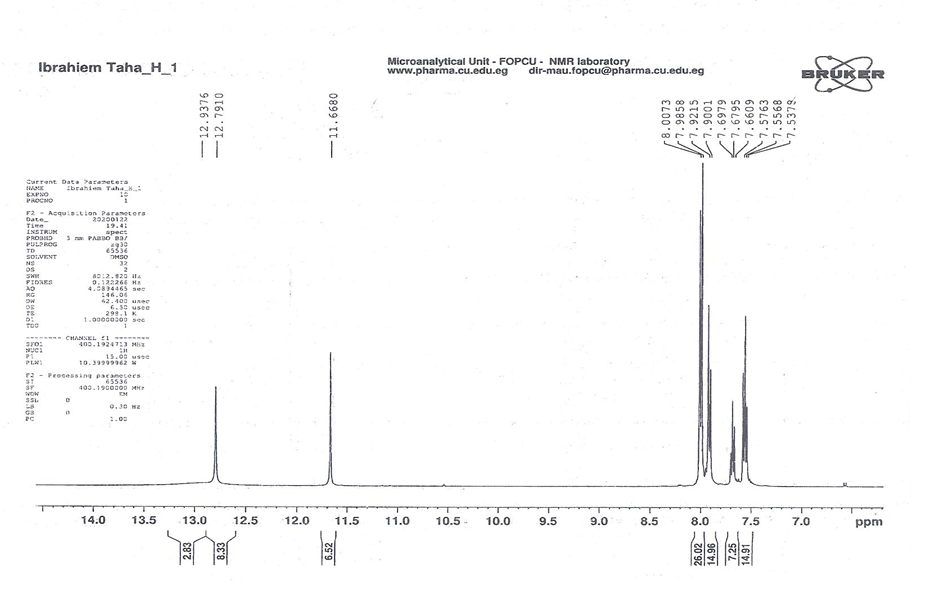


**S10. ^1^H NMR of compound 15b**

**
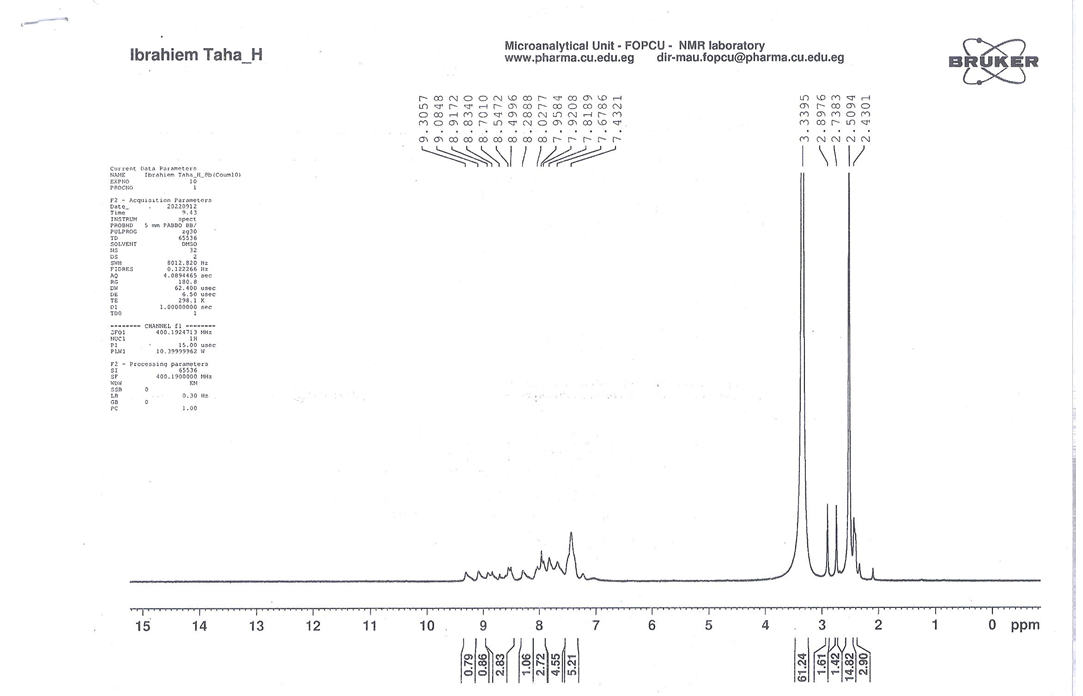
**

**S11. ^1^H NMR of compound 18b**

**
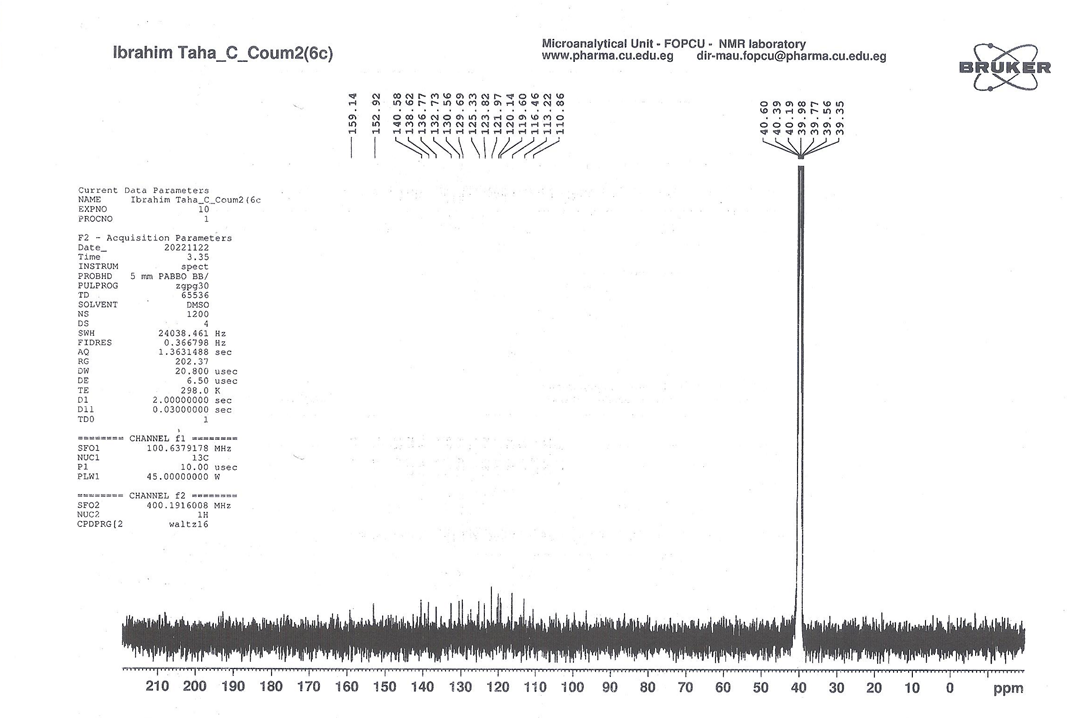
**

**S12. ^13^C NMR of compound 6c**

**
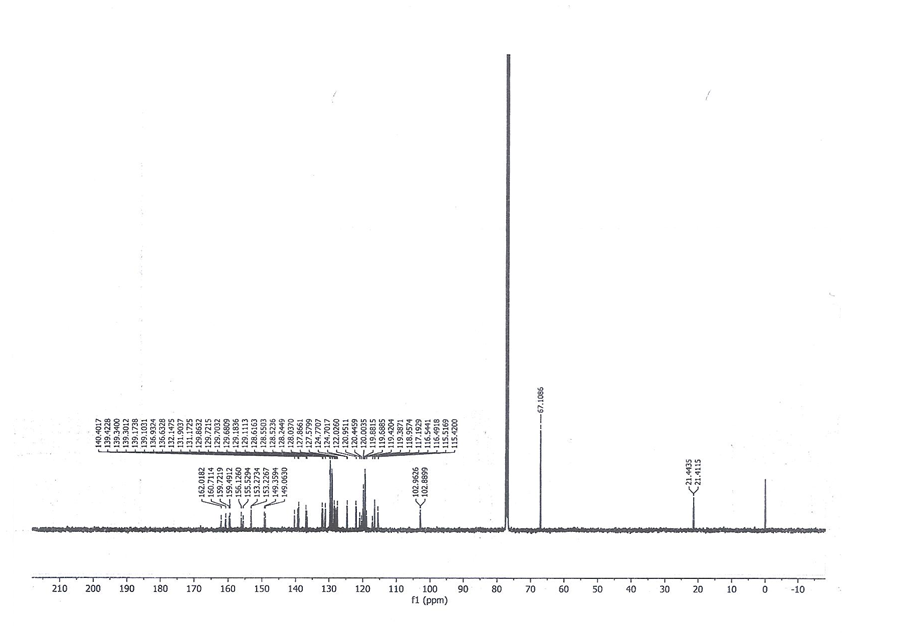
**

**S13. ^13^C NMR of compound 8b**

**
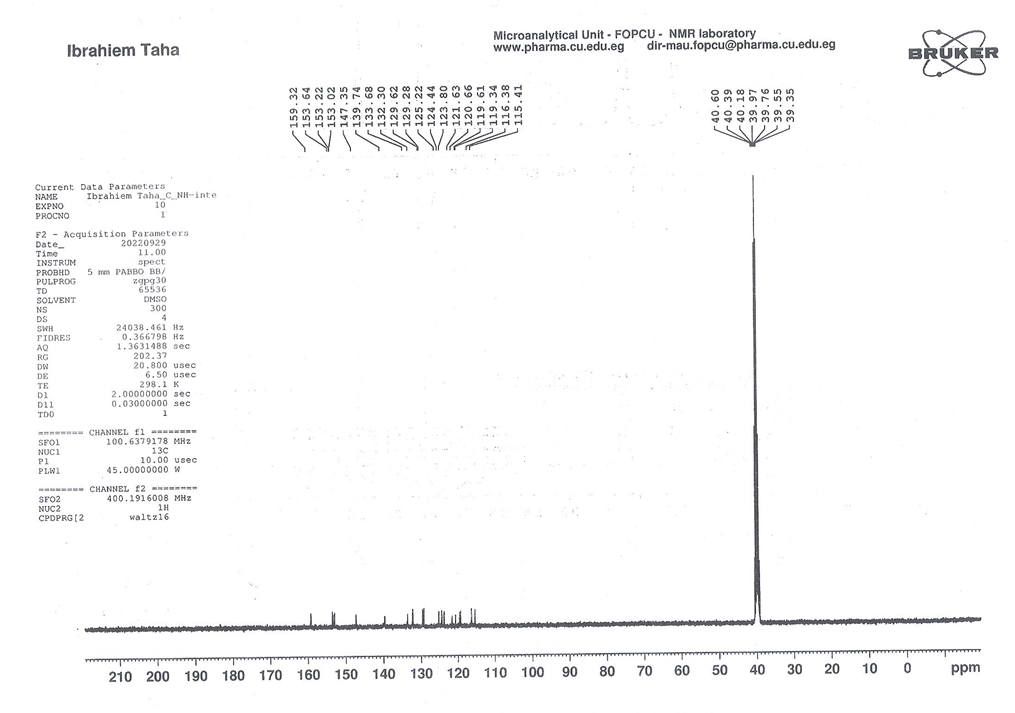
**

**S14. ^13^C NMR of compound 9**

**
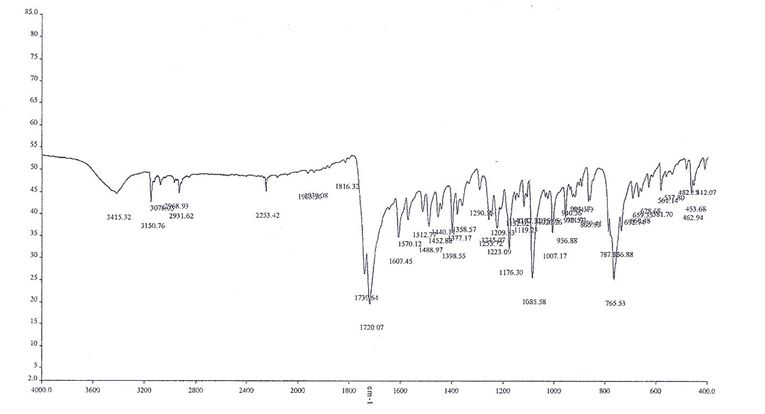
**

**S15. IR of compound 4**

**
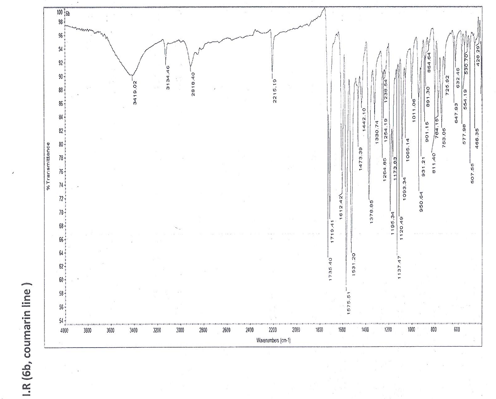
**

**S16. IR of compound 6b**

**
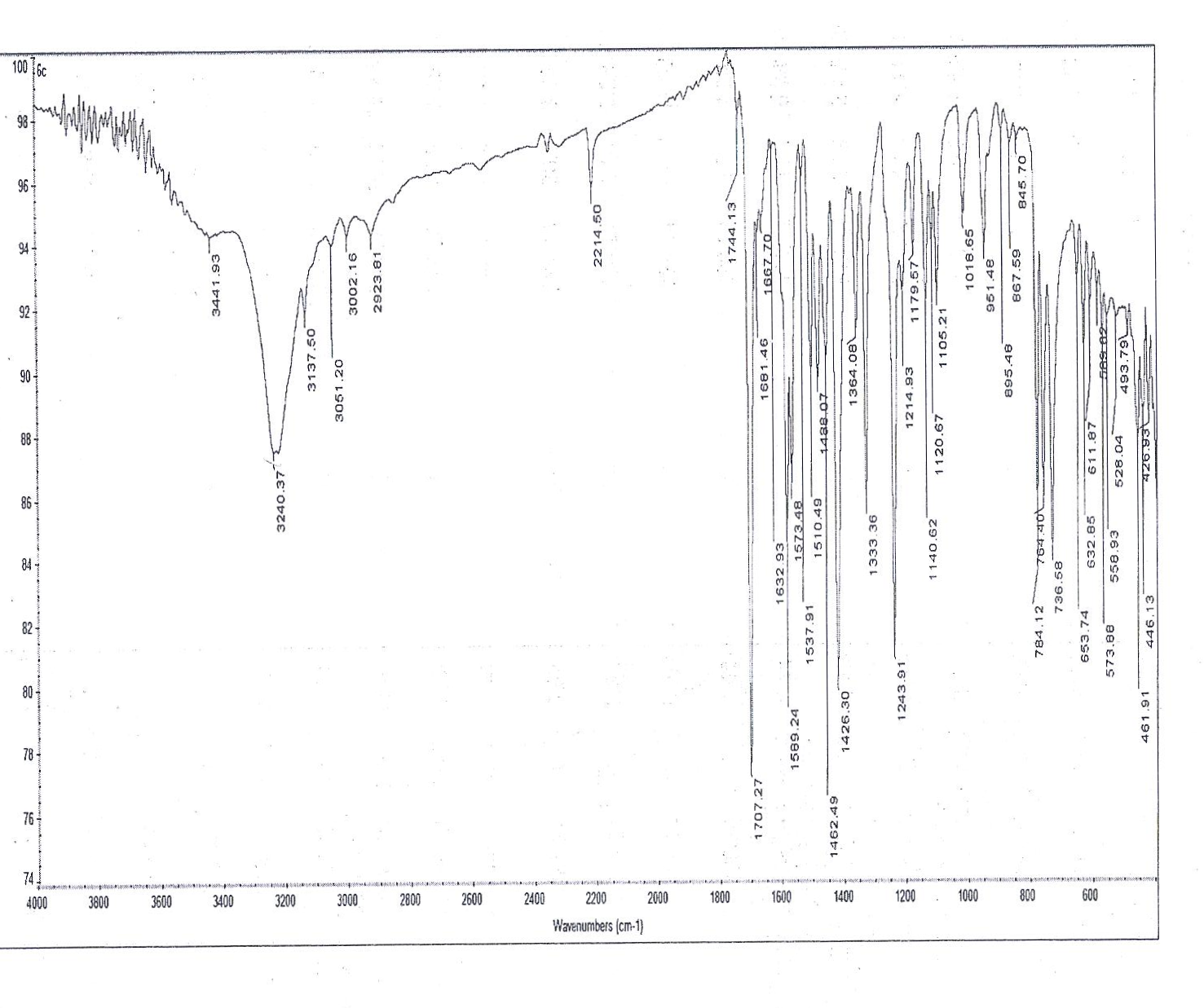
**

**S17. IR of compound 6c**

**
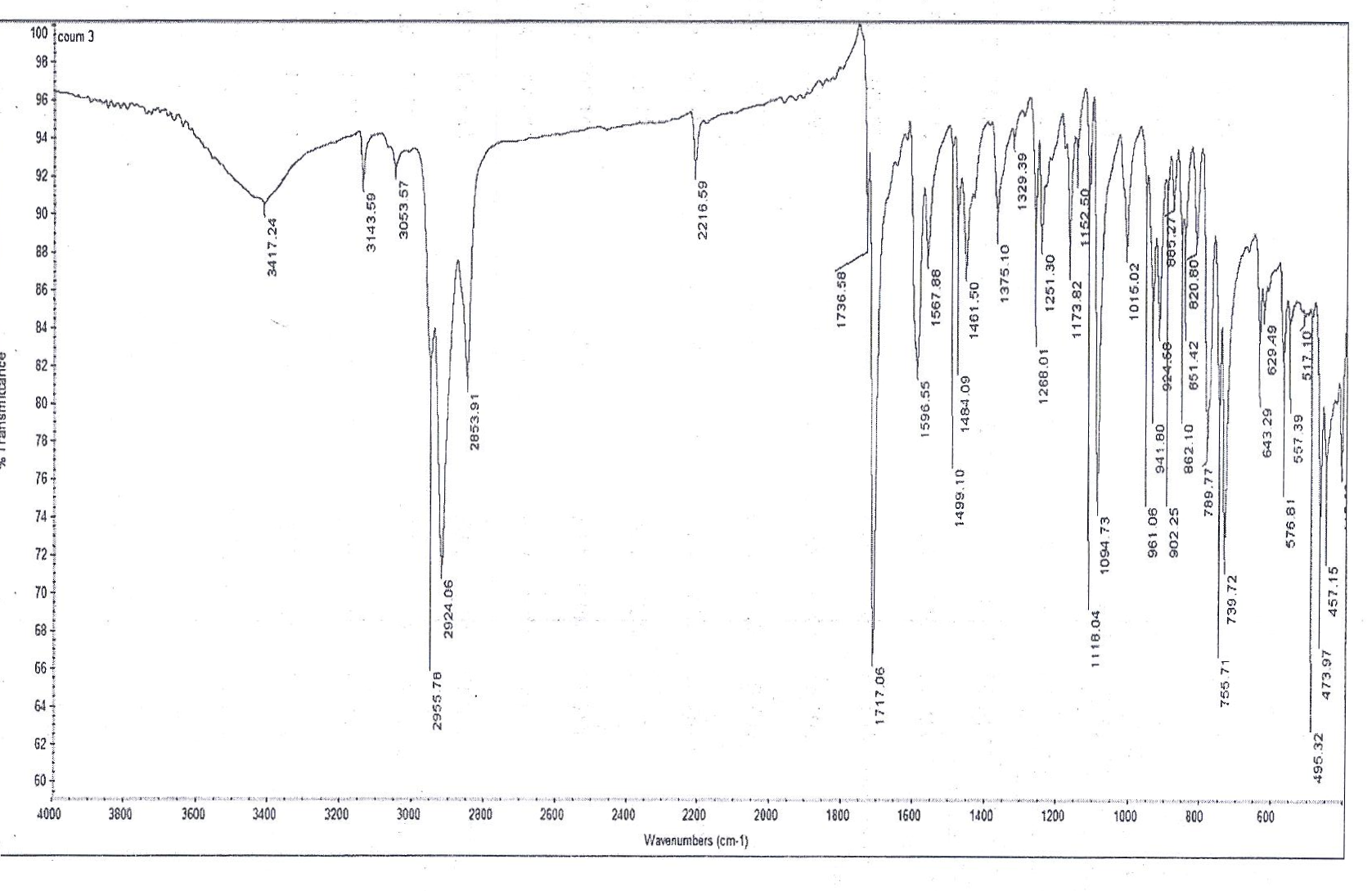
**

**S18. IR of compound 6d**

**
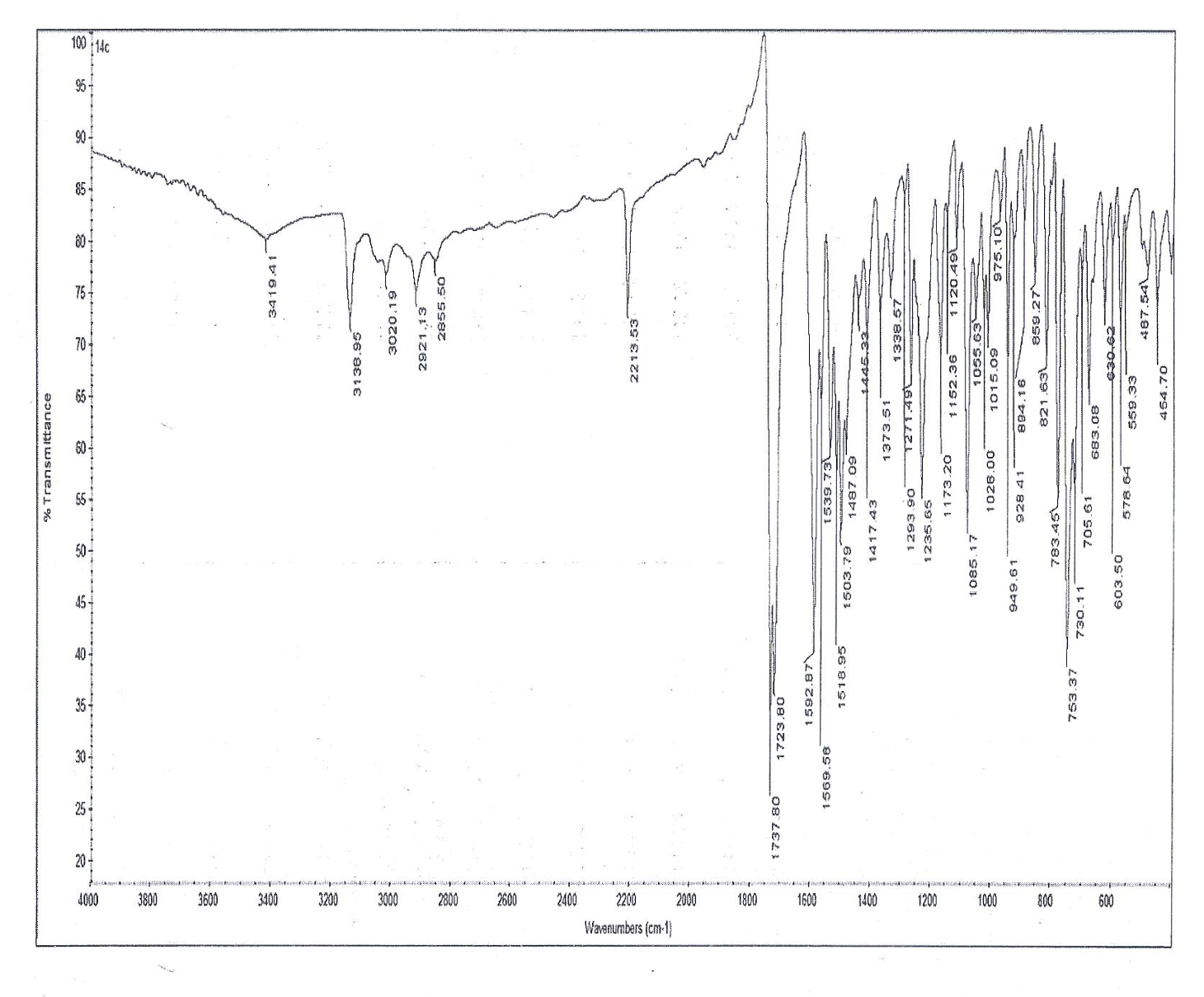
**

**S19. IR of compound 8b**

**
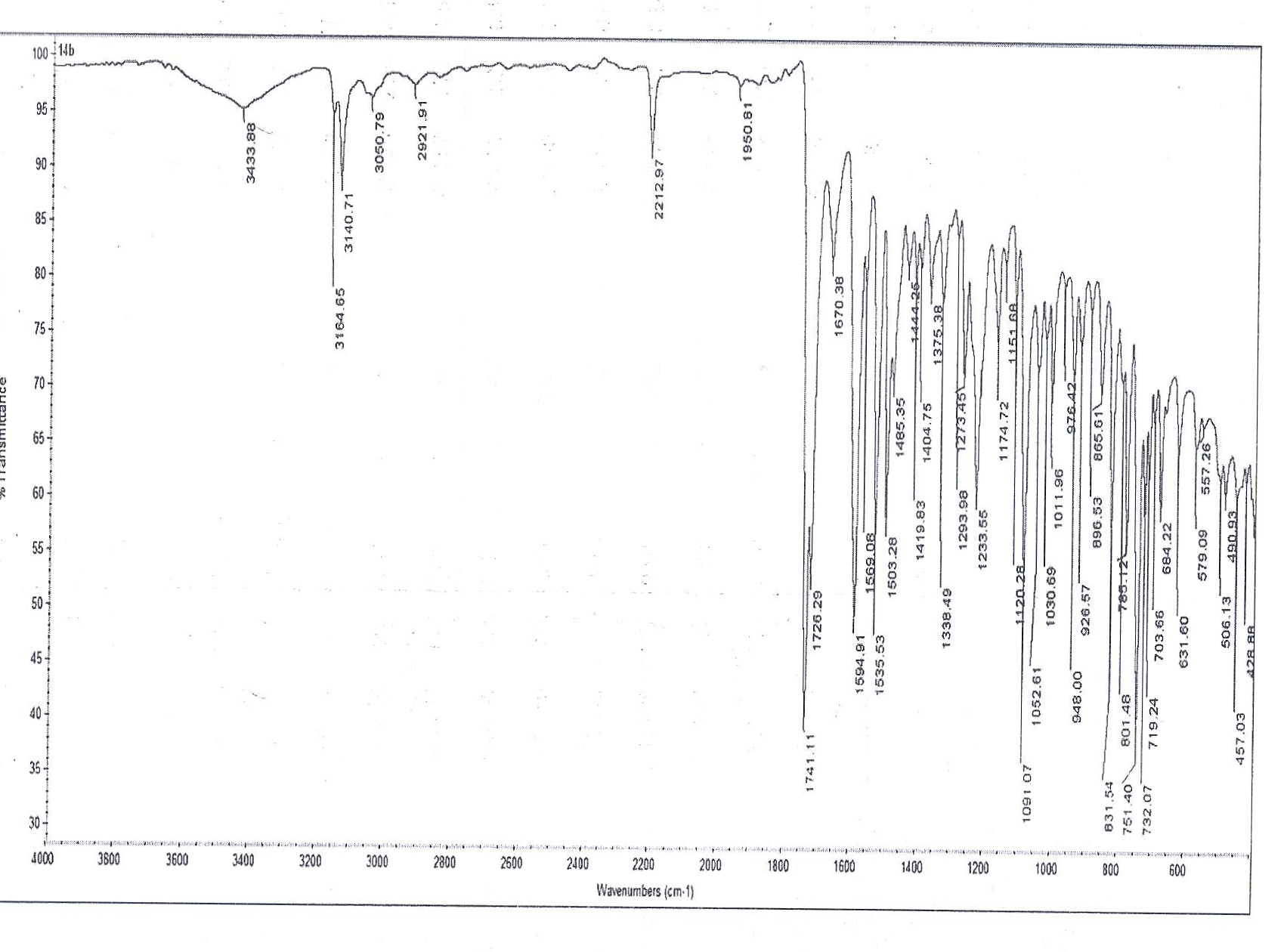
**

**S20. IR of compound 8c**

**
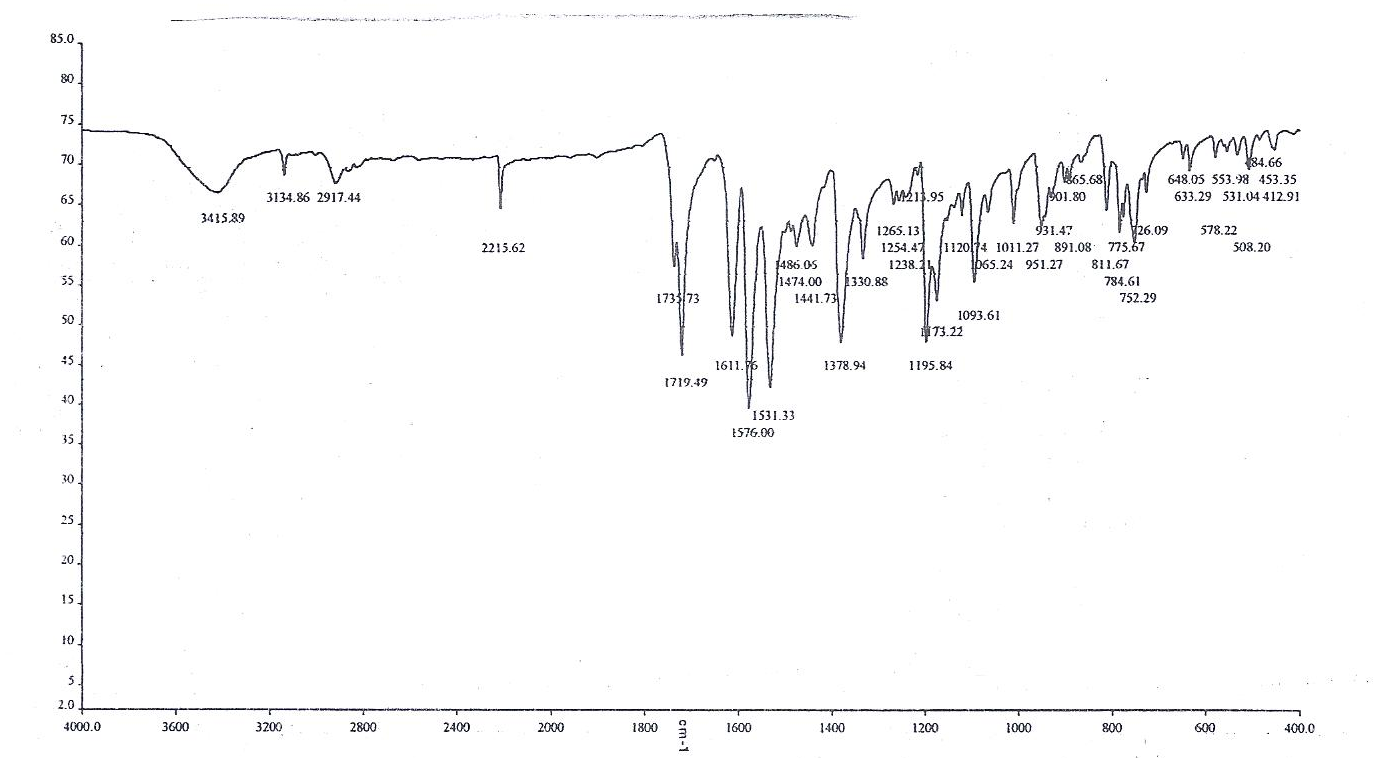
**

**S21. IR of compound 11a**

**
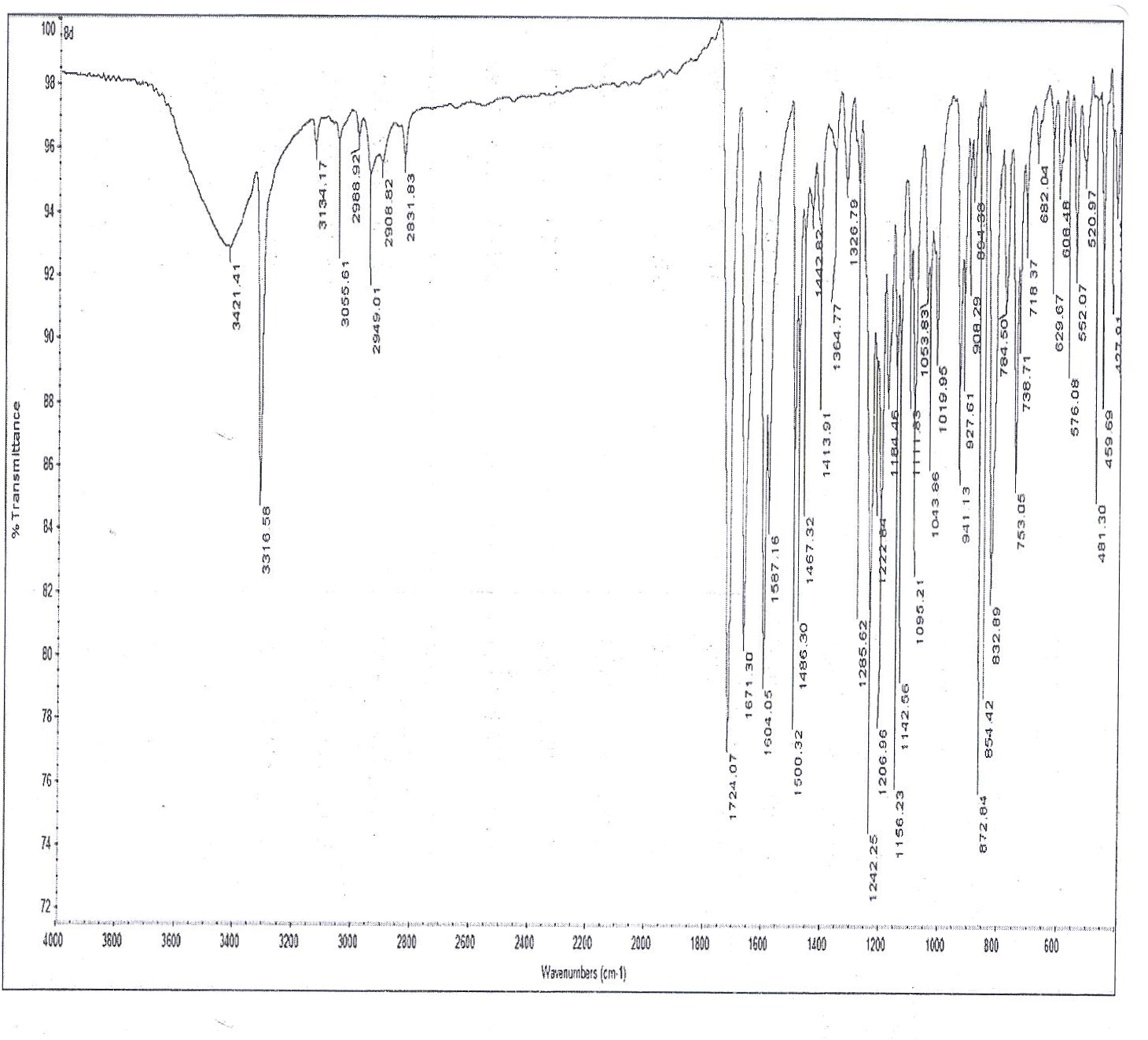
**

**S22. IR of compound 11b**

**
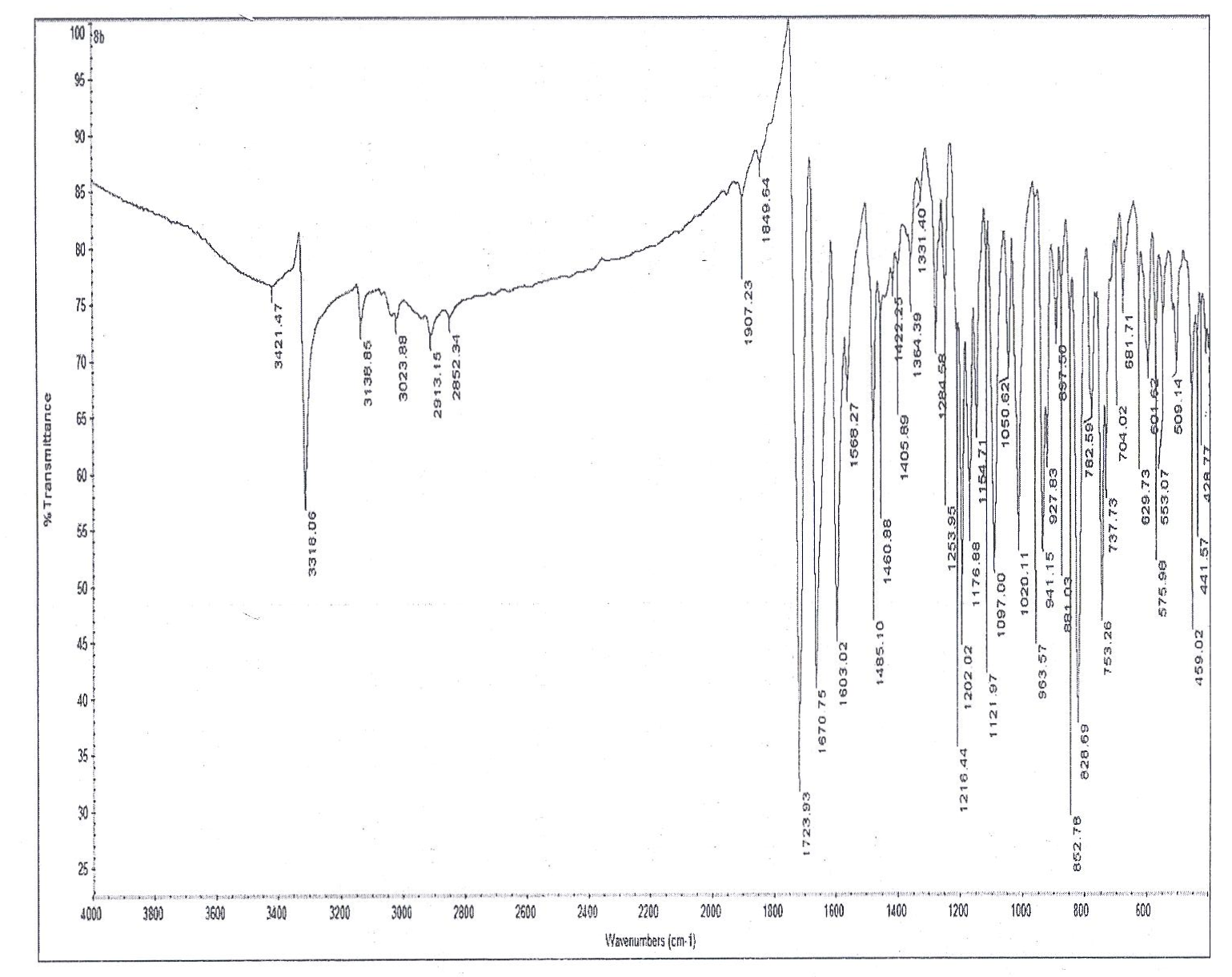
**

**S23. IR of compound 15a**

**
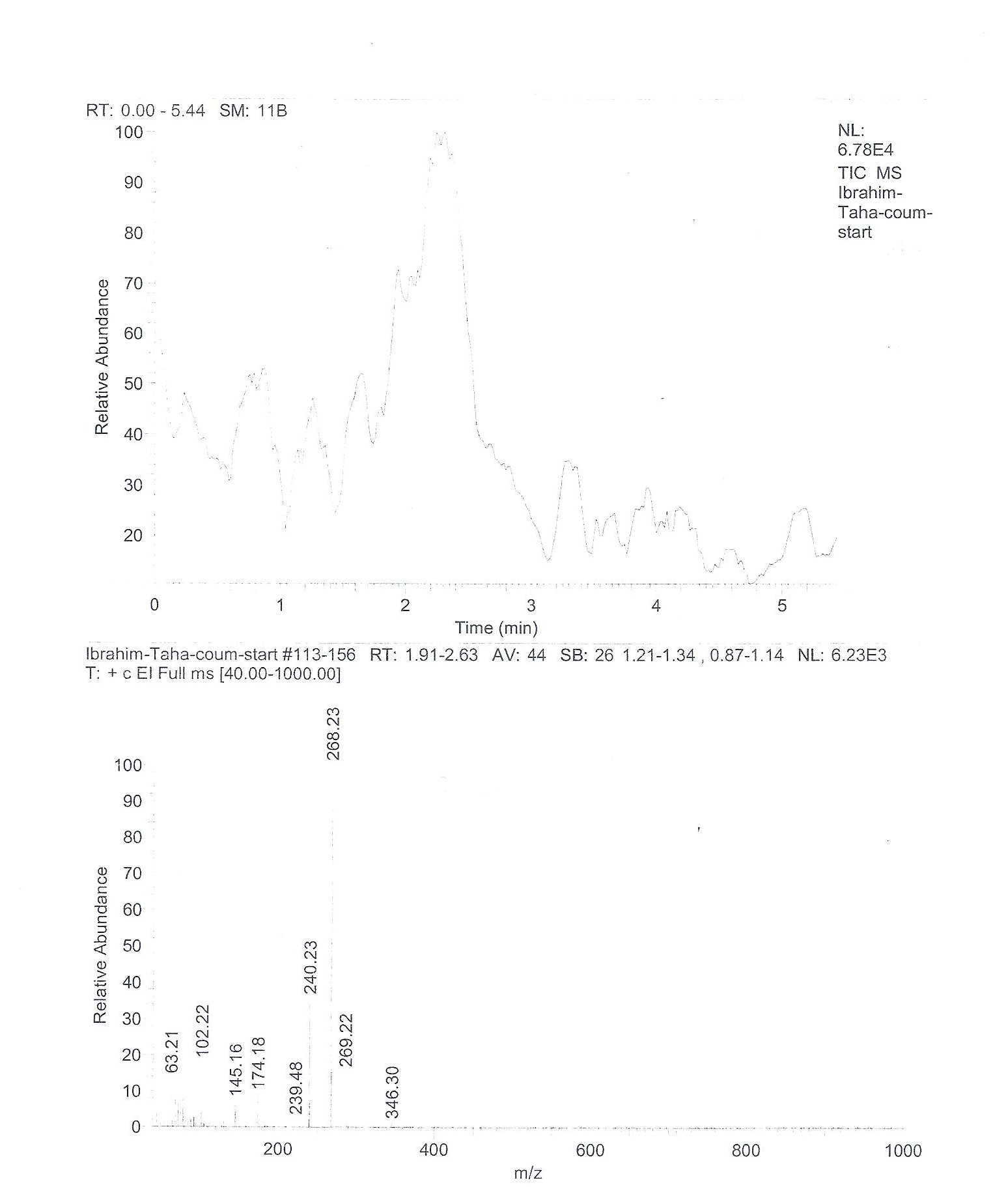
**

**S24. MS of compound 4**

**
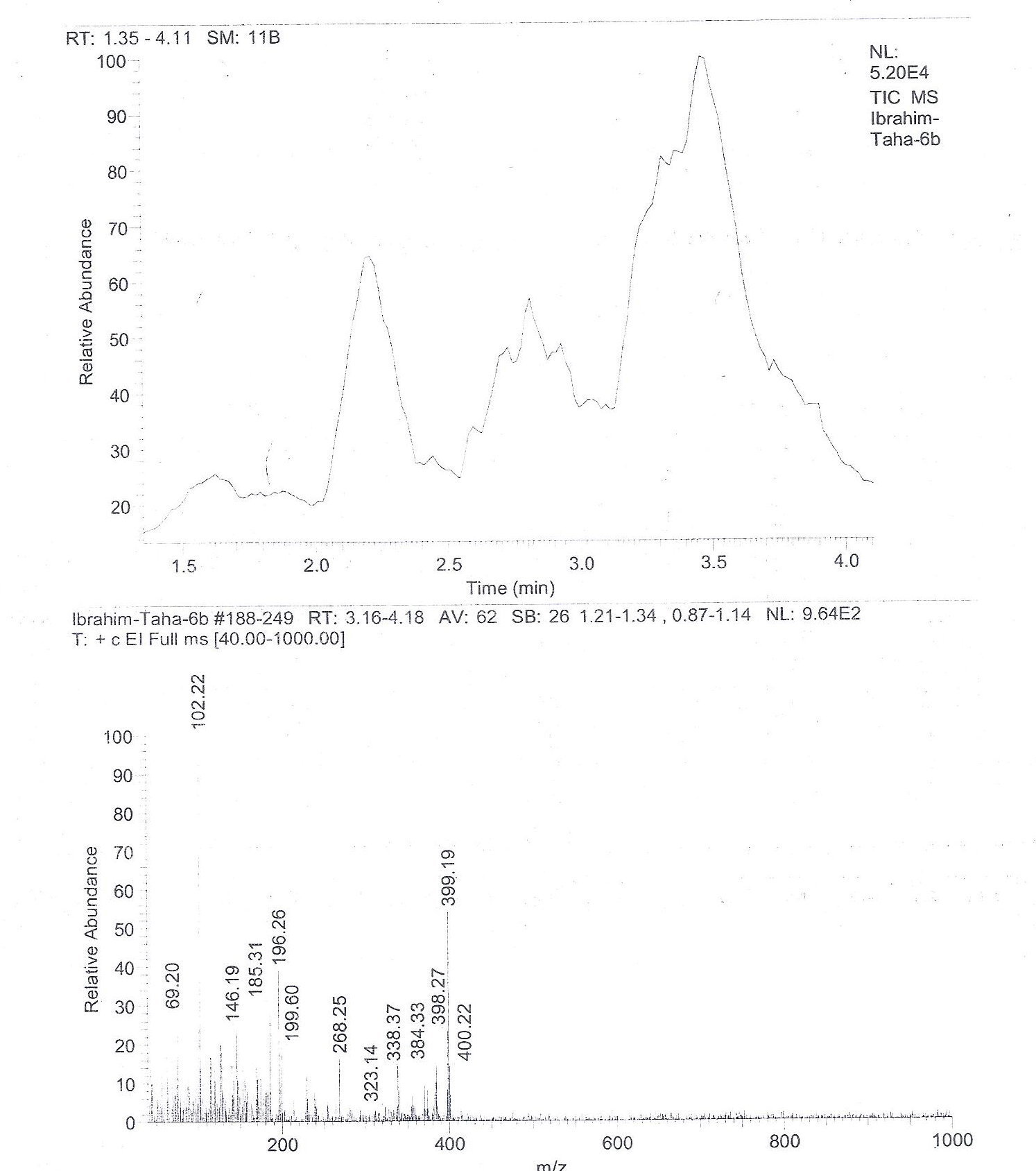
**

**S25. MS of compound 6b**

**
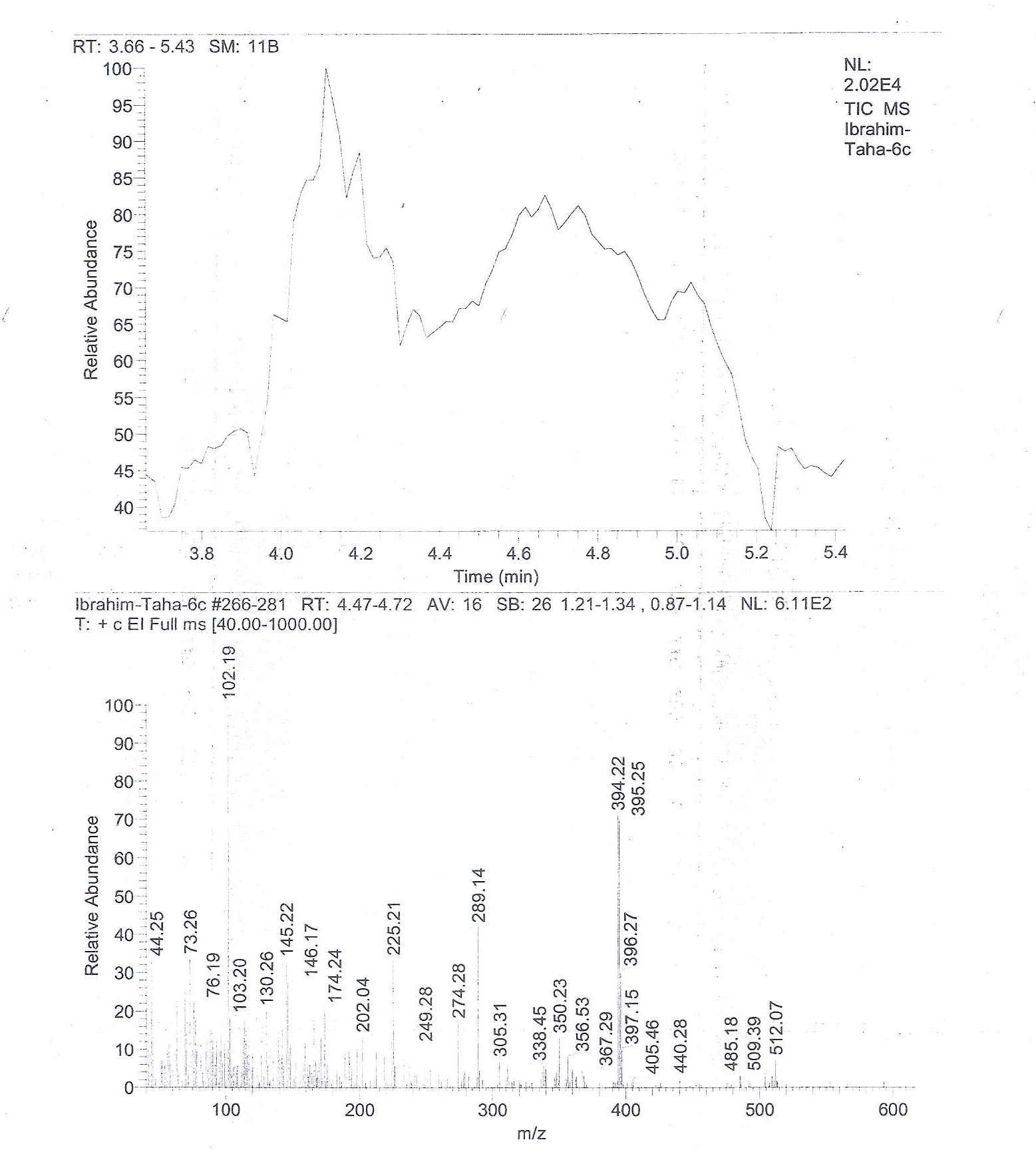
**

**S26. MS of compound 6c**

**
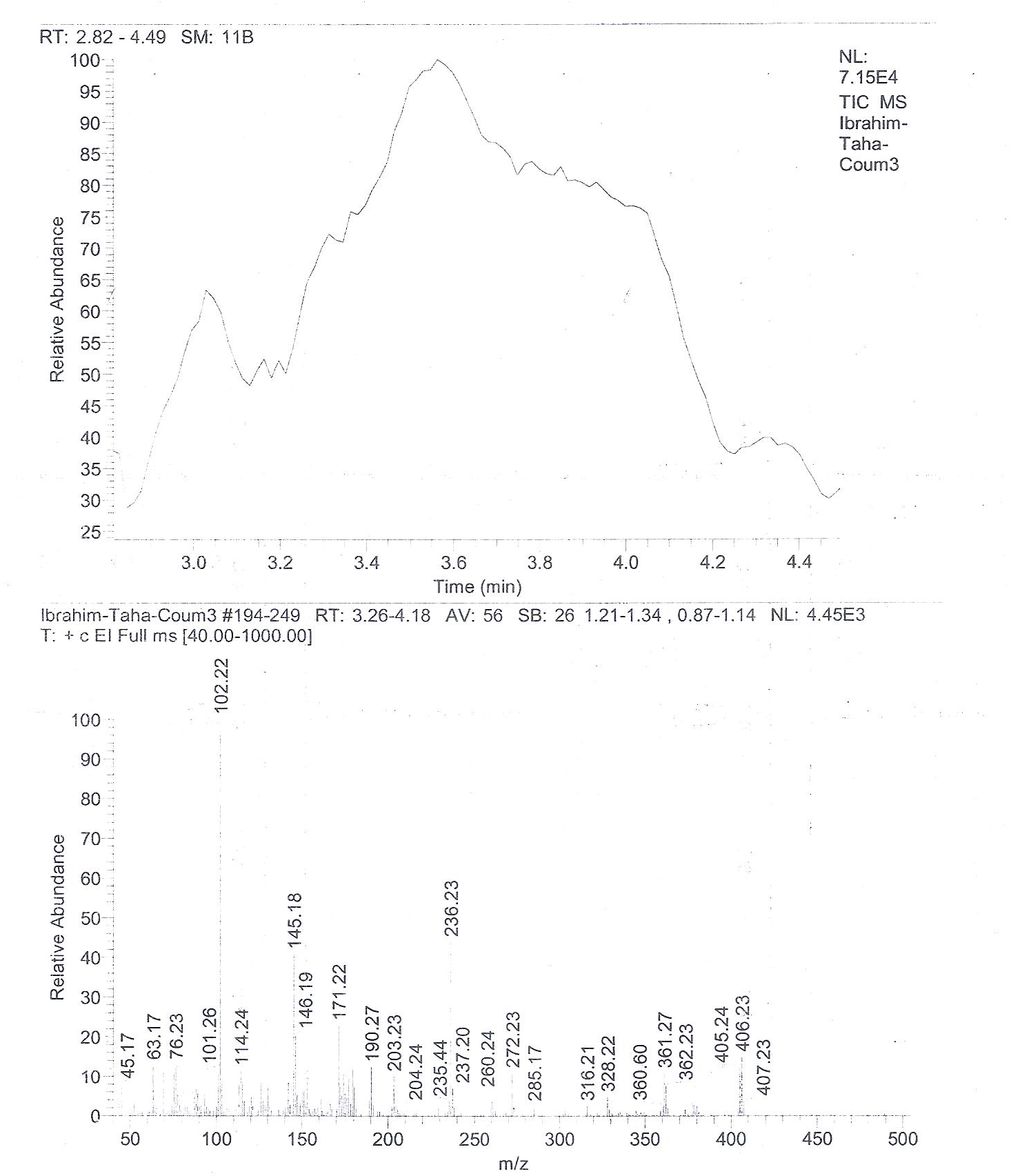
**

**S27. MS of compound 6d**

**
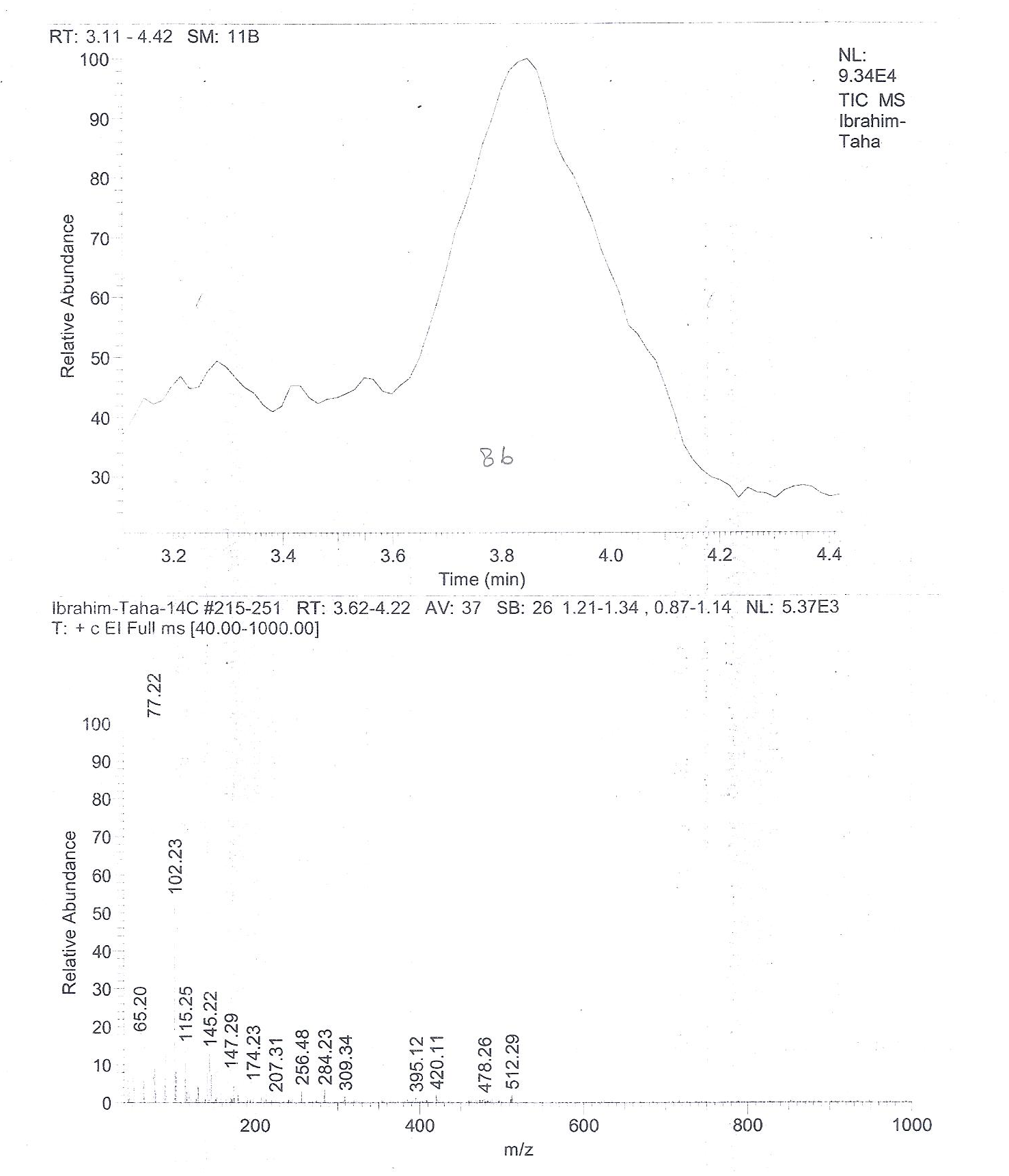
**

**S28. MS of compound 8b**

**
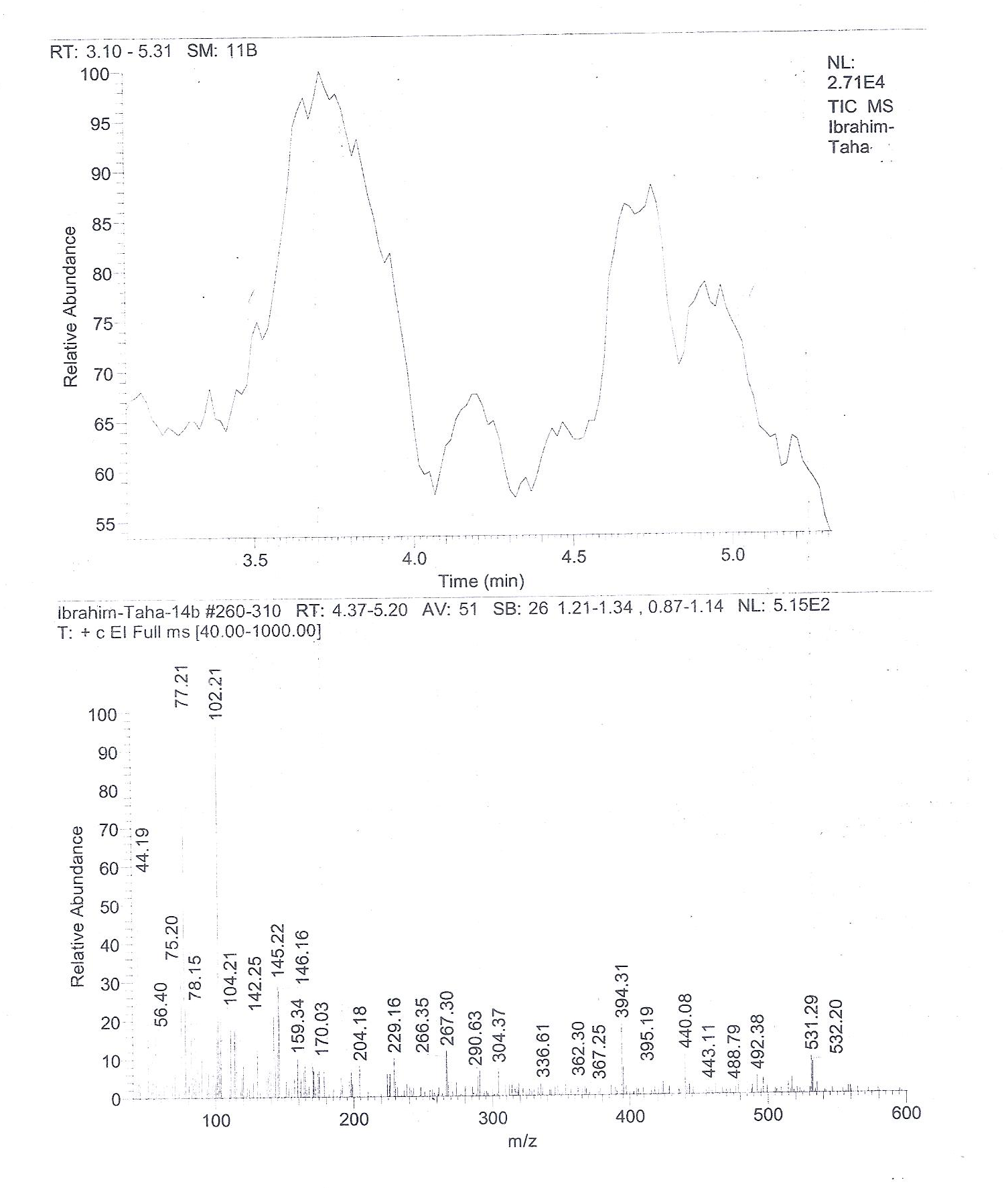
**

**S29. MS of compound 8c**

**
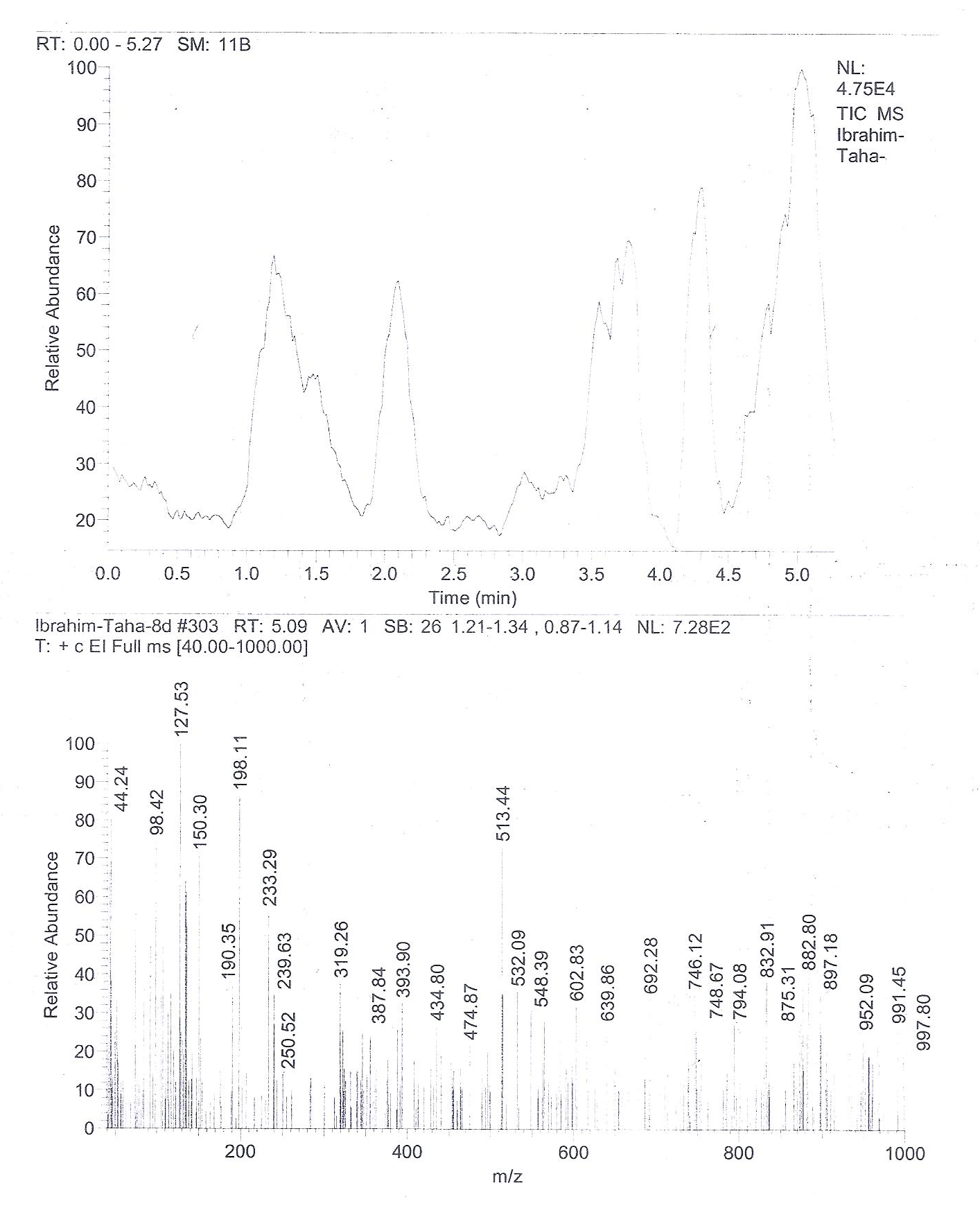
**

**S30. MS of compound 11b**

**
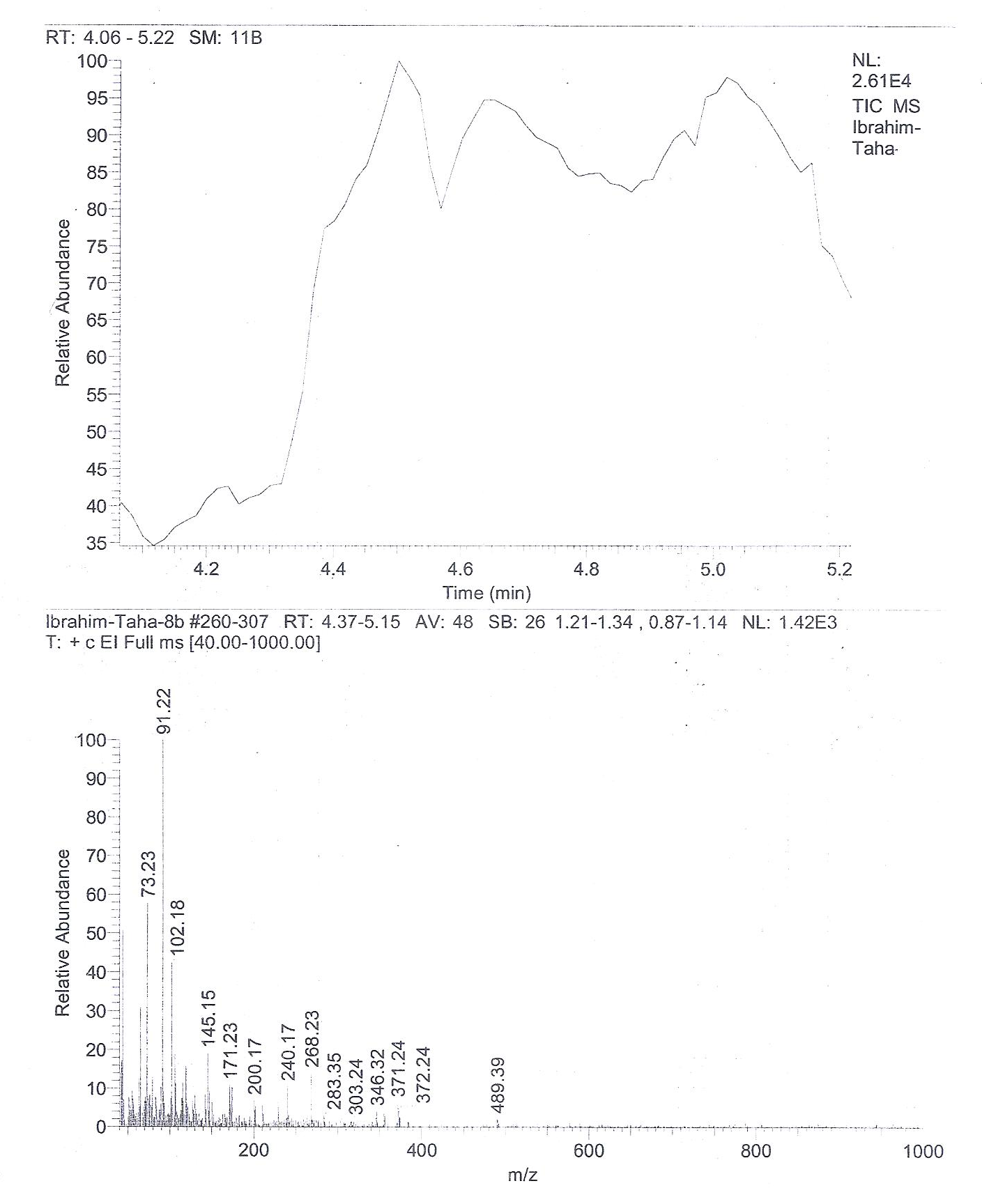
**

**S31. MS of compound 18b**

**Fig 32. Photo of antimicrobial**
